# Failure to track a stable AMOC state under rapid climate change

**DOI:** 10.1038/s41558-026-02730-w

**Published:** 2026-08-13

**Authors:** René M. van Westen, Reyk Börner, Henk A. Dijkstra

**Affiliations:** Department of Physics, Institute for Marine and Atmospheric research Utrecht, Utrecht University, Utrecht, the Netherlands

**Keywords:** Climate and Earth system modelling, Attribution, Physical oceanography, Projection and prediction

## Abstract

The Atlantic Meridional Overturning Circulation (AMOC), a tipping element of the climate system, currently has an estimated global warming threshold for collapse of +4.0 °C (uncertainty range 1.4–8 °C). However, such a threshold may not be meaningful because AMOC stability depends on the rate of radiative forcing change, not a set temperature. Here we identify an AMOC stabilizing mechanism that operates on timescales slower than present-day warming rates. Slow forcing permits coherent adjustment of surface and interior ocean properties, supported by enhanced evaporation and reduced sea-ice extent, counteracting destabilizing feedbacks. Using a slow CO_2_ ramp (+0.5 ppm yr^−1^) climate model simulation, we explicitly demonstrate the AMOC remains stable up to +5.5 °C of global warming. By contrast, under faster CO_2_ ramps, the AMOC collapses at substantially lower warming levels (+2 °C). Our findings demonstrate rate-induced AMOC tipping and imply that limiting the rate of emissions is critical for reducing the risk of an AMOC collapse.

## Abstract (web summary)

Climate change is expected to alter oceanic circulation, with the Atlantic Meridional Overturning Circulation (AMOC) at risk of collapse. This work shows that the stability of the AMOC is dependent on the rate of atmospheric CO_2_ change rather than having a fixed threshold.

## Main

Earth’s climate system contains various tipping elements in the biosphere, cryosphere and hydrosphere^1^. These tipping elements may undergo a transition upon exceeding a critical forcing, often expressed as global warming thresholds^2^. For the Atlantic Meridional Overturning Circulation (AMOC), the threshold for a collapse is estimated to be at global warming levels of +4 °C, with a range from +1.4 °C to +8.0 °C (ref. ^2^). In particular, the lower bound of such estimates is important for society to identify safe operating spaces that minimize the risk of disruptive climate change^3,4^.

However, estimating a critical warming threshold for the AMOC poses several challenges, from limited palaeo-climatic evidence and simulated tipping events to model biases causing large uncertainty in the AMOC response to radiative forcing^2,5–7^. A key underlying assumption behind such a threshold is that the present-day AMOC state will lose stability if the corresponding warming level is exceeded, triggering the onset of a collapse^1,2,8^. Combining this assumption with the future warming levels projected by models forced with the Shared Socioeconomic Pathways (SSPs), it seems possible to determine when an AMOC collapse would begin.

Yet, many results indicate that the evolution of the AMOC depends on the rate of radiative forcing change—in observations^9^, in conceptual climate models^10^, in early global climate models^11^ and recently in a low-resolution Earth system model^12^. In addition, modelling studies have found sustained strong AMOC states in very warm climates^13–16^, for example under doubled or quadrupled CO_2_ levels. While studies have emphasized the forcing rate effects on the magnitude of AMOC weakening^11,12^, the question whether the forcing rate alone can determine AMOC tipping has been less investigated. An early study, using a zonally averaged three-basin ocean model^10,17^, demonstrated that a faster rate of radiative forcing can indeed collapse the AMOC while a slower forcing rate does not. This is one of the first examples showing rate-dependent tipping^18,19^ of the AMOC, meaning that it fails to track its current stable state for sufficiently fast forcing change.

An additional problem with a warming threshold is that the main destabilizing feedback mechanism of the AMOC may operate independently of global temperatures. While certain tipping elements (for example, the Greenland Ice Sheet and West Antarctic Ice Sheet) are losing stability directly due to higher temperatures^20–23^, the AMOC is destabilized through the salt-advection feedback^24–27^. This feedback can be triggered by natural climate variability^28–31^ and thermal or haline changes in surface buoyancy fluxes over the North Atlantic Ocean^32–35^. Much progress has been made in assessing the stability properties of the AMOC with respect to varying surface freshwater forcing^36,37^, which in isolation requires unrealistically large freshwater inputs to collapse the present-day AMOC^7^. However, the present-day AMOC is exposed to substantial changes in radiative forcing, impacting both freshwater and heat forcings. This anthropogenic forcing is expected to substantially weaken the AMOC^38–40^ and possibly trigger a collapse^29,35,41^. Nonetheless, the interacting effects of changing heat and freshwater fluxes under global warming remain insufficiently understood.

Motivated by these issues with a global warming threshold for an AMOC collapse, we here investigate the radiative forcing path dependence of AMOC tipping in the Community Earth System Model (CESM, version 1.0.5) and several models participating in the Coupled Model Intercomparison Project (CMIP) phase 6. Starting from a recent quasi-equilibrium freshwater flux simulation with the CESM, in which an AMOC collapse has been found^33^, we extend earlier work on rate dependence in more detail^10,17^ and beyond AMOC weakening^11,12^. We demonstrate that sufficiently fast forcing can cause an AMOC collapse without crossing a tipping threshold in CO_2_ or warming levels, questioning the suitability of global warming thresholds for assessing AMOC tipping risk.

## Stable AMOC under extreme climate change

We will present an in-depth analysis of a slow CO_2_ ramp simulation (+0.5 ppm yr^−1^) with the CESM, together with a comparison using faster CO_2_ ramps (+2.5 ppm yr^−1^ and +5.0 ppm yr^−1^) and also different Representative Concentration Pathway (RCP) scenarios; the results for the RCP scenarios were already presented previously^34^. All climate model simulations are initialized from a quasi-equilibrium hosing simulation performed under constant pre-industrial (PI) radiative forcing conditions^33,42^. This PI hosing simulation reveals a bistable AMOC regime, consistent with evidence suggesting that the present-day AMOC resides in such a regime^37^.

**Fig. 1:**
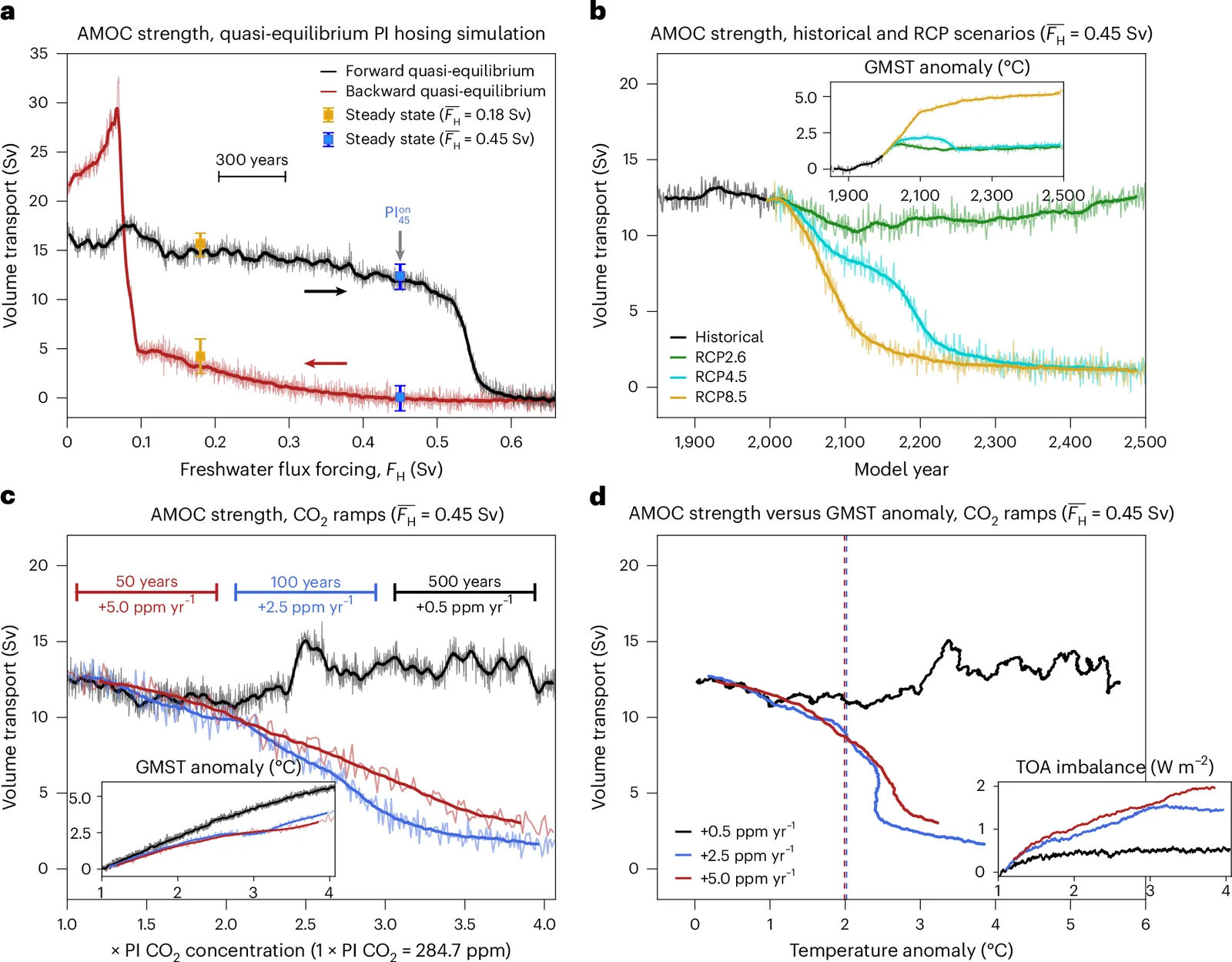
Climate model simulations with the CESM. **a**, The AMOC strength (at 26° N and 1,000 m depth) for the quasi-equilibrium PI hosing simulation, including four steady states (error bars indicate the mean value, including minimum and maximum values of last 50 model years). **b**, The AMOC strength for the historical and three extended RCP scenarios and $\overline{{F}_{{\rm{H}}}}=0.45\,\mathrm{Sv}$. Inset: the GMST anomaly compared with the historical period 1850–1899. **c**,**d**, The AMOC strength for the different CO_2_ ramps and ${\overline{{F}_{\rm{H}}}}=0.45\,\mathrm{Sv}$ against CO_2_ concentration (**c**) and against GMST anomaly (**d**) (compared with ${{\rm{PI}}}_{45}^{{\rm{on}}}$), including the corresponding GMST values of AMOC collapse onset (Extended Data Table 1). Insets: the GMST anomaly (**c**) and the TOA radiative imbalance (**d**), with horizontal axes in units of PI CO_2_ concentration. In all panels, the thin curves are yearly averages, whereas the thick curves are smoothed versions (25-year moving averages).

In the quasi-equilibrium PI hosing simulation (Fig. 1a), an additional freshwater flux (*F*_H_) was gradually applied over the North Atlantic Ocean (20° N–50° N) and this forcing was compensated elsewhere (at the ocean surface) to conserve ocean salinity. Qualitatively comparable responses are found under small variations in hosing location^25,32,43^ and in the applied compensation protocol^44–46^, but both choices do influence quantitative thresholds. For this CESM version and protocol, a tipping point (that is, a saddle-node bifurcation) is located at *F*_H_ ≈ 0.5 Sv (ref. ^7^). The existence of a tipping point in freshwater forcing is consistent with results across a hierarchy of climate models^5,24,47,48^. Note that the AMOC tipping threshold is found at large *F*_H_ values, about 65 times the present-day melt rate of the Greenland Ice Sheet^33^, which is related to persistent climate model biases^45,49,50^. The real AMOC is probably closer to a tipping point^7,37^, and an imposed background hosing should be regarded as a bias correction for AMOC stability.

Statistical equilibria (that is, attracting steady states with time-invariant statistics) were obtained at multiple hosing strengths by branching off at selected values of *F*_H_ and integrating for 500 years at fixed ${F}_{H}=\overline{{F}_{H}}$ (refs. ^7,30^). The steady states have a radiative imbalance close to zero, meaning that any remaining model drift is smaller than internal climate variability^51^, and are also shown in Fig. 1a for $\overline{{F}_{{\rm{H}}}}=0.18\,\mathrm{Sv}$ and $\overline{{F}_{{\rm{H}}}}=0.45\,\mathrm{Sv}$. In the following, we will use the PI steady state with a strong AMOC at $\overline{{F}_{{\rm{H}}}}=0.45\,\mathrm{Sv}$ as a reference, which we refer to as ${{\rm{PI}}}_{45}^{{\rm{on}}}$ (last 50 model years). This reference state lies in the destabilizing regime of the salt-advection feedback and is hence more prone to AMOC transitions than at $\overline{{F}_{{\rm{H}}}}=0.18\,\mathrm{Sv}$ (ref. ^7^). While reducing freshwater biases, this choice also implies a weaker AMOC strength (~13 Sv) than in observations (~17 Sv)^52,53^.

Different radiative forcing simulations were branched from the end of ${{\rm{PI}}}_{45}^{{\rm{on}}}$, keeping $\overline{{F}_{H}}$ fixed. The historical forcing (1850–2005) was followed by three RCP (2006–2100) simulations. These RCP scenarios were subsequently continued to model year 2,500 under their fixed greenhouse gas and aerosol concentrations of the year 2100 (Fig. 1b). This was done to determine statistical equilibria reached after different climate change scenarios^34^. The AMOC recovers under RCP2.6, while the AMOC collapses under RCP4.5 and RCP8.5. Note that this does not guarantee that the AMOC will remain in a collapsed state beyond 2500 under these fixed climate change conditions^13–15,29^. Consequently, it remains unclear whether the AMOC crosses a saddle-node bifurcation under radiative forcing increase, while strong evidence points to this being the case under increasing *F*_H_ (ref. ^7^) (Fig. 1a). Hence, we avoid the term ‘tipping point’ and instead refer to transitions to a persistently weak alternative state as ‘AMOC collapse’. We determine the timing of the collapse onset using modern AMOC theory (Methods; Extended Data Table 1). Even if the AMOC would eventually recover under fixed climate change conditions, the AMOC-induced impacts are considerable over the 300-year period (model years 2,200–2,500) with very weak AMOC strengths^51,54^.

For RCP4.5 (RCP8.5), the collapse onset occurs around model year 2,110 (2,060), when the global mean near-surface temperature (GMST) anomaly is +2.19 °C (+2.78 °C) compared with the historical period of 1850–1899^34^. The GMST anomaly difference of 0.59 °C between RCP4.5 and RCP8.5 highlights a dependence on the radiative forcing agent (for example, CO_2_, methane and aerosols) pathways that are relevant for AMOC responses^55,56^, which was previously demonstrated for a low-resolution version of the CESM^12^. Note that AMOC tipping under radiative forcing is also dependent on the background $\overline{{F}_{H}}$, as the AMOC recovers under RCP4.5 and $\overline{{F}_{{\rm{H}}}}=0.18\,\mathrm{Sv}$ (ref. ^34^); this simulation will also be analysed below.

To further test the sensitivity of estimated warming thresholds for an AMOC collapse onset, we performed three different CO_2_ ramp simulations (again at fixed $\overline{{F}_{{\rm{H}}}}=0.45\,\mathrm{Sv}$), where the CO_2_ concentration increases from the PI level of 284.7 ppm (= 1 × PI CO_2_) at a certain rate. We choose a linear increase instead of the more common approach using exponential growth rates (for example, ref. ^12^). This avoids very high CO_2_ levels (22 × PI CO_2_) and large CO_2_ growth rates (>100 ppm yr^−1^) in our multicentennial simulations. Starting from the end of ${{\rm{PI}}}_{45}^{{\rm{on}}}$, we linearly increased the CO_2_ concentration up to 4.07 × PI CO_2_ (Fig. 1c,d) with rates of +0.5 ppm yr^−1^ (black curve, 1,750 model years), +2.5 ppm yr^−1^ (blue curve, 350 model years) and +5.0 ppm yr^−1^ (red curve, 175 model years). For reference, the current CO_2_ rate of increase at the Mauna Loa Observatory is +2.6 ppm yr^−1^ (2015–2025 average).

The AMOC collapses for both the +2.5 ppm yr^−1^ and +5.0 ppm yr^−1^ cases with an associated GMST anomaly of +2.02 °C and +1.99 °C compared with ${{\rm{PI}}}_{45}^{{\rm{on}}}$, respectively (Extended Data Table 1). By contrast, in the slow ramp of +0.5 ppm yr^−1^, the AMOC remains stable far above this apparent +2 °C threshold, reaching +5.5 °C over the last 50 model years. This is an important indication that AMOC tipping under global warming can depend solely on the forcing rate, for which the trajectories fail to track a stable equilibrium under fast radiative forcing changes. This is corroborated by the radiative imbalance at the top of atmosphere (TOA; Fig. 1d, inset), where TOA remains stable at +0.5 W m^−2^ for the slow ramp, meaning that the climate system can reasonably track the shifting equilibrium under global warming. For the faster ramps, the TOA imbalance increases throughout the simulations, indicative of a climate system that is departing further from equilibrium. Note that the warming level up to 2 × PI CO_2_ (Fig. 1c, inset), for which all cases still exhibit a relatively strong AMOC, is larger for slower rates owing to the fact that the climate system has more time to warm, allowing slow amplifying climate feedbacks (for example, sea-ice melt) to contribute. The warming rates (up to 2 × PI CO_2_) are of course faster in +5.0 ppm yr^−1^ (+2.95 °C per century) than in +0.5 ppm yr^−1^ (+0.39 °C per century).

## Density changes under slow warming

In the following, we examine the slow CO_2_ ramp simulation (+0.5 ppm yr^−1^) in greater detail to understand why the AMOC remains stable under extreme global warming. For convenience, we mostly report the slow CO_2_ ramp in model years rather than in units of PI CO_2_.

**Extended Data Fig. 1:**
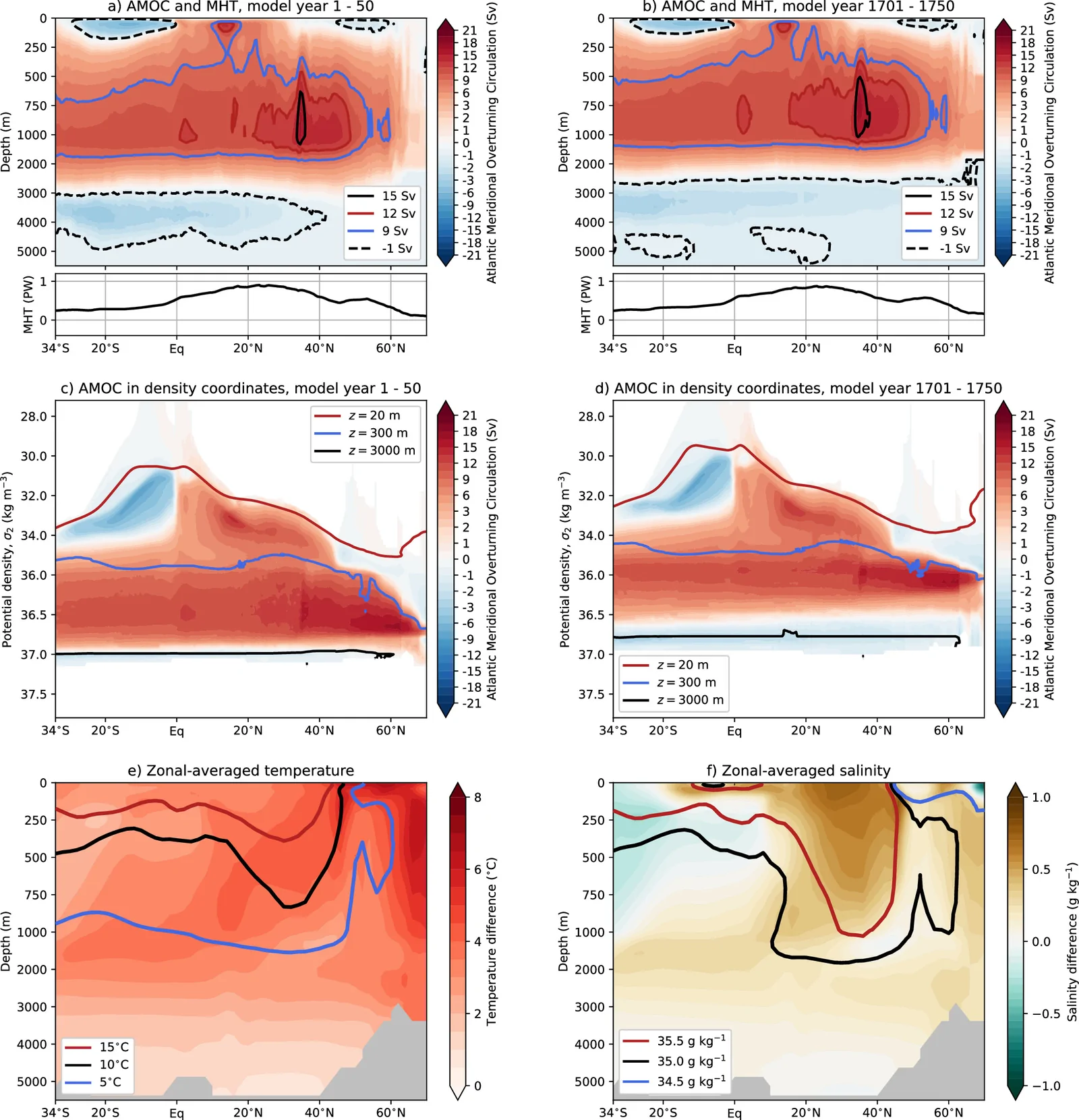
Atlantic Ocean Responses for the slow CO_2_ ramp. (**a** & **b**): The time-mean AMOC in depth coordinates for model years **a**) 1 – 50 and **b**) 1701 – 1750. The lower panel shows the meridional heat transport (MHT). (**c** & **d**): The time-mean AMOC in density coordinates for model years **c**) 1 – 50 and **d**) 1701 – 1750. The three curves represent the (section-averaged) depth level, whereas the 20 m depth contour is smoothed to reduce its meridional variability. (**e**): The zonally-averaged temperature difference between model years 1701 – 1750 and ${{\rm{PI}}}_{45}^{{\rm{on}}}$ (shading). The curves are three isotherms of ${{\rm{PI}}}_{45}^{{\rm{on}}}$. (**f**): Similar to panel **e**, but now for the zonally-averaged salinity.

The AMOC in depth coordinates and meridional heat transport for model years 1–50 (≈ 1 × PI CO_2_) and 1701–1750 (≈ 4 × PI CO_2_) are shown in Extended Data Fig. 1a,b, respectively. The overall overturning structures and meridional heat transports do not change much, although the depth of the northward overturning cell becomes smaller from about 2,800 m depth (model years 1–50) to about 2,400 m depth (model years 1,701–1,750). The AMOC in density coordinates shifts to lighter water classes between the two periods (Extended Data Fig. 1c,d), with the section-averaged depth levels of 20 m, 500 m and 3,000 m getting lighter by about 0.90 kg m^−3^, 0.65 kg m^−3^ and 0.25 kg m^−3^, respectively. The relatively light water masses near the surface are becoming lighter more rapidly than the relatively heavy water masses at depth, which is related to the greater warming closer to the surface (Extended Data Fig. 1e). The salinity increases over most parts of the Atlantic Ocean (Extended Data Fig. 1f), which makes water masses more dense and, hence, partially compensates the warming-induced response of the density.

**Fig. 2:**
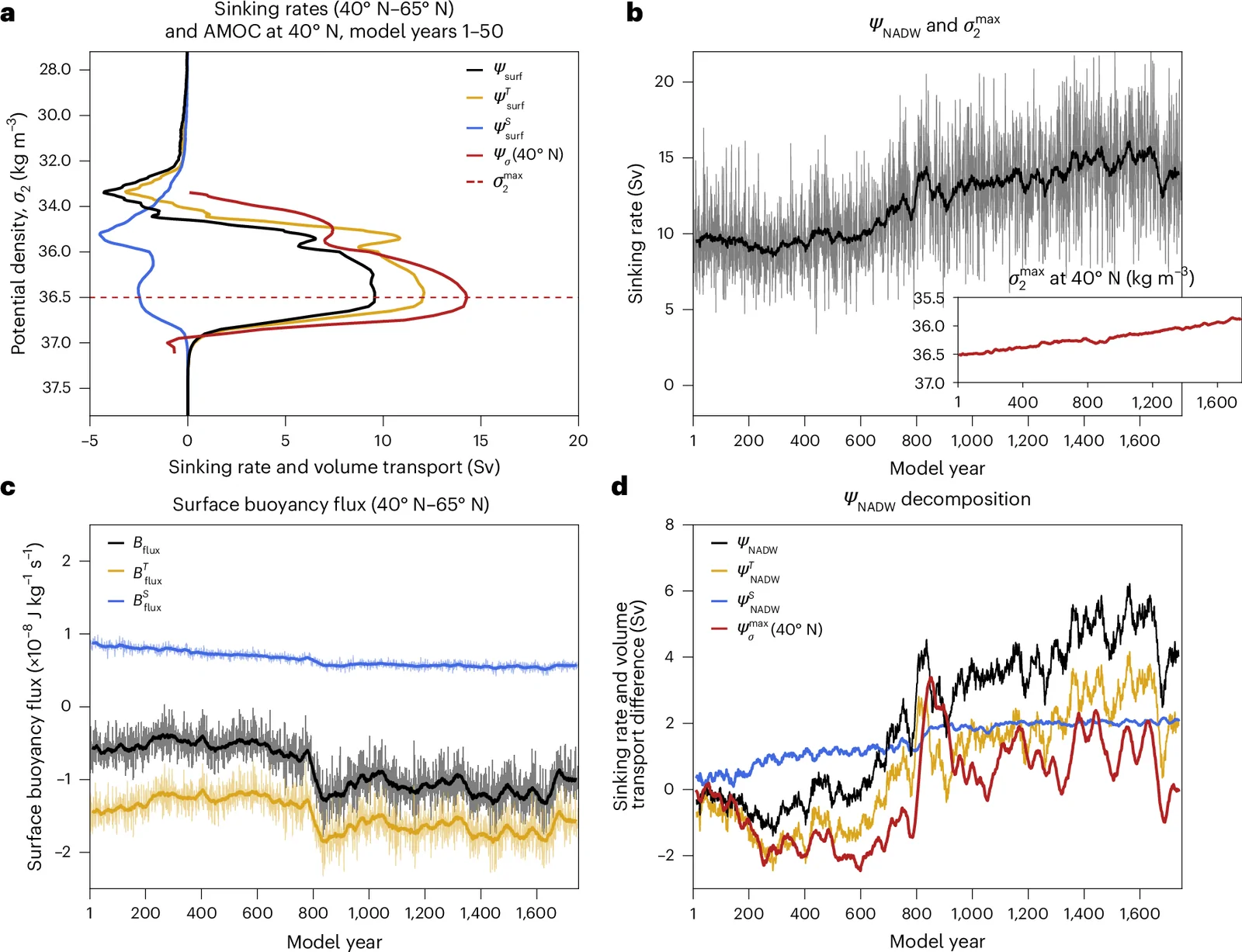
Surface-forced AMOC for the slow CO_2_ ramp. **a**, The surface-forced AMOC between 40° N and 65° N (*Ψ*_surf_, sinking rates) over the first 50 model years, including the temperature and salinity contributions. The AMOC at 40° N in density coordinates with its maximum indicated by the dashed red line (${\sigma }_{2}^{\max }$) are also shown. **b**, The *Ψ*_NADW_ and ${\sigma }_{2}^{\max }$ (inset). **c**, The spatially averaged (40° N and 65° N) surface buoyancy flux, decomposed into the heat and freshwater fluxes. **d**, The *Ψ*_NADW_ differences compared with ${{\rm{PI}}}_{45}^{{\rm{on}}}$, including the temperature and salinity contributions. The differences for the maximum AMOC strength at 40° N are also shown. The time series in **b**–**d** are smoothed through a 25-year running mean (to reduce the variability).

Water mass transformation (WMT; Methods) primarily takes place over the isopycnal outcropping region (here defined as 40° N–65° N) in the North Atlantic Ocean, which is crucial for sustaining an adiabatic AMOC^57,58^. Under the assumptions of thermal wind balance^27^ and that WMT mainly takes place near the surface, the adiabatic AMOC can be reconstructed from surface buoyancy fluxes^59–62^. Indeed, the AMOC at 40° N in density coordinates (*Ψ*_*σ*_) and the surface-forced AMOC between 40° N and 65° N (*Ψ*_surf_ = *Ψ*_WMT_(40° N)−*Ψ*_WMT_(65° N)) closely resemble each other (Fig. 2a, red and black curves, respectively). The surface-forced AMOC is mainly driven by oceanic heat loss to the atmosphere, whereas freshwater fluxes have a limited contribution (Fig. 2a, yellow and blue curves), consistent with observations^63^. Surface-induced WMT rates that contribute to the water supply of the North Atlantic Deep Water (NADW) are found for density classes of ${\sigma }_{2}\ge {\sigma }_{2}^{\max }$ (ref. ^34^): ${\varPsi }_{\mathrm{surf}}({\sigma }_{2}^{\max })-{\varPsi }_{\mathrm{surf}}({\sigma }_{2}^{\infty })$. Here, ${\sigma }_{2}^{\max }$ is the density level of the maximum AMOC strength at 40° N (Fig. 2a, dashed red line) and ${\varPsi }_{{\rm{surf}}}({\sigma }_{2}^{\infty })=0$. Hence, the reconstructed AMOC strength from surface buoyancy fluxes is then defined as ${\varPsi }_{\mathrm{NADW}}(t)={\varPsi }_{\mathrm{surf}}({\sigma }_{2}^{\max }(t))$, which can also be decomposed into a thermal (${{\varPsi }_{\mathrm{NADW}}^{T}}$) and haline (${\varPsi }_{\mathrm{NADW}}^{S}$) contribution. Note that *Ψ*_NADW_ approximates the actual AMOC strength (Fig. 2d), because *Ψ*_NADW_ does not consider subsurface mixing and volume tendencies^64^. WMT contributions outside the 40° N–65° N latitude band are relatively small^34^.

**Fig. 3:**
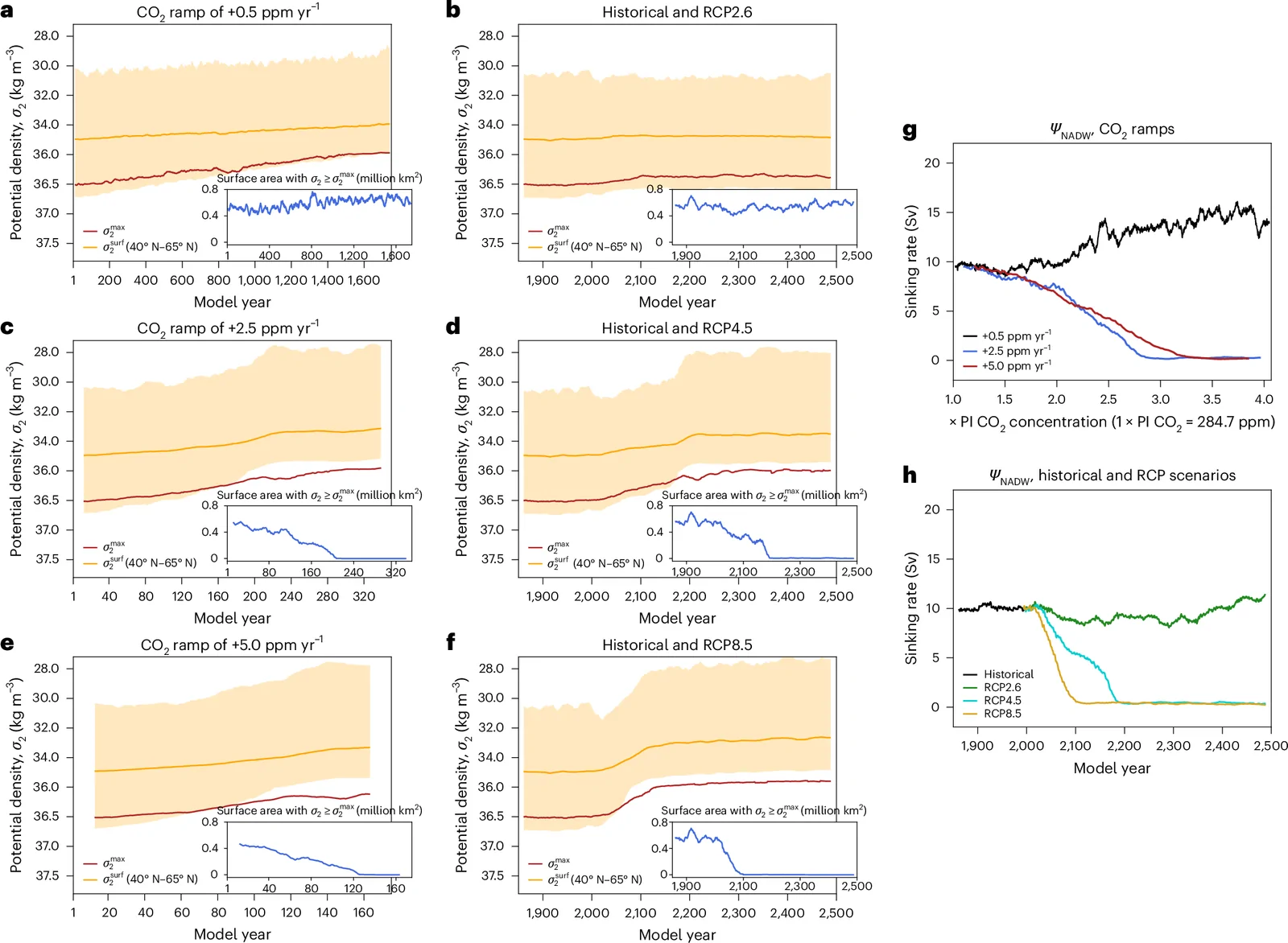
Atlantic Ocean potential densities. **a**–**f**, The potential density in the North Atlantic Ocean for the surface (${\sigma }_{2}^{{\rm{surf}}}$, 40° N–65° N, yellow curve) and at the maximum AMOC strength at 40° N (${\sigma }_{2}^{\max }$, red curve). The yellow shading represents the variations in surface potential density, where for each month the lightest and heaviest potential densities (over 40° N–65° N) are retained and are subsequently converted to yearly averages. Insets: the surface area where the monthly surface potential densities are heavier than ${\sigma }_{2}^{\max }$, which is subsequently converted to yearly averages. The results are for the CO_2_ ramps (**a**,**c**,**e**) and historical and RCP scenarios (**b**,**d**,**f**); all have $\overline{{F}_{{\rm{H}}}}=0.45\,\mathrm{Sv}$. **g**,**h**, The *Ψ*_NADW_ for the CO_2_ ramps (in units of PI CO_2_) (**g**) and historical and RCP scenarios (**h**). All time series are smoothed through a 25-year running mean to reduce the variability.

The quantity *Ψ*_NADW_ is useful as near-zero values indicate that an adiabatic AMOC cannot be sustained^34^. The *Ψ*_NADW_ remains relatively large throughout the slow CO_2_ ramp (Fig. 2b), which is expected given that the AMOC tracks the shifting equilibrium under global warming. To understand this *Ψ*_NADW_ response, we first analyse the ${\sigma }_{2}^{\max }$ (Fig. 2b, inset), which is shifting to lighter density classes. To keep the adiabatic pathways open, the surface density in the sinking region (40° N–65° N) needs to change at a comparable rate as ${\sigma }_{2}^{\max }$, which is indeed the case (Fig. 3a). The retreating sea-ice cover further activates additional sinking regions over the first 800 model years (Fig. 3a, inset), which will be discussed in more detail below. This coherent surface and interior response is found for the RCP2.6 simulation (Fig. 3b). However, for the faster CO_2_ ramps, as well as RCP4.5 and RCP8.5 simulations (Fig. 3c–f), the surface sinking region is getting lighter faster than ${\sigma }_{2}^{\max }$, and this closes the adiabatic pathways. For these AMOC collapse cases, near-zero values of *Ψ*_NADW_ (Fig. 3g,h) happen when the AMOC strength is strongly reduced (⪅5 Sv). This demonstrates that rate-dependent effects are important not only for AMOC weakening^12^ but also for AMOC collapse scenarios.

The *Ψ*_NADW_ remains sufficiently large and even increases during the slow CO_2_ ramp simulation. It would, by construction, remain unchanged under a uniform shift in the ocean’s potential density, meaning that *Ψ*_NADW_ variations are then linked to surface buoyancy fluxes changes (*B*_flux_; Fig. 2c). In the slow CO_2_ ramp, the quantities *Ψ*_NADW_ and *B*_flux_ are closely related (*R*^2^ = 0.896), but note that a part of *B*_flux_ does not supply water to the adiabatic AMOC pathways. Both surface heat fluxes (${B}_{{\rm{flux}}}^{T}$) and surface freshwater fluxes (${B}_{{\rm{flux}}}^{S}$) decline over time, resulting in larger ${\Psi }_{{\rm{NADW}}}^{T}$ and ${\Psi }_{\mathrm{NADW}}^{S}$, respectively (Fig. 2d). The initial AMOC weakening during the first 300 model years is attributed to changing heat fluxes, whereas freshwater fluxes have the opposite response.

**Extended Data Fig. 2:**
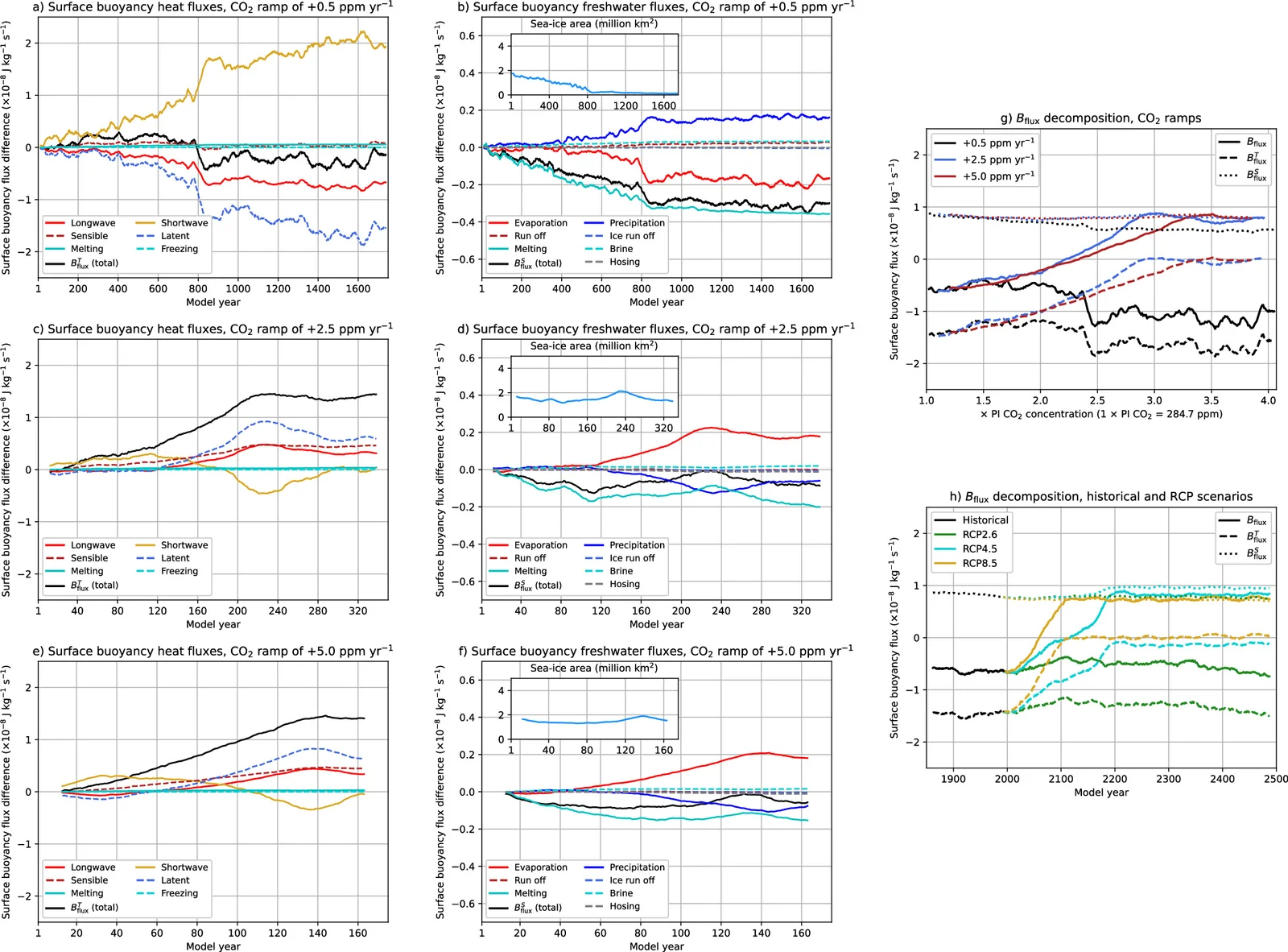
Surface buoyancy flux decomposition. (**a** – **f**): The surface buoyancy flux differences (compared to ${{\rm{PI}}}_{45}^{{\rm{on}}}$) between 40^∘^N – 65°N for the three CO_2_ ramps, decomposed into the different heat (left column) and freshwater (middle column) fluxes. The insets shows the yearly-averaged sea-ice area (grid cells with sea-ice fractions of at least 15%) between 40°N – 65°N. (**g** & **h**): The *B*_flux_ decomposition for the CO_2_ ramps (in units of PI CO_2_) and historical and RCP scenarios. All time series are smoothed through a 25-year running mean to reduce the variability.

The *B*_flux_ framework also allows identifying stabilizing and destabilizing factors. For the surface heat fluxes in the slow CO_2_ ramp (Extended Data Fig. 2a), the dominant contributions come from more outgoing longwave radiation (stabilizing) due to higher sea surface temperatures, more latent heat release (stabilizing) due to enhanced evaporation rates, and more shortwave absorption (destabilizing) due to a lower albedo. The North Atlantic sea-ice extent retreats northwards under higher temperatures (Extended Data Fig. 2b, inset) and this limits sea-ice insulation effects that tend to destabilize the AMOC^30,65^. Another stabilizing sea-ice effect is the reduced melting contribution to the surface freshwater flux (Extended Data Fig. 2b), with the sea-ice contribution also being rate dependent^12,66^. This sea ice forms around Greenland and by advection ends up in the 40° N–65° N latitude band, where it melts during boreal spring^27^. Enhanced evaporation (stabilizing) and precipitation (destabilizing) also have a substantial contribution, but their opposing effects compensate to about zero. The remaining *B*_flux_ components have a relatively small contribution.

Almost all *B*_flux_ components have the opposite response in the faster CO_2_ ramps (Extended Data Fig. 2c–f) compared with the slow CO_2_ ramp; the faster CO_2_ ramp responses are very similar to those under RCP4.5 and RCP8.5 (for the RCPs, see refs. ^34,67^ or online material). Recent work suggests that a sign change in *B*_flux_ (from negative to positive) can be used to indicate the onset of the AMOC collapse^34,68^, which is indeed the case for the faster CO_2_ ramps, RCP4.5 and RCP8.5 (Extended Data Fig. 2g,h). By contrast, a more negative *B*_flux_ indicates a relatively stable AMOC in the slow CO_2_ ramp and RCP2.6 simulations. Like the evolution of *B*_flux_, the buoyancy transfer^10^ from the surface to the deep ocean is also strongly timescale dependent, as captured by the bulk buoyancy frequency, which measures the degree of stratification in the given depth range (Extended Data Fig. 4). In the faster CO_2_ ramps, the stratification in the upper 1,000 m increases much more strongly in absolute values and relative to the deep ocean (which also becomes more stratified), compared with the slow CO_2_ ramp.

**Extended Data Fig. 3:**
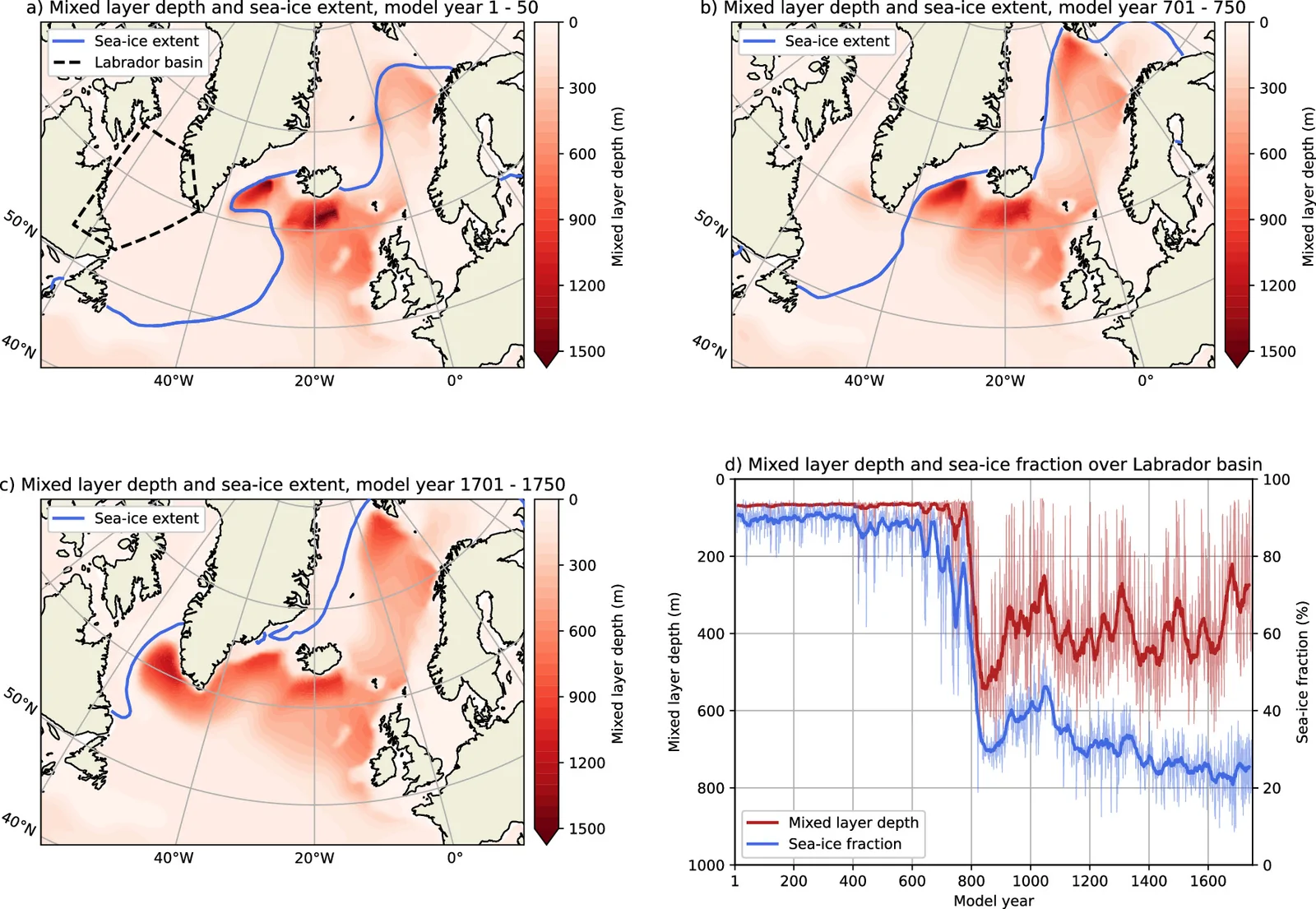
Mixed layer depth and sea-ice extent for the slow CO_2_ ramp. (**a**): The March mixed layer depth and sea-ice extent (that is, 15% sea-ice fraction contour) for model years 1 – 50. (**b** & **c**): Similar to panel a, but now for **b**) model years 701 – 750 and **c**) 1701 – 1750. (**d**): The spatially-averaged March mixed layer depth and sea-ice fraction over the Labrador basin (black outlined region in panel **a**). The thin curves are yearly averages, whereas the thick curves are smoothed versions (25-year moving averages). Basemaps in **a**–**c** generated with Cartopy^84^.

In the slow CO_2_ ramp, the AMOC strength undergoes a relatively rapid increase by about 3 Sv around model year 800 (Fig. 1c), which is related to the retreating sea-ice extent over the North Atlantic Ocean. Upon initialization from ${{\rm{PI}}}_{45}^{{\rm{on}}}$, the sea ice extends relatively far south (Extended Data Fig. 3a), owing to a weak AMOC strength of about 13 Sv under the background hosing of $\overline{{F}_{{\rm{H}}}}=0.45\,\mathrm{Sv}$. Sea ice limits ocean–atmosphere exchange and deep convection^65^; hence, the mixed layer depth is relatively shallow (<100 m) where sea ice is present. Importantly, there is initially no deep convection over the Labrador Sea due to the extensive sea-ice extent. When sea ice retreats poleward under higher temperatures, the Labrador Sea becomes less sea-ice covered, and this allows for a deeper mixed layer (Extended Data Fig. 3b–d). Vertical mixing brings relatively warm subsurface water masses to the surface, strongly contributing to the retreating sea-ice extent over the Labrador Sea, and giving rise to nonlinear responses under the linear CO_2_ ramp. Labrador Sea deep convection starts from model year 770 and onwards, followed by the 3-Sv increase in AMOC strength around model year 800. The role of deep convection in WMT rates is quite complex^69^, and this convection certainly plays an important role in modulating AMOC strength and collapsing the AMOC^35^. Hence, we expect that the 3-Sv increase can only be found when additional deep convection sites are activated as a result of retreating sea ice.

In summary, the analysis of the CO_2_ ramp simulations and comparison with the RCP scenarios (Fig. 3) show that the ocean response to radiative forcing changes depends qualitatively on the forcing rate. In the slow CO_2_ ramp, in which the atmopsheric warming rate is (much) smaller than in the faster CO_2_ ramps, RCP4.5 and RCP8.5, the AMOC remains stable up to +5.5 °C warming. In fact, from the *Ψ*_NADW_ and *B*_flux_ analysis, we conclude that the AMOC is getting more stable in warmer climates.

## Freshwater budget under climate change

**Fig. 4:**
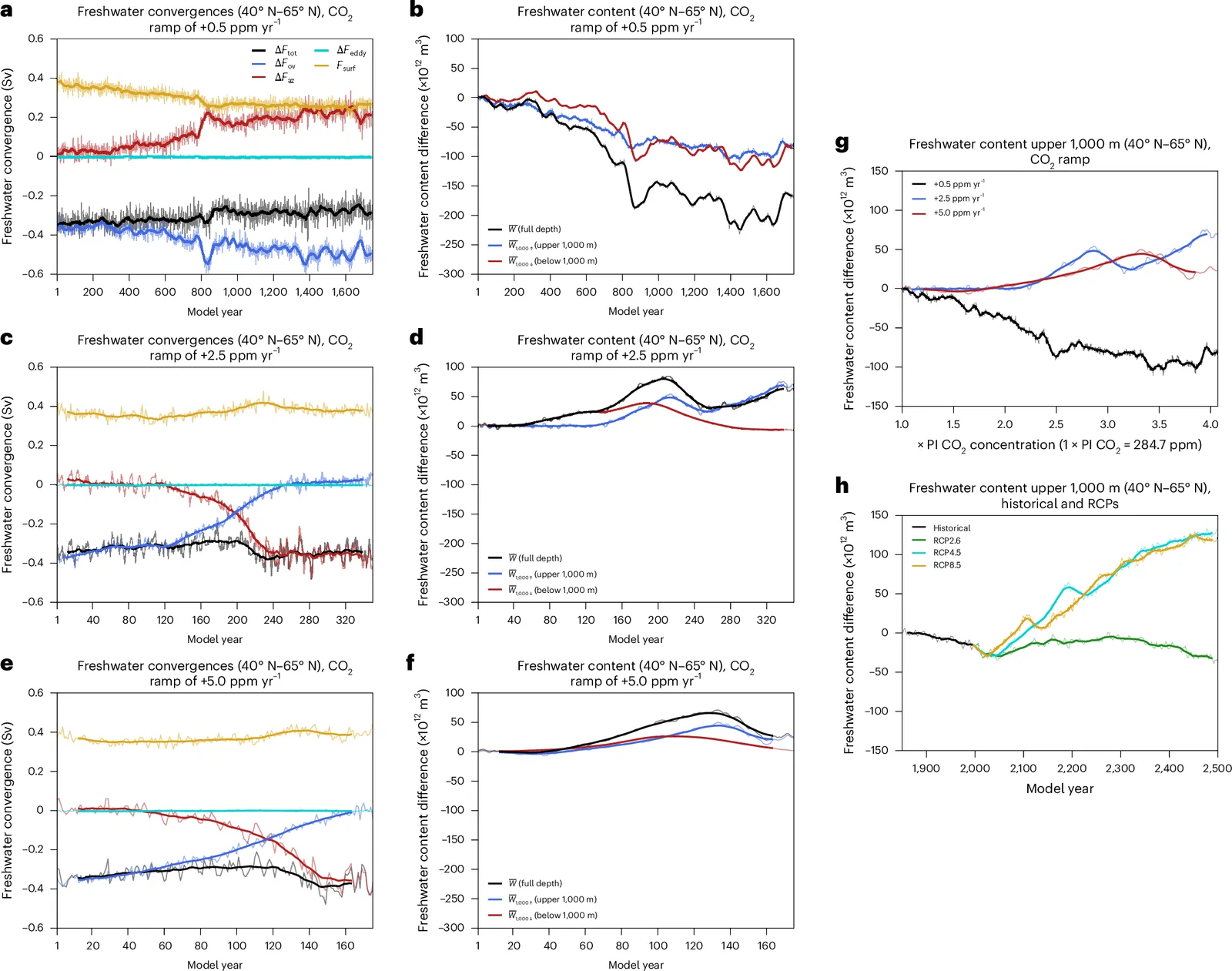
Freshwater convergence over the North Atlantic Ocean. **a**,**c**,**e**, The meridional freshwater convergences (40° N–65° N) for +0.5 ppm yr^−^^1^ (**a**), +2.5 ppm yr^−1^ (**b**) and +5.0 ppm yr^−1^ (**c**) CO_2_ ramps, including the surface freshwater flux (see legend in **a**). **b**,**d**,**f**, The freshwater content (40° N–65° N) difference (compared with ${{\rm{PI}}}_{45}^{{\rm{on}}}$) for the +0.5 ppm yr^−1^ (**b**), +2.5 ppm yr^−1^ (**d**) and +5.0 ppm yr^−1^ (**f**) CO_2_ ramps, which is split into an upper 1,000 m and below 1,000 m contribution. **g**,**h**, The upper 1,000 m freshwater content difference for the CO_2_ ramps (compared with ${{\rm{PI}}}_{45}^{{\rm{on}}}$, in units of PI CO_2_) (**g**) and historical and RCP scenarios (compared with the historical period of 1850–1899) (**h**). In all panels, the thin curves are yearly averages, whereas the thick curves are smoothed versions (25-year moving averages).

**Extended Data Fig. 4:**
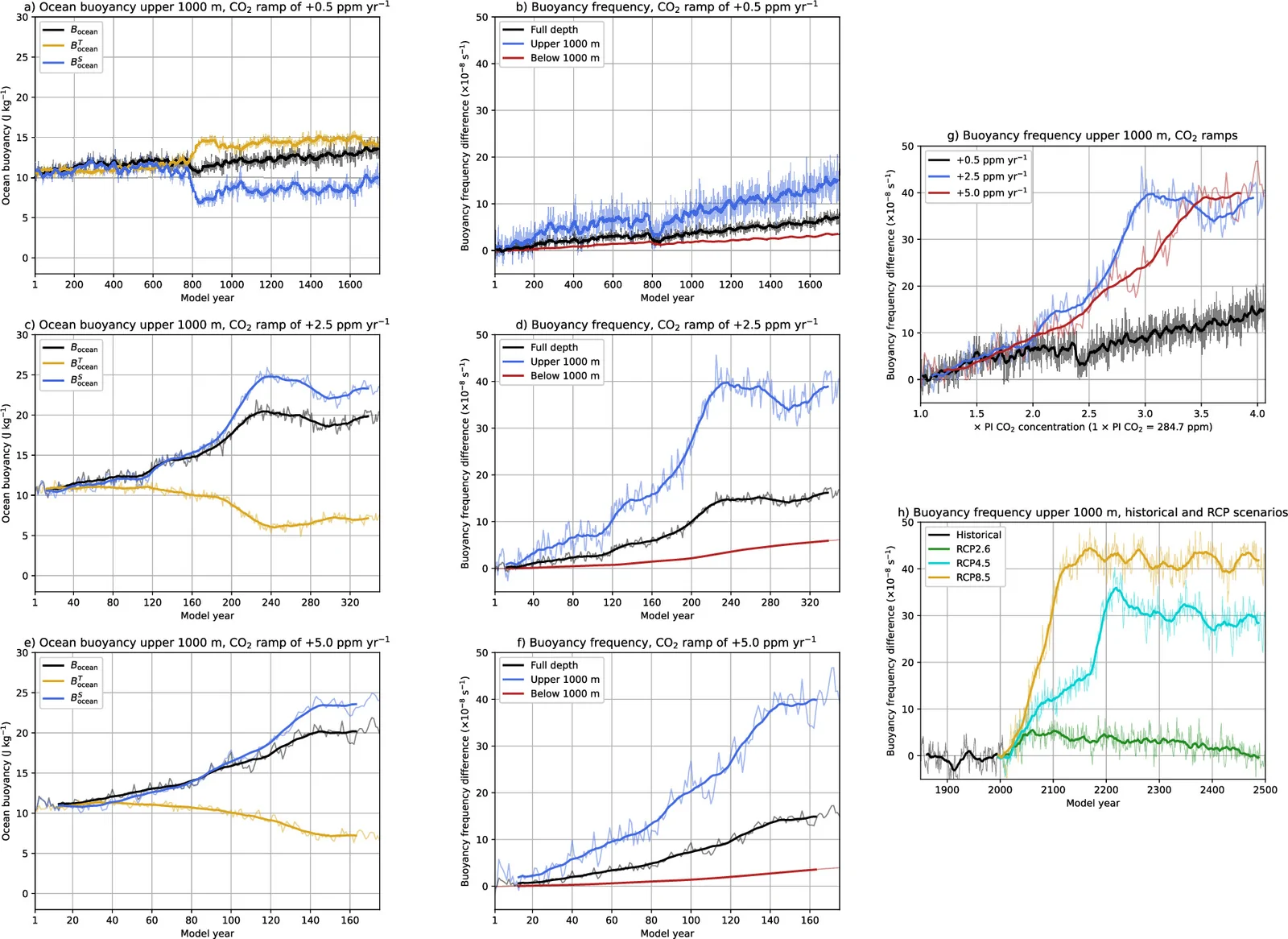
Upper 1000 m ocean buoyancy. (**a**,**c**,**e**): The upper 1,000 m ocean buoyancy between 40°N – 65°N for the three CO_2_ ramps. The ocean buoyancy is decomposed into a temperature and salinity contribution using the fixed salinity and temperature fields from ${{\rm{PI}}}_{45}^{{\rm{on}}}$, respectively. (**b**,**d**,**f**): The buoyancy frequency (${N}^{2}=\frac{-g}{{\rho }_{0}}\frac{\partial \rho }{\partial z}$) difference (compared to ${{\rm{PI}}}_{45}^{{\rm{on}}}$) for the three CO_2_ ramps, which is split into an upper 1,000 m and below 1,000 m contribution. (**g**,**h**): The upper 1,000 m buoyancy frequency difference for the CO_2_ ramps (compared to ${{\rm{PI}}}_{45}^{{\rm{on}}}$, in units of PI CO_2_) and historical and RCP scenarios (compared to the historical period of 1850 – 1899). In all panels, the thin curves are yearly averages, whereas the thick curves are smoothed versions (25-year moving averages).

Ultimately, an AMOC collapse is caused by net freshwater accumulation over the northern North Atlantic and is driven by the destabilizing salt-advection feedback^24–26^. This feedback can be activated by noise, freshwater perturbations or transient climate change^29,31,33,35^. There is also a heat-advection feedback that tends to stabilize the AMOC^27^, but its effect is smaller than that of freshwater accumulation when expressed in terms of ocean buoyancy^34^ (Extended Data Fig. 4a,c,e). Hence, we focus on the freshwater convergence between 40° N and 65° N (Fig. 4; Methods).

For the slow CO_2_ ramp, the total freshwater convergence Δ*F*_tot_ remains fairly constant over the first 800 model years and thereafter slightly increases (Fig. 4a). The total convergence is decomposed into its contributing factors, indicating that the overturning convergence is declining (Δ*F*_ov_, that is, salinifying) while being balanced by the azonal (gyre) convergence (Δ*F*_az_, that is, freshening). The declining Δ*F*_ov_ values are consistent with the relatively large salinity increase south of 40° N (Extended Data Fig. 1f), such that the AMOC imports net salinity into the 40° N–65° N latitude band. However, these responses of Δ*F*_ov_ and Δ*F*_az_ cannot explain the lower freshwater content ($\overline{W}$, that is, salinifying; Fig. 4b) when considering the constant Δ*F*_tot_ over the first 800 model years. These declining values of $\overline{W}$ are primarily driven by a smaller freshwater input through the surface (*F*_surf_), which was extensively discussed above (Extended Data Fig. 2).

The freshwater budget over 40° N–65° N evolves completely differently for the faster CO_2_ ramps (Fig. 4c–f). The Δ*F*_ov_ is strongly increasing and, together with net freshwater accumulation over the upper 1,000 m, is indicative of the dominant and destabilizing salt-advection feedback that causes the AMOC to collapse. Similar to the slow CO_2_ ramp, Δ*F*_az_ has the opposite response to Δ*F*_ov_, but now Δ*F*_az_ is removing the freshwater anomalies from the latitude band. The salt-advection feedback is triggered once the AMOC becomes sufficiently weak, with the initial AMOC weakening driven by an increase in the thermal component ${B}_{{\rm{flux}}}^{T}$, while ${B}_{{\rm{flux}}}^{S}$ exhibits the opposite response (Extended Data Fig. 2g). In other words, the surface freshwater fluxes do not generate a convergence of freshwater anomalies, as reflected by the slight decline in *F*_surf_ before the strong increase in Δ*F*_ov_. The freshwater accumulation over the upper 1,000 m (${\overline{W}}_{1000\uparrow }$) in the faster CO_2_ ramps has the opposite sign when comparing with the slow CO_2_ ramp (Fig. 4g). This dichotomy is also evident among the RCP scenarios (Fig. 4h). Lower values of ${\overline{W}}_{1000\uparrow }$ are therefore indicative of increased AMOC stability^70^, but should be considered with care as they can be compensated by temperature responses (Extended Data Fig. 4a).

Increasing values of *Ψ*_NADW_, together with decreasing values of *B*_flux_ and ${\overline{W}}_{1000\uparrow }$, suggest that the AMOC becomes more stable during the slow CO_2_ ramp. The strength of the salt-advection feedback, which relates to AMOC instability, is quantified by the AMOC-induced freshwater transport at 34° S, that is, *F*_ovS_. The use of *F*_ovS_ as a stability indicator is derived from the entire Atlantic freshwater budget (34° S to 65° N) and is appropriate only under (quasi-)equilibrium conditions^25,27,71^. This assumption certainly does not apply for the faster CO_2_ ramps (Fig. 1d, inset) nor the RCP4.5 and RCP8.5 scenarios^34^. The global warming rate in the slow CO_2_ ramp is on average 0.03 °C per decade, about a factor of 10 slower compared with that in the CO_2_ ramp of +5.0 ppm yr^−1^, the RCP8.5 scenario over the twenty-first century, and the currently observed warming rate^72,73^.

**Fig. 5:**
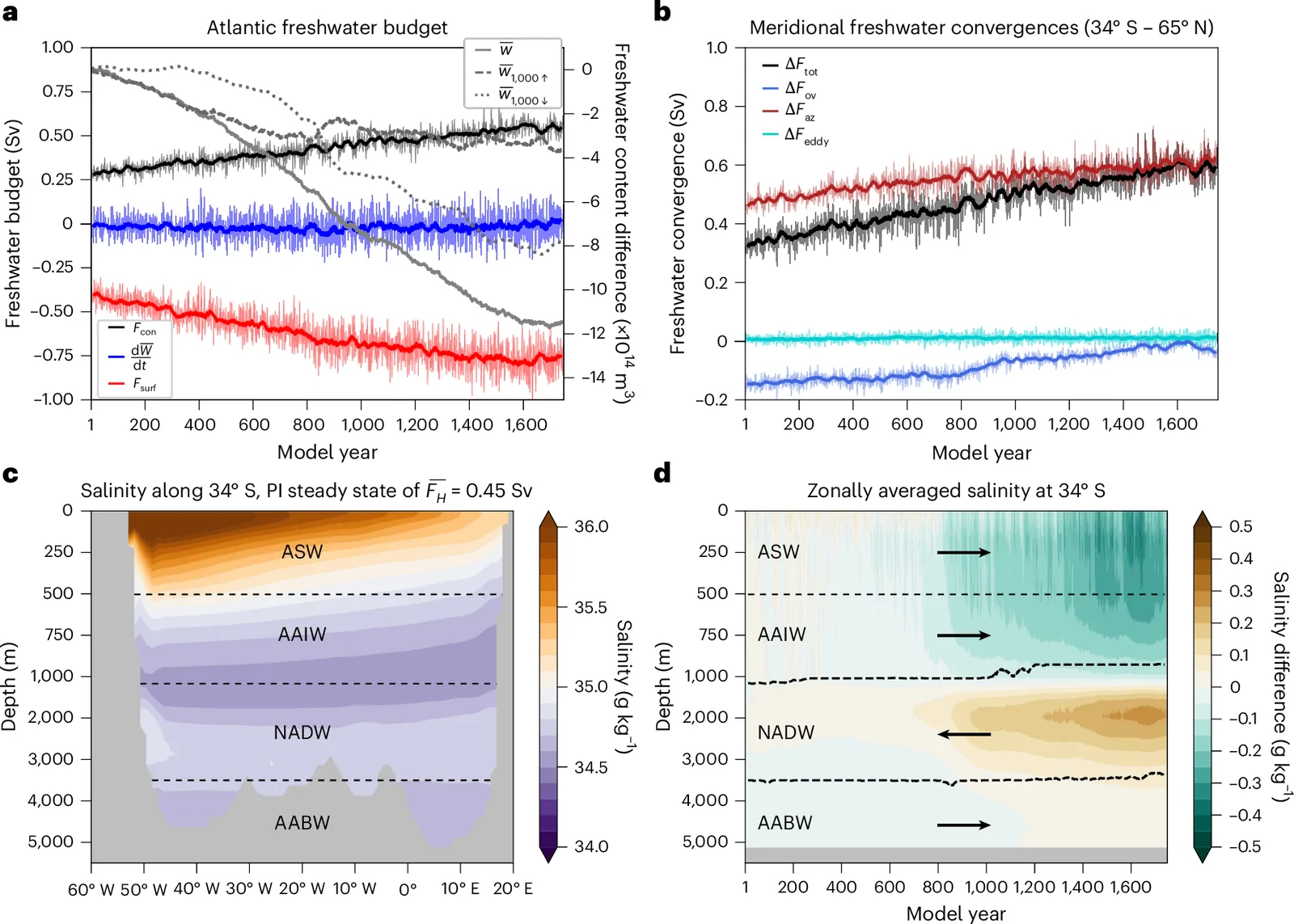
The Atlantic freshwater budget for the slow CO_2_ ramp. **a**, The Atlantic Ocean (34° S to 65° N) freshwater budget with the freshwater content ($\overline{W}$), freshwater convergence (*F*_con_), surface freshwater fluxes (*F*_surf_) and changes in the freshwater content ($\frac{{\rm{d}}\overline{W}}{{\rm{d}}t}$). The quantity $\overline{W}$ is split into an upper 1,000 m contribution (${\overline{W}}_{1000\uparrow }$) and below 1,000 m contribution (${\overline{W}}_{1000\downarrow }$) and is displayed as their differences compared with ${{\rm{PI}}}_{45}^{{\rm{on}}}$. **b**, The meridional freshwater convergences (omitting the contribution by the Strait of Gibraltar) for the different freshwater transport components. **c**, The salinity along 34° S for ${{\rm{PI}}}_{45}^{{\rm{on}}}$, where the dashed lines indicate the different water masses that are based on the meridional velocity profile (see procedure outlined in ref. ^49^). **d**, The zonally averaged salinity at 34° S over time, which are displayed as differences compared with ${{\rm{PI}}}_{45}^{{\rm{on}}}$. The dashed lines are the different water masses. The arrows are indicative of the meridional velocity (right, northward; left, southward) associated with the AMOC. ASW, Atlantic surface water; AAIW, Antarctic intermediate water; NADW, North Atlantic deep water; AABW, Antarctic bottom water.

To further explore whether AMOC stability increases in the slow CO_2_ ramp simulation, the Atlantic freshwater budget is shown in Fig. 5a. The Atlantic freshwater content is decreasing (that is, salinifying) in warmer climates, which is driven by the surface freshwater fluxes and is partially being opposed by the horizontal freshwater convergence.

**Extended Data Fig. 5:**
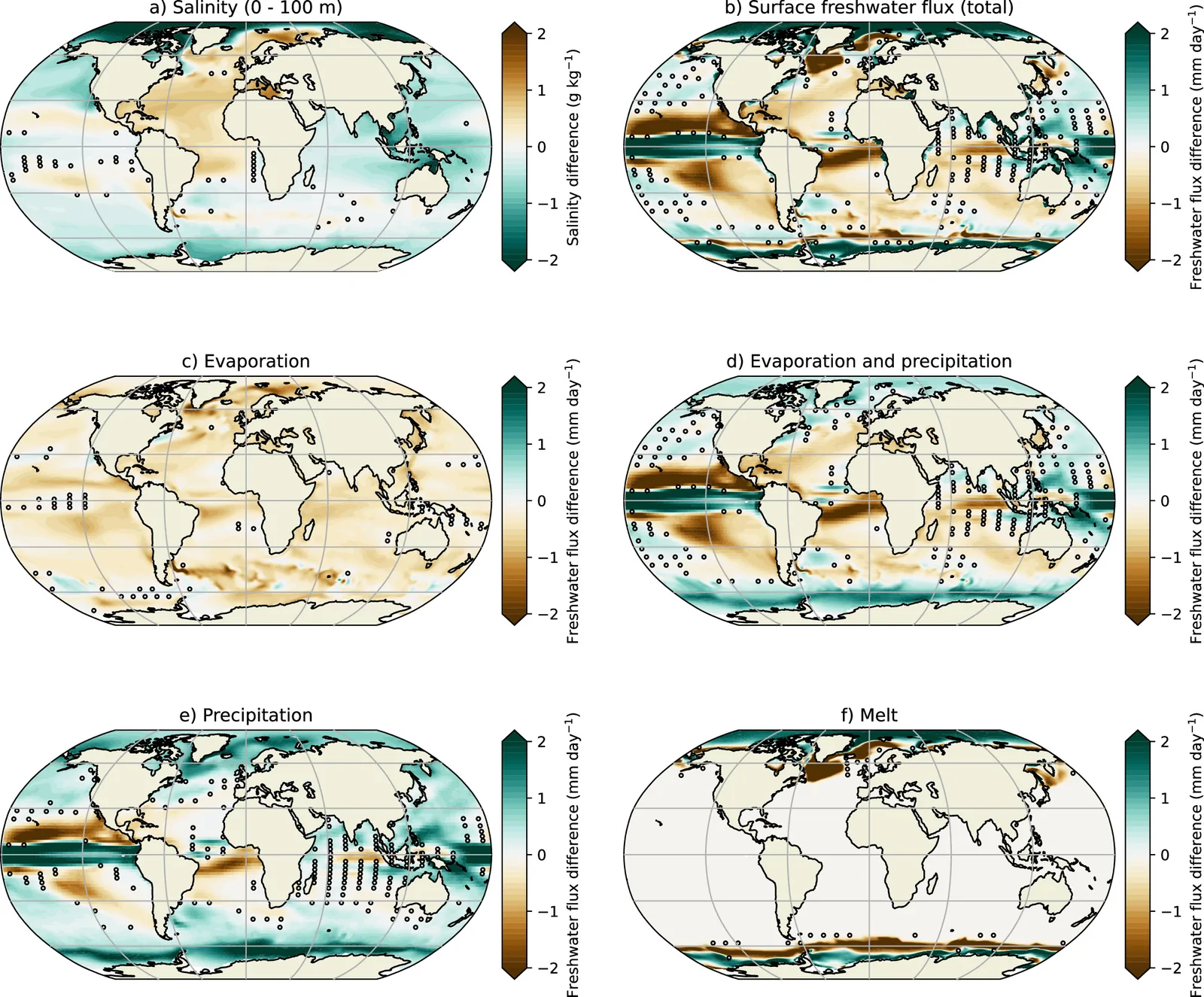
Salinity and freshwater flux responses for the slow CO_2_ ramp. (**a**): The depth-averaged (upper 100 m) salinity differences between model years 1701 – 1750 and ${{\rm{PI}}}_{45}^{{\rm{on}}}$. (**b** – **f**): The surface freshwater flux differences and its decomposition between model years 1701 – 1750 and ${{\rm{PI}}}_{45}^{{\rm{on}}}$ (positive = relative surface freshening). In all panels, the circled markers indicate non-significant (*p*≥0.05, two-sided Welch t-test) differences, markers were not displayed when no sea ice was present (panel **f**). Basemaps generated with Cartopy^84^.

We first analyse the surface-driven contribution, as the conventional thought is that global warming increases precipitation minus evaporation (*P* − *E*) over the North Atlantic. While precipitation increases over most of the ocean, indicative of an enhanced hydrological cycle, evaporation likewise increases over all ocean surfaces (Extended Data Fig. 5a–e). Over the Atlantic basin, this leads to a net negative *P* − *E* anomaly, with a portion of these freshwater anomalies is transferred via atmospheric bridges to other basins. Note that there is also a considerable sea-ice contribution at the higher latitudes (Extended Data Fig. 5f). The total surface freshwater flux response is opposite compared with that of the PI hosing simulation^33^, meaning that the (slow) climate change forcing is effectively a reversed hosing experiment (in terms of freshwater forcing).

**Extended Data Fig. 6:**
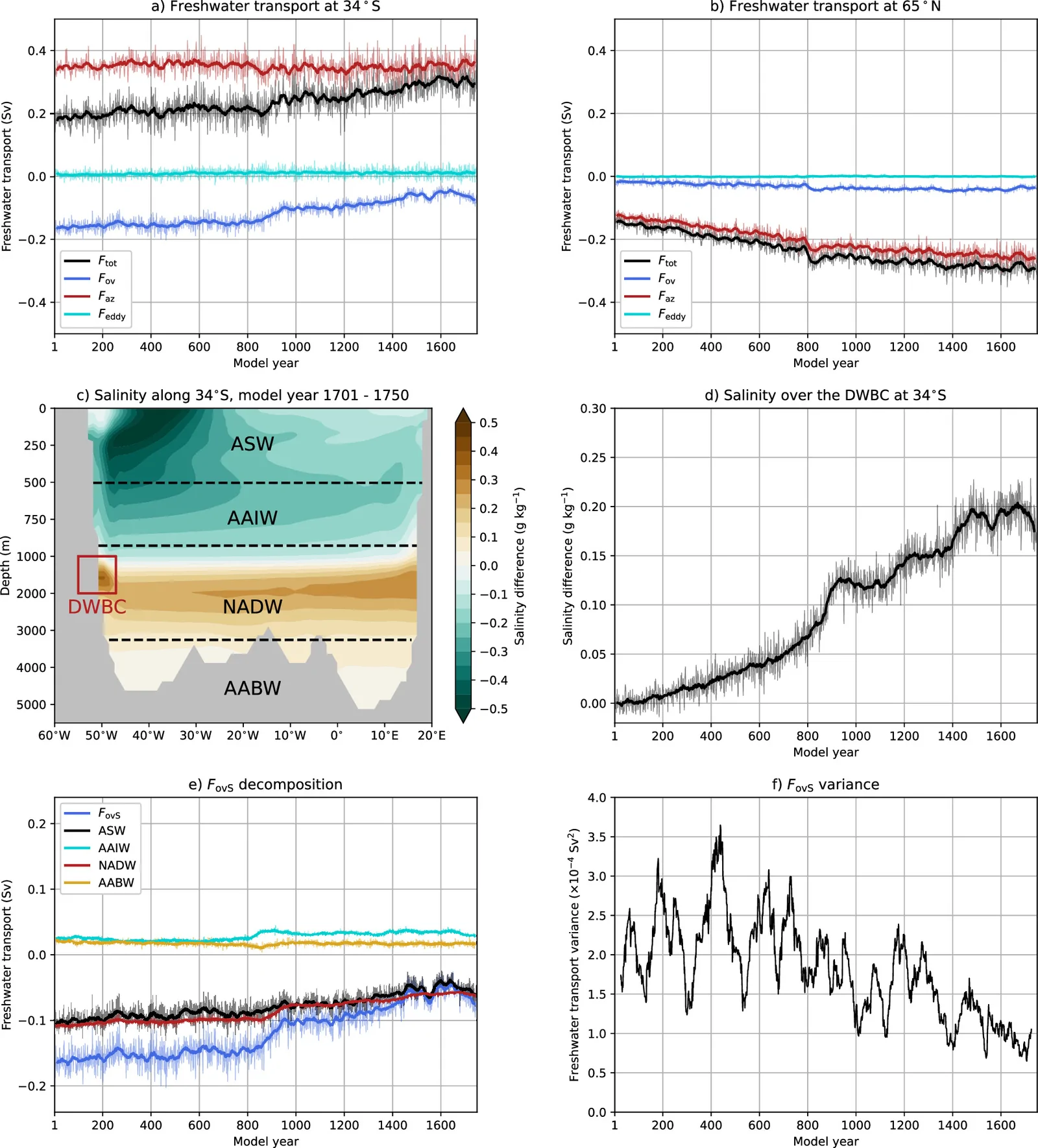
Freshwater transport decomposition for the slow CO_2_ ramp. (**a** & **b**): The freshwater transports at 34°S and 65°N. (**c**): The salinity difference along 34°S at the end of the simulation compared to ${{\rm{PI}}}_{45}^{{\rm{on}}}$, with the dashed lines indicating the different water masses^49^. (**d**): The salinity difference (compared to ${{\rm{PI}}}_{45}^{{\rm{on}}}$) over the deep western boundary current (DWBC, red outlined region in panel **c**). (**e**): The *F*_ovS_ decomposition, following the procedure outlined in^49^. (**f**): The *F*_ovS_ variance, where the variance is determined over 50-year sliding windows. A linear trend is removed over the 50-year window before determining the variance. In panels **a** – **e**, the thin curves are yearly averages, whereas the thick curves are smoothed versions (25-year moving averages).

Next, we analyse the Atlantic freshwater convergence, which is again decomposed into its dominant contributing factors (Fig. 5b). While Δ*F*_az_ is mostly contributing before model year 800, Δ*F*_ov_ thereafter also contributes. The increase in Δ*F*_az_ is primarily caused by a declining gyre freshwater transport at 65° N over the first 800 model years, after which Δ*F*_az_ stabilizes (Extended Data Fig. 6b). This response appears to be related to the retreating North Atlantic sea-ice extent. The freshwater transport by the overturning component at 34° S, *F*_ovS_, starts to contribute after model year 800 (Extended Data Fig. 6a), which is also reflected by the salinity responses at 34° S (Fig. 5c,d).

The salinifying NADW means that the AMOC is exporting salinity anomalies out of the basin, resulting in a larger *F*_ovS_ and indicative of increased AMOC stability. These salinity anomalies are formed in the northern North Atlantic Ocean (Fig. 4b) and a major pathway for these anomalies to leave the Atlantic basin is via the deep western boundary current (DWBC). The salinity over the DWBC at 34° S increases monotonically (Extended Data Fig. 6c,d), but note that the salinity across the entire zonal extent takes longer to adjust, explaining why these salinity anomalies become more pronounced after model year 700 (Fig. 5d). The resulting effect is that *F*_ovS_ is increasing (Extended Data Fig. 6e), meaning that the salt-advection feedback becomes weaker^27^. Increased *F*_ovS_ variance is also indicative of a stronger salt-advection feedback and, hence, a less stable AMOC^33,74^. In the slow CO_2_ ramp, the *F*_ovS_ variance declines (Extended Data Fig. 6f), which also suggests increased AMOC stability.

**Extended Data Fig. 7:**
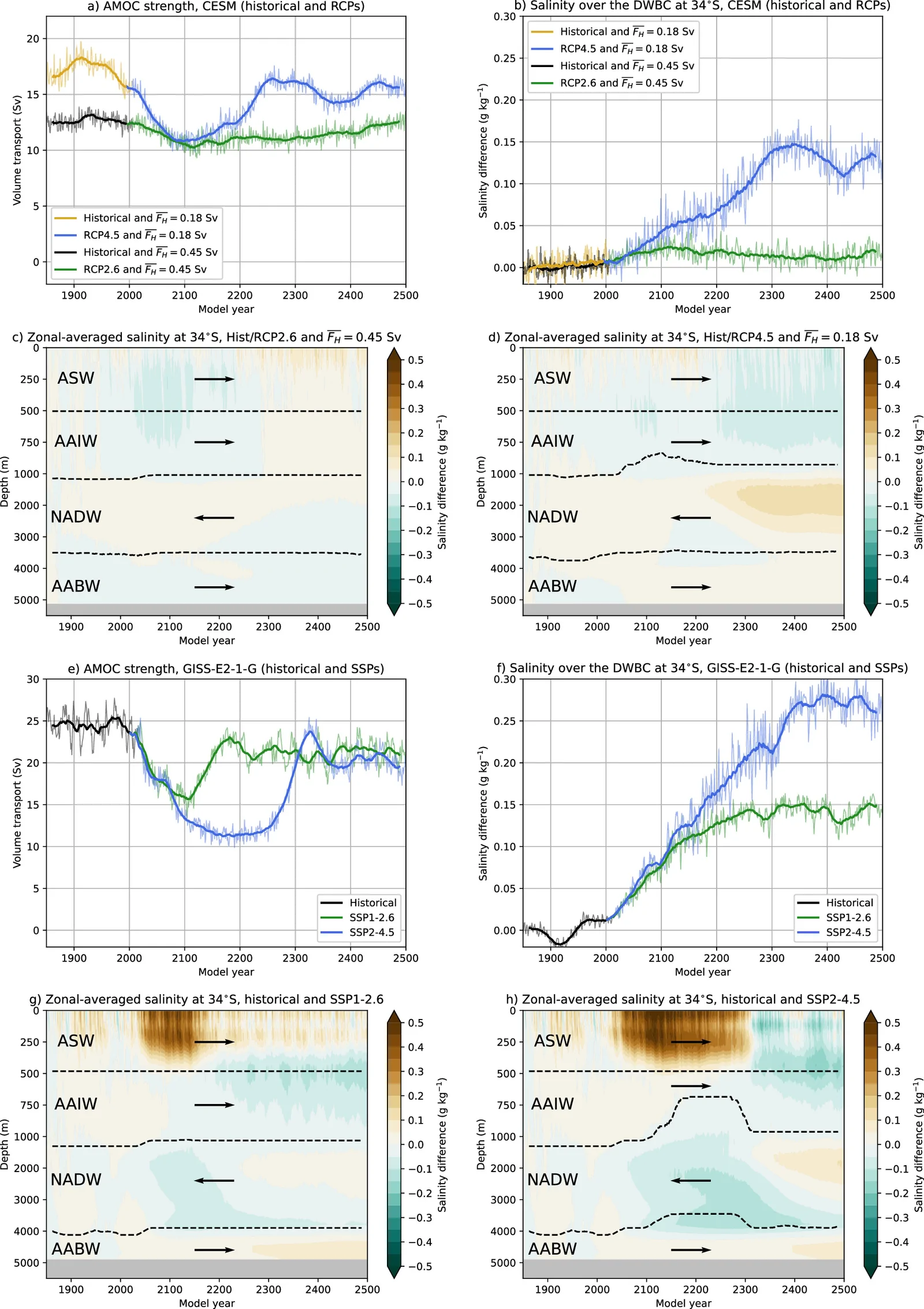
AMOC strength and salinity at 34°S under climate change. (**a**): The AMOC strength (at 26°N and 1,000 m depth) for the historical and RCP2.6 with $\overline{{F}_{H}}=0.45$ Sv and historical and RCP4.5 with $\overline{{F}_{H}}=0.18$ Sv. (**b**): Similar to panel a, but now for the salinity over the DWBC region (region in Extended Data Figures 6c) compared to 1850 – 1899. (**c** & **d**): Similar to Fig. 5d, but now for the two climate change scenarios. (**e** – **h**): Similar to panels **a** – **d**, but now for the GISS-E2-1-G (r2i1p1f2). In panels **a**,**b**,**e**,**f**, the thin curves are yearly averages, whereas the thick curves are smoothed versions (25-year moving averages).

The salinifying NADW is therefore an important fingerprint for increased AMOC stability, which is being explored in CESM under the RCPs and GISS-E2-1-G under the SSPs (that do not exhibit AMOC collapses; Extended Data Fig. 7). The DWBC is indeed salinifying in all climate change scenarios. However, the salinifying NADW is found only under the higher-emission scenario, which is related to adjustment timescales and/or the GMST anomaly. For the latter, a higher GMST anomaly results in a saltier NADW and DWBC, which was explicitly demonstrated for the slow CO_2_ ramp.

**Extended Data Fig. 8:**
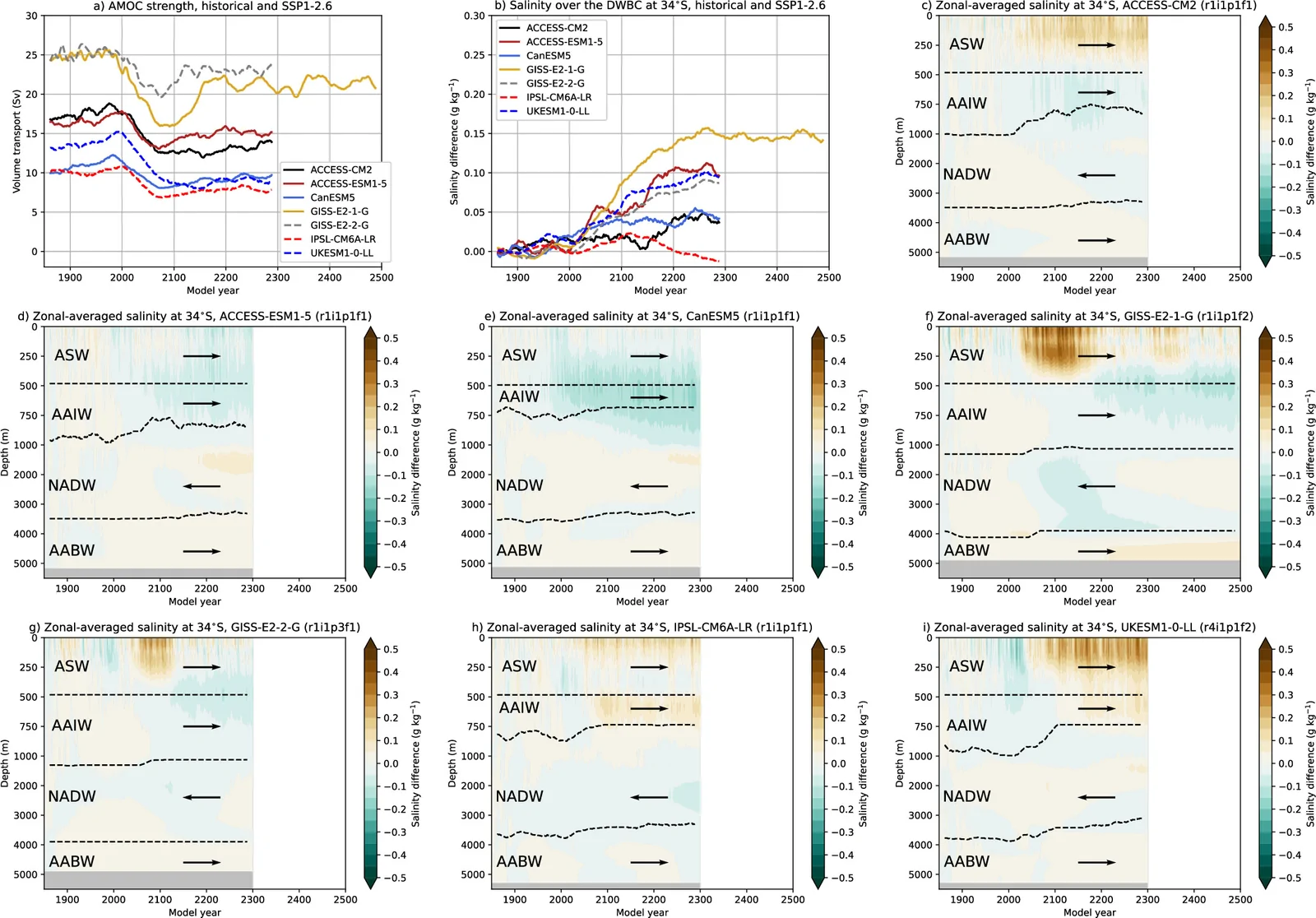
AMOC strength and salinity at 34°S under the historical and SSP1-2.6 scenario. Similar to Extended Data Figure 7, but now for 7 different CMIP6 models under the historical and SSP1-2.6. The specific realisations are indicated in the captions of panels **c** through **i**.

We also test whether a salinification of the NADW occurs in the remaining CMIP6 models under the extended SSP1-2.6. Most CMIP6 models show an increase in the DWBC salinities, but again the NADW responses are less pronounced (Extended Data Fig. 8). The IPSL-CM6A-LR is the only model where the DWBC and NADW become fresher by 2300. Most CMIP6 models align qualitatively with our CESM results, suggesting increased stability of the strong AMOC steady state under warmer climates. Note that the near-surface freshening at 34° S (Fig. 5d) is also contributing to increasing *F*_ovS_ values in the slow CO_2_ ramp (Extended Data Fig. 6e), but there is a large intermodel spread, probably related to differences in ocean–atmosphere interactions, Agulhas leakage and model resolution used^49,75^.

Sufficiently slow climate change allows coherent oceanic adjustment (Fig. 3), thereby preventing an AMOC collapse in the first place. In addition, changes in the Atlantic freshwater budget reveal that the AMOC becomes more stable in warmer climates. Importantly, this AMOC stabilizing mechanism adjusts over centennial timescales, which is set by the propagation of NADW salinity anomalies out of the Atlantic Ocean. The different timescales on which climate change induces AMOC weakening (years to decades) and AMOC stabilization (centuries) explain why the AMOC may collapse under climate change. Consequently, we conclude that the AMOC collapse observed under faster CO_2_ ramps and higher RCP scenarios is rate-induced: it occurs because the warming rate exceeds a critical threshold, without the global warming level crossing a critical threshold.

## Dynamical perspective

**Extended Data Fig. 9:**
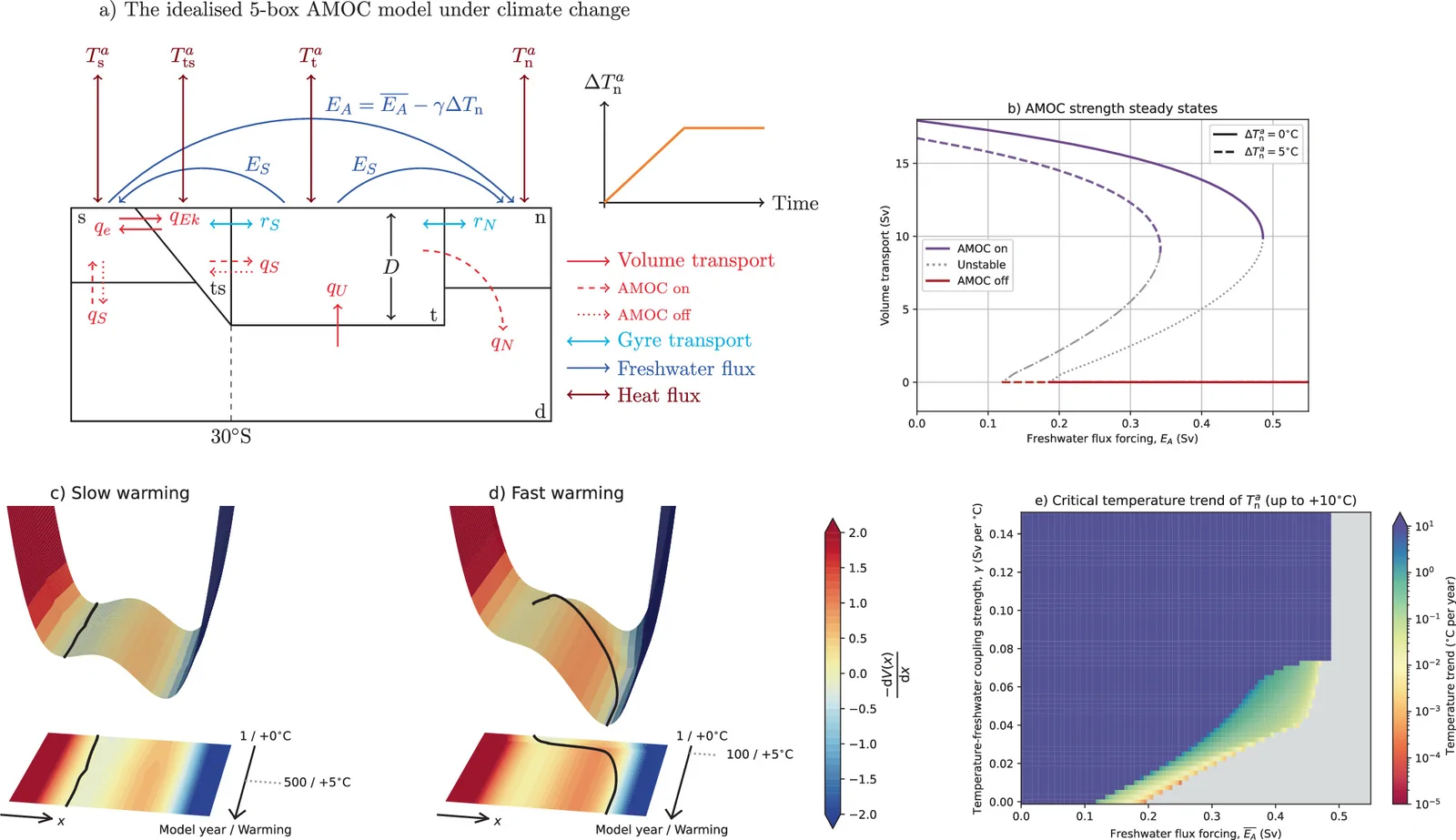
The 5-box AMOC model. (**a**): Schematic of the 5-box AMOC model, with an AMOC on state (clockwise circulation, dashed red arrows) and AMOC off state (anticlockwise circulation, dotted red arrows). (**b**): Bifurcation diagram under varying *E*_*A*_, showing equilibria for fixed $\Delta {T}_{{\rm{n}}}^{a}={0}^{\circ }$C and $\Delta {T}_{{\rm{n}}}^{a}={5}^{\circ }$C. (**c** & **d**): Sketch of the (forcing path-dependent) stability landscape as a classical double-well potential, with the left and right minima representing the ‘AMOC on’ and ‘AMOC off’ states, respectively, shown for the slow and fast warming cases. In this landscape, the system state is remapped based on the distance to the saddle-node bifurcation, where a larger (smaller) distance means that the AMOC is more (less) stable, as is reflected by a relatively deep (shallow) left minimum. The system trajectory is shown by the black curve. (**e**): Similar to Fig. 6d, but now for a linear ramp up to $\Delta {T}_{{\rm{n}}}^{a}=1{0}^{\circ }$C.

It remains unclear whether the AMOC could cross an equilibrium bifurcation under anthropogenic climate change. Addressing this question requires additional quasi-equilibrium simulations in the forcing space of imposed freshwater fluxes and radiative forcing. Moreover, the AMOC response depends on the background climate state, for example on $\overline{{F}_{H}}$ (ref. ^34^), and sensitivity experiments with models like the CESM require substantial computational resources. Therefore, we here use a five-box ocean model of the Atlantic basin (Extended Data Fig. 9a; Methods) to conceptualize the forcing path dependence of AMOC tipping.

The box model has been shown to capture the essential AMOC dynamics of the CESM^7^. In our context, the two main forcing parameters are the freshwater flux forcing (*E*_A_) and the atmospheric temperature anomaly over the subpolar Atlantic box ($\Delta {T}_{{\rm{n}}}^{a}$). The effects of global warming are incorporated by increasing $\Delta {T}_{{\rm{n}}}^{a}$ to lower the meridional atmospheric temperature gradient between the lower and higher latitudes, mimicking polar amplification^7^. For each $\Delta {T}_{{\rm{n}}}^{a}$ increment of 0.1 °C, we calculate the steady states and bifurcations of the box model using continuation techniques, with *E*_A_ as the bifurcation parameter. The model features a bistable AMOC regime bounded by saddle-node bifurcations, which can be crossed in both forcing directions (*E*_A_ and $\Delta {T}_{{\rm{n}}}^{a}$). The saddle-node bifurcation shifts in freshwater forcing space from *E*_A_ = 0.486 Sv for $\Delta {T}_{{\rm{n}}}^{a}={0}^{\circ }$ to *E*_A_ = 0.342 Sv for $\Delta {T}_{{\rm{n}}}^{a}=+{5}^{\circ }$ (Extended Data Fig. 9b).

**Fig. 6:**
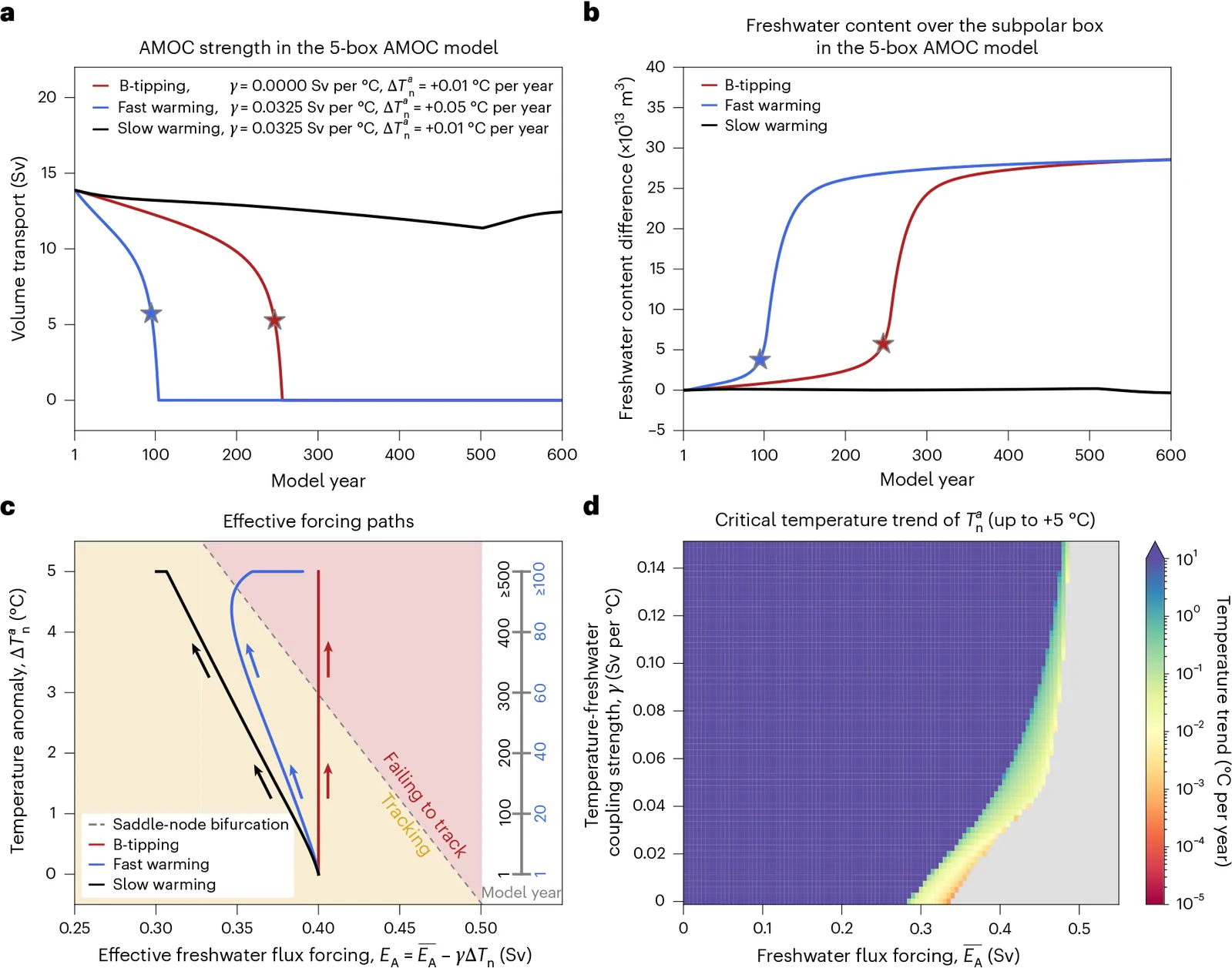
AMOC transitions in the five-box model. **a**,**b**, The AMOC strength (**a**) and the freshwater content difference (**b**, compared with the initial state) over the subpolar box for three different forcing paths (see legend in **a** for details); all forcing paths have $\overline{{E}_{{\rm{A}}}}=0.40\,\mathrm{Sv}$. Stars indicate the crossing of the shifting saddle-node bifurcation. **c**, Effective forcing paths in the forcing space of subpolar warming and effective freshwater flux. The position of the saddle-node bifurcation of the stable AMOC on state for increasing $\Delta {T}_{{\rm{n}}}^{a}$ divides the effective forcing space into two qualitative regimes (tracking/failing to track the AMOC on state). **d**, The critical temperature trend of ${T}_{{\rm{n}}}^{a}$ without collapsing the AMOC for varying $\overline{{E}_{A}}$ and temperature–freshwater coupling strength *γ*, the lowest explored trend is 1 × 10^−5^ °C per year. In the grey region, tracking the AMOC-on state fails for all trends considered.

First, consider cases with fixed *E*_A_ > 0.342 Sv. The AMOC always collapses under an imposed warming anomaly of $\Delta {T}_{{\rm{n}}}^{a}=+{5}^{\circ }$ or more, since the ‘AMOC on’ state disappears. Thus, a critical warming threshold exists beyond which the system transitions to the only remaining stable solution (the collapsed ‘AMOC off’ state), regardless of the rate of increase at which the warming anomaly is reached. This is a case of bifurcation-induced tipping (B-tipping). An example is given for a fixed freshwater flux forcing ($\overline{{E}_{A}}$) of 0.40 Sv, forced under a linear temperature trend of $\Delta {T}_{{\rm{n}}}^{a}=+0.0{1}^{\circ }$ per year up to +5 °C (Fig. 6a, red curve).

This situation is qualitatively inconsistent with the slow CO_2_ ramp in CESM, where the ‘AMOC on’ state remains stable up to at least +5.5 °C. However, the box model so far neglects the interdependence between surface heat and freshwater fluxes under radiative forcing change. To account for this in an idealized manner, we consider an effective freshwater flux forcing *E*_A_ that depends on the ocean state, specifically the temperature anomaly Δ*T*_n_ of the subpolar box: ${E}_{A}=\overline{{E}_{A}}-\gamma \Delta {T}_{{\rm{n}}}$. Here, $\overline{{E}_{A}}$ is the fixed freshwater flux forcing, *γ* is the temperature–freshwater coupling strength, and $\Delta {T}_{{\rm{n}}}={T}_{{\rm{n}}}(t)-{T}_{{\rm{n}}}(\overline{{E}_{A}},\Delta {T}_{{\rm{n}}}^{a}=0)$. This temperature–freshwater coupling is motivated by the declining North Atlantic surface freshwater input in the slow CO_2_ ramp simulation (Figs. 4a and 5a) where, for reference, the freshwater flux over 34° S–65° N declines by 0.069 Sv per degree warming (giving *γ* ≈ 0.069 Sv per degree Celsius).

We repeat the warming experiment (linear ramp up to $\Delta {T}_{{\rm{n}}}^{a}={5}^{\circ }{\rm{C}}$ at fixed $\overline{{E}_{{\rm{A}}}}=0.40\,\mathrm{Sv}$) but now with *γ* > 0, comparing relatively slow (+0.01 °C yr^−1^) and relatively fast (+0.05 °C yr^−1^) warming rates. For *γ* = 0.0325 Sv per degree Celsius, the system continues to track the ‘AMOC on’ state under the slow warming case (Fig. 6a, black curve). The equilibrated subpolar ocean warming Δ*T*_n_ is 3.04 °C, resulting in an effective hosing strength of 0.30 Sv, which is indeed below the bifurcation threshold of 0.342 Sv for $\Delta {T}_{{\rm{n}}}^{a}=+{5}^{\circ }{\rm{C}}$. However, in the fast warming case, the AMOC collapses as the system fails to track the ‘AMOC on’ state after crossing the moving saddle-node bifurcation (Fig. 6a, blue curve). Because the ‘AMOC on’ state remains stable for *γ* = 0.0325 Sv per degree Celsius (Extended Data Fig. 9c,d) and can be tracked in the slow warming case, the fast warming case represents a rate-induced AMOC tipping event.

To visualize the cases described above, we plot their effective forcing paths in a diagram of the warming anomaly against the effective freshwater flux forcing (Fig. 6c). For *γ* > 0, trajectories initially move towards lower effective freshwater flux forcing, and whether the saddle-node bifurcation is crossed depends on the forcing rate. Tracking the stable ‘AMOC on’ state hence depends on the ratios of atmospheric warming rate (forcing timescale) and ocean warming rate (adjustment timescale), as well as the interaction strength between thermal and freshwater effects. Due to the state-dependent surface forcing resulting from ocean–atmosphere coupling, the rate-dependent tipping scenario shown here is more complex than the classical setting of rate-induced tipping in which the control parameters are purely external^18,19^.

The box model is a strong simplification of the CESM dynamics and only mimicks one of the rate-dependent mechanisms identified in the CESM, namely the reduced freshwater flux through the Atlantic Ocean surface under gradual warming. Nonetheless, this process is sufficient to produce rate-dependent AMOC tipping in the box model, and the freshwater content in the subpolar box shows a qualitatively similar response to the freshwater content analysis in the CESM (Figs. 4g and 6b). The maximal temperature trend that does not cause an AMOC collapse (that is, the critical warming rate), is shown in Fig. 6d as a function of $\overline{{E}_{A}}$ and *γ*. For sufficiently large temperature–freshwater coupling strengths, the system can always track the stable ‘AMOC on’ state as long as it exists and the imposed forcing rate is slow enough. These results are qualitatively robust for even larger temperature anomalies of up to +10 °C (Extended Data Fig. 9e), demonstrating that, even for extreme warming, forcing pathways may exist for which the AMOC does not collapse.

## Discussion

In this study, we argue that an AMOC collapse is dependent on the radiative forcing path (that is, climate change scenario) and not governed by a specific global warming threshold. To demonstrate this, we presented an analysis of CO_2_ ramp simulations using the CESM, where the CO_2_ concentration was increased up to four times the PI levels at different rates. In the slow CO_2_ ramp (+0.5 ppm yr^−1^) simulation, the GMST anomaly reaches +5.5 °C and the AMOC remains stable throughout, while for the faster CO_2_ ramps (+2.5 ppm yr^−1^ and +5.0 ppm yr^−1^) the AMOC starts to collapse at +2.0 °C. This GMST anomaly is much lower than the maximum warming in the slow CO_2_ ramp, showing that an AMOC collapse is determined by the radiative forcing path and specifically the forcing rate. Comparable results are found under the standard climate change scenarios of RCP2.6 (stable AMOC) and RCP4.5 and RCP8.5 (collapsing AMOC), where we note that these RCPs have different forcing agent (CO_2_, methane and aerosols) pathways that all affect the AMOC^12,55,56^. Our result corroborates early work using idealized climate models^10^, while now being demonstrated with a much more comprehensive climate model.

Present-day and projected warming simulations using CMIP6 models show substantial AMOC weakening^38–40,76,77^ and possibly an AMOC collapse^29,35^. For the CESM under relatively rapid radiative forcing changes (≥+2.5 ppm yr^−1^ of CO_2_ and $\overline{{F}_{{\rm{H}}}}=0.45\,\mathrm{Sv}$), the near-surface water is getting lighter much faster than the interior of the Atlantic Ocean, resulting in a shutdown of the adiabatic pathways followed by an AMOC collapse. When the imposed radiative forcing is sufficiently slow, both the interior and surface waters adjust their densities accordingly such that they support a relatively strong adiabatic AMOC. With an idealized AMOC model subjected to heat and freshwater forcing, we illustrated how the interplay between the changing forcing and the changing climate state can alter the system’s stability landscape in a way that leads to rate-dependent AMOC tipping.

In the slow CO_2_ ramp, the relatively small temperature-induced AMOC weakening is outweighed by two dominant effects. First, higher evaporation rates make the evaporative characteristic of the Atlantic basin more pronounced, which effectively results in a negative freshwater flux forcing. Second, the North Atlantic sea-ice extent is reduced in a warmer climate, which limits the sea-ice insulation effects that influence the AMOC^30,65^ and also lowers the freshwater input by sea-ice melt, contributing to less freshwater input over the higher latitudes. Both net evaporation and reduced sea-ice extent contribute to a salinifying Atlantic Ocean and, after WMT, the AMOC starts to export these salinity anomalies out of the Atlantic basin at greater depth (1,000–3,500 m). This response at depth is indicative of a more stable AMOC (that is, larger *F*_ovS_ values), and a similar response is found in most CMIP6 models under extended SSP scenarios with a sustained AMOC. The freshwater budget analysis and related *F*_ovS_ stability indicator are only meaningful sufficiently close to equilibrium^25,27^ and are not useful under rapid climate change^34^.

The stabilizing mechanism identified here may also explain the strong AMOC states found in warmer climates^12,13,16,29^. Some models, such as the CESM version used here, are in a monostable regime under PI conditions without background hosing^42^, and this is related to persistent climate model biases^45,49,50^, which could also be the case under different radiative forcing conditions.

There are now first indications that the AMOC can collapse under anthropogenic climate change in the latest generation of climate models^29,35^. This implies that the stabilizing mechanism in warmer climates, as described above, operates on timescales slower than the current rate of radiative forcing. Hence, it may still be useful to explore warming levels for an AMOC collapse onset on relatively short timescales (years to decades), but this becomes less useful if warming rates slow down (as projected beyond the twenty-first century) and should be complemented with threshold estimates for other relevant state variables and forcing parameters. Based on our results, we propose to convert the AMOC tipping warming threshold to a warming rate threshold. Yet, this also poses challenges as the critical warming rate is dependent on the background climate state and the strength of the stabilizing mechanism. Due to slow climate processes and nonlinear forcing, the exact forcing pathway and its history matter. Therefore, it is more useful to analyse physics-based indicators, such as *Ψ*_NADW_ and *B*_flux_, that capture the effects of changing forcing conditions (both stabilizing and destabilizing). In particular, a *B*_flux_ sign change is marking the onset of an AMOC collapse also under transient climate change^34^.

According to the *B*_flux_ indicator, as determined in CMIP6 models, the AMOC starts to collapse around +2.5 °C warming at projected warming rates, which could be reached around the year 2060^34^. This is substantially lower than the previous estimate of +4 °C warming^2^ that lacks a time horizon. Observations show a present-day warming close to the +1.5 °C level (2023–2025, C3S/ECMWF and ref. ^73^), and, if the AMOC were to begin collapsing at +2.5 °C warming in 2060, this would imply a critical warming rate of +0.29 °C per decade. This rate aligns with projected warming rates of +0.27 to +0.36 °C per decade^72^ and, also considering the effects of random variability^29,78^, strongly suggests that rate-dependent effects are highly relevant for the fate of the AMOC. It is therefore critically important to reduce greenhouse gas emissions as quickly as possible to limit the risk of an AMOC collapse.

## Methods

### Climate Model Simulations

The CESM is a fully coupled climate model and the simulations here have a 1° horizontal resolution for the ocean/sea-ice components and a 2° horizontal resolution for the atmosphere/land components. The ocean component is the Parallel Ocean Program version 2 (POP2)^79^, the atmospheric component is the Community Atmosphere Model version 4 (CAM4)^80^ and the sea-ice component is the Community Ice Code version 4 (CICE4)^81^. The CESM is either forced under varying freshwater flux forcing and fixed PI radiative forcing conditions or under fixed freshwater flux forcing and varying radiative forcing conditions. For more details on the precise CESM set-up, we refer to previous work^30,33,34,42^ and the main text.

In addition to the CESM, model output from CMIP phase 6 (indicated here as CMIP6 models) were analysed. We retained the historical forcing (1850–2014) followed by the extended SSP1-2.6 scenario (2015–2300). Only for the GISS-E2-1-G model were the scenarios extended to 2500 and are also available for the extended SSP2-4.5 scenario. Note that the forcing scenarios are slightly different between the CESM simulations (the RCPs) and CMIP6 (the SSPs).

### Water mass transformation

We use the same procedure as described in ref. ^34^, which is briefly repeated here for completeness. We consider the volume (*V*_*σ*_) conservation of a fixed horizontal domain that is bounded by the ocean surface and isopycnal^64^:

1 $$\frac{\partial {V}_{\sigma }}{\partial t}={M}_{\sigma }-{G}_{\sigma },$$

where *M*_*σ*_ is the advective transport convergence through the open boundaries of the domain and *G*_*σ*_ is the diapycnal transformation rate taken place in the domain. The WMT rates from the surface are dominant in *G*_*σ*_ and are indicated by *Ψ*_WMT_:

2 $${\varPsi }_{\mathrm{WMT}}(\,y,{\sigma }_{2})=\frac{1}{{\Delta }{\sigma }_{2}}{\int }_{{x}_{W}}^{{x}_{E}}{\int }_{y}^{{y}_{N}}-\frac{{\rho }_{0}}{g}{B}_{\mathrm{flux}}(x,y)\,\varPi ({\sigma }_{2})\,{\rm{d}}y^{\prime} {\rm{d}}x^{\prime}$$

where

3 $$\Pi ({\sigma }_{2})=\{\begin{array}{c}1\,\,\mathrm{if}\,{\sigma }_{2}-\frac{{\rm\Delta }{\sigma }_{2}}{2}\le {\sigma }_{2} < {\sigma }_{2}+\frac{{\rm\Delta }{\sigma }_{2}}{2}\\ 0\,\,\mathrm{elsewhere}\end{array},$$

and we use a potential density bin size of Δ*σ*_2_ = 0.05 kg m^−3^. The quantity *B*_flux_ is the surface buoyancy flux:

4 $${B}_{{\rm{flux}}}(x,y)=\frac{g\alpha }{{\rho }_{0}{C}_{p}}{Q}_{{\rm{heat}}}+\frac{g\beta {S}_{{\rm{surf}}}}{{\rho }_{0}}{Q}_{{\rm{fresh}}}={B}_{{\rm{flux}}}^{T}+{B}_{{\rm{flux}}}^{S}$$

and we also analyse the upper 1,000 m ocean buoyancy:

5 $${B}_{{\rm{ocean}}}(T,S)=\frac{g}{{\rho }_{0}}\int_{-\mathrm{1,000}}^{0}\left(\sigma (z)-\sigma (z=0)\right){\rm{d}}z.$$

In the relations above, *α* is the thermal expansion coefficient, *C*_*p*_ the specific heat capacity, *Q*_heat_ the net heat flux into the ocean, *β* the haline contraction coefficient, *S*_surf_ the sea surface salinity, *Q*_fresh_ the net freshwater flux into the ocean, *g* (9.8 m s^−2^) the gravitational acceleration, *z* the coordinate of integration and *ρ*_0_ (1,027 kg m^−3^) a reference density. For each quantity, we used its local value, and for *α*, *β* and *C*_*p*_ we used the Thermodynamic Equation of SeaWater 2010 (TEOS-10) toolkit^82^. All the analyses are conducted on monthly averaged fields (due to strong seasonal cycle), and the related time series are subsequently averaged to yearly values.

### Defining the onset of AMOC collapse

A freshwater forcing threshold for AMOC collapse can be accurately determined in the quasi-equilibrium PI hosing simulation, where the crossing of the tipping point in the forcing coincides in time with the start of abrupt AMOC weakening^33^. However, under transient climate change, it is more challenging to deduce the onset of the collapse, as the transition is not sharp in time and is preceded by AMOC weakening^35^. Defining the onset of the collapse based on AMOC strength has its limitations^7,32,34^, and instead, van Westen et al. ^34^ proposed the sign change in the spatially averaged *B*_flux_ over the North Atlantic isopycnal outcropping region (40° N–65° N; Extended Data Fig. 2g,h). The timing of the sign change is robust when this latitudinal band is slightly varied. The spatially averaged *B*_flux_ is strongly related to the adiabatic pathways that are part of the AMOC (that is, *Ψ*_NADW_; see main text), and this estimate works very well for CESM, as well as for other CMIP6 models^34^.

Here, the AMOC collapse onset is defined as the timepoint (or corresponding GMST anomaly) at which the 11-year running mean of *B*_flux_ first switches sign from negative to positive (Extended Data Table 1). This definition holds whether the tipping event is rate-induced or bifurcation-induced.

### The Atlantic freshwater budget

The freshwater budget over a latitude band (*y*_1_ to *y*_2_) in the Atlantic Ocean is defined as

6a $$\frac{{\rm{d}}\overline{W}}{{\rm{d}}t}={F}_{{\rm{con}}}+{F}_{{\rm{surf}}}+{F}_{{\rm{mix}}}$$

6b $$\overline{W}=-\frac{1}{{S}_{0}}\int_{-H}^{0}\int_{{y}_{1}}^{{y}_{2}}\int_{{x}_{W}}^{{x}_{E}}(S-{S}_{0}){\rm{d}}x{\rm{d}}y{\rm{d}}z$$

where $\overline{W}$ is the freshwater content, *F*_con_ is the freshwater convergence and is determined as the total freshwater transport through the boundaries, *F*_surf_ is the surface freshwater flux and *F*_mix_ is a residual term that closes the budget and captures for example diffusion^83^. *F*_con_ is primarily governed by the total meridional freshwater transports and is defined by

7 $${F}_{{\rm{tot}}}(\,y)=-\frac{1}{{S}_{0}}\int_{-H}^{0}\int_{{x}_{W}}^{{x}_{E}}v(S-{S}_{0}){\rm{d}}x{\rm{d}}z$$

and can be further decomposed in an overturning (*F*_ov_), azonal gyre (*F*_az_), barotropic (*F*_bt_ ≈ 0) and eddy (*F*_eddy_) contribution:

8 $${F}_{{\rm{ov}}}(\,y)=-\frac{1}{{S}_{0}}\int_{-H}^{0}\left[\int_{{x}_{W}}^{{x}_{E}}{v}^{* }{\rm{d}}x\right]\left[\langle S\rangle -{S}_{0}\right]{\rm{d}}z,$$

9 $${F}_{{\rm{az}}}(\,y)=-\frac{1}{{S}_{0}}\int_{-H}^{0}\int_{{x}_{W}}^{{x}_{E}}v{\prime} S{\prime} {\rm{d}}x{\rm{d}}z,$$

10 $${F}_{{\rm{bt}}}(\,y)=-\frac{1}{{S}_{0}}\int_{-H}^{0}\int_{{x}_{W}}^{{x}_{E}}\hat{v}\left(\hat{S}-{S}_{0}\right){\rm{d}}x{\rm{d}}z,$$

11 $${F}_{{\rm{eddy}}}(\,y)=-\frac{1}{{S}_{0}}\int_{-H}^{0}\int_{{x}_{W}}^{{x}_{E}}\tilde{v}\left(\tilde{S}-{S}_{0}\right){\rm{d}}x{\rm{d}}z.$$

Here, v^*^ is defined as ${v}^{* }=v-\hat{v}$, where *v* is the meridional velocity and $\hat{v}$ ($\hat{S}$) is the section spatially averaged meridional velocity (salinity). The quantity 〈*S*〉 indicates the zonally averaged salinity, and primed quantities ($v{\prime}$ and $S{\prime}$) are deviations from their respective zonal averages. The $\tilde{v}$ and $\tilde{S}$ are the eddy terms for velocity and salinity, respectively, with more details provided in ref. ^83^.

### The five-box AMOC model

The idealized five-box AMOC model dynamically resolves salinities, temperatures and the pycnocline depth (Extended Data Fig. 9). The model has the asymmetric freshwater flux forcing, *E*_A_, as a control parameter. In addition, the model is forced by a varying temperature anomaly $\Delta {T}_{{\rm{n}}}^{a}$ applied to ${T}_{{\rm{n}}}^{a}$ (and also ${T}_{{\rm{s}}}^{a}$ as they are coupled). More details, parameter settings and sensitivity experiments are provided in refs. ^7,30^; the version analysed here does not consider sea-ice insulation effects.

We note that previous versions of the model (including the versions used in refs. ^7,28,30^) produce an artificial freshwater and temperature flux under pycnocline variations. The volume transport between the ts-box and t-box, *q*_S_, is here corrected by substituting in the model equations:

12 $${q}_{S}\longrightarrow {q}_{S}-\frac{{L}_{xA}{L}_{y}}{2}\frac{{\rm{d}}D}{{\rm{d}}t}\,,$$

where *L*_*xA*_ is the zonal extent of the ts-box, *L*_*y*_ is the meridional extent of the ts-box and *D* is the pycnocline depth. The volume transport between the d-box and s-box, which also uses *q*_S_, is left unchanged. This correction does not impact the steady states because $\frac{{\rm{d}}D}{{\rm{d}}t}=0$, but does influence transient responses for considerable pycnocline depth variations.

## Online content

Any methods, additional references, Nature Portfolio reporting summaries, source data, extended data, supplementary information, acknowledgements, peer review information; details of author contributions and competing interests; and statements of data and code availability are available at https://doi.org/10.1038/s41558-026-02730-w.

## Supplementary information

Peer Review File

## Data Availability

The (processed) model output is available via Zenodo at https://doi.org/10.5281/zenodo.21352775 (ref. ^67^). The CMIP6 model output is provided by the World Climate Research Programme’s Working Group on Coupled Modeling.

## Extended data

**Extended Data Table 1:** Simulations performed with the CESM

Simulation name	$\overline{\boldsymbol{F}_{\boldsymbol{H}}}$	Forcing conditions	Collapse onset	AMOC strength	GMST anomaly
	(Sv)		(model year)	(Sv)	(°C)
Extended RCP4.5 #1	0.18	Historical–RCP4.5	–	–	–
Extended RCP8.5 #1		Historical–RCP8.5	2099	7.8	+ 4.15^1^
Extended RCP2.6	0.45	Historical–RCP2.6	–	–	–
Extended RCP4.5 #2		Historical–RCP4.5	2111	8.3	+ 2.19^2^
Extended RCP8.5 #2		Historical–RCP8.5	2061	9.0	+ 2.78^2^
CO_2_ ramp #1	0.45	+0.5 ppm yr^−1^ of CO_2_	–	–	–
CO_2_ ramp #2		+2.5 ppm yr^−1^ of CO_2_	139	9.0	+ 2.02^3^
CO_2_ ramp #3		+5.0 ppm yr^−1^ of CO_2_	78	8.8	+ 1.99^3^

Overview of the available CESM simulations, including the value of $\overline{{F}_{H}}$ and forcing conditions. Where applicable, the timing of AMOC collapse onset and the associated AMOC strength and GMST anomaly (compared to: 1 = Historical of $\overline{{F}_{H}}=0.18$ Sv (1850 – 1899); 2 = Historical of $\overline{{F}_{H}}=0.45$ Sv (1850 – 1899); 3 = PI steady state of $\overline{{F}_{H}}=0.45$ Sv) are also indicated.

## Code availability

The analysis scripts are also available via Zenodo at https://doi.org/10.5281/zenodo.21352775 (ref. ^67^).

## References

[1] Lenton, T. M. et al. *Global Tipping Points Report 2025* (Univ. Exeter, 2025).

[2] Armstrong McKay, D. I. et al. Exceeding 1.5 °C global warming could trigger multiple climate tipping points. *Science* **377**, 7950 (2022). 10.1126/science.abn7950 36074831

[3] Wunderling, N. et al. Global warming overshoots increase risks of climate tipping cascades in a network model. *Nat. Clim. Change* **13**, 75–82 (2023).

[4] Möller, T. et al. Achieving net zero greenhouse gas emissions critical to limit climate tipping risks. *Nat. Commun.* **15**, 6192 (2024). 10.1038/s41467-024-49863-0 PMC11294534 39090087

[5] Rahmstorf, S. et al. Thermohaline circulation hysteresis: a model intercomparison. *Geophys. Res. Lett.* **32**, L23605 (2005).

[6] Kriegler, E., Hall, J. W., Held, H., Dawson, R. & Schellnhuber, H. J. Imprecise probability assessment of tipping points in the climate system. *Proc. Natl Acad. Sci. USA* **106**, 5041–5046 (2009). 10.1073/pnas.0809117106 PMC2657590 19289827

[7] van Westen, R. M., Vanderborght, E. & Dijkstra, H. A. A saddle-node bifurcation may be causing the AMOC collapse in the community Earth system model. *Earth Syst. Dyn.* **16**, 2063–2085 (2025).

[8] Lenton, T. M. et al. Tipping elements in the Earth’s climate system. *Proc. Natl Acad. Sci. USA* **105**, 1786–1793 (2008). 10.1073/pnas.0705414105 PMC2538841 18258748

[9] Dima, M., Lohmann, G., Nichita, D.-R., Knorr, G. & Scholz, P. Structural stability changes of the Atlantic Meridional Overturning Circulation. *npj Clim. Atmos. Sci.* **8**, 73 (2025).

[10] Stocker, T. F. & Schmittner, A. Influence of CO_2_ emission rates on the stability of the thermohaline circulation. *Nature* **388**, 862–865 (1997).

[11] Manabe, S. & Stouffer, R. J. Multiple-century response of a coupled ocean-atmosphere model to an increase of atmospheric carbon dioxide. *J. Clim.* **7**, 5–23 (1994).

[12] Hankel, C. The effect of co2 ramping rate on the transient weakening of the Atlantic Meridional Overturning Circulation. *Proc. Natl Acad. Sci. USA* **122**, 2411357121 (2025). 10.1073/pnas.2411357121 PMC11725785 39715440

[13] Bonan, D. B., Thompson, A. F., Newsom, E. R., Sun, S. & Rugenstein, M. Transient and equilibrium responses of the Atlantic overturning circulation to warming in coupled climate models: the role of temperature and salinity. *J. Clim.* **35**, 5173–5193 (2022).

[14] Nobre, P. et al. Amoc decline and recovery in a warmer climate. *Sci. Rep.* **13**, 15928 (2023). 10.1038/s41598-023-43143-5 PMC10517999 37741891

[15] Curtis, P. E. & Fedorov, A. V. Collapse and slow recovery of the Atlantic Meridional Overturning Circulation (AMOC) under abrupt greenhouse gas forcing. *Clim. Dyn.* **62**, 5949–5970 (2024).

[16] Willeit, M. & Ganopolski, A. Generalized stability landscape of the Atlantic Meridional Overturning Circulation. *Earth Syst. Dyn.* **15**, 1417–1434 (2024).

[17] Schmittner, A. & Stocker, T. F. The stability of the thermohaline circulation in global warming experiments. *J. Clim.* **12**, 1117–1133 (1999).

[18] Ashwin, P., Wieczorek, S., Vitolo, R. & Cox, P. Tipping points in open systems: bifurcation, noise-induced and rate-dependent examples in the climate system. *Philos. Trans. R. Soc. A* **370**, 1166–1184 (2012). 10.1098/rsta.2011.0306 22291228

[19] Wieczorek, S., Xie, C. & Ashwin, P. Rate-induced tipping: thresholds, edge states and connecting orbits. *Nonlinearity* **36**, 3238 (2023).

[20] Waibel, M., Hulbe, C., Jackson, C. & Martin, D. Rate of mass loss across the instability threshold for thwaites glacier determines rate of mass loss for entire basin. *Geophys. Res. Lett.* **45**, 809–816 (2018).

[21] Mouginot, J. et al. Forty-six years of greenland ice sheet mass balance from 1972 to 2018. *Proc. Natl Acad. Sci.USA* **116**, 9239–9244 (2019). 10.1073/pnas.1904242116 PMC6511040 31010924

[22] Boers, N. & Rypdal, M. Critical slowing down suggests that the western greenland ice sheet is close to a tipping point. *Proc. Natl Acad. Sci.USA* **118**, 2024192118 (2021). 10.1073/pnas.2024192118 PMC8166178 34001613

[23] Turney, C. S. et al. Early last interglacial ocean warming drove substantial ice mass loss from antarctica. *Proc. Natl Acad. Sci.USA* **117**, 3996–4006 (2020). 10.1073/pnas.1902469117 PMC7049167 32047039

[24] Stommel, H. Thermohaline convection with two stable regimes of flow. *Tellus* **13**, 224–230 (1961).

[25] Rahmstorf, S. On the freshwater forcing and transport of the Atlantic thermohaline circulation. *Clim. Dyn.* **12**, 799–811 (1996).

[26] Marotzke, J. Abrupt climate change and thermohaline circulation: mechanisms and predictability. *Proc. Natl Acad. Sci. USA* **97**, 1347–1350 (2000). 10.1073/pnas.97.4.1347 PMC34301 10677464

[27] Vanderborght, E., Westen, R. M. & Dijkstra, H. A. Feedback processes causing an AMOC collapse in the community earth system model. *J. Clim.* **38**, 5083–5102 (2025).

[28] Castellana, D., Baars, S., Wubs, F. W. & Dijkstra, H. A. Transition probabilities of noise-induced transitions of the Atlantic Ocean circulation. *Sci. Rep.* **9**, 20284 (2019). 10.1038/s41598-019-56435-6 PMC6937345 31889057

[29] Romanou, A. et al. Stochastic bifurcation of the North Atlantic circulation under a midrange future climate scenario with the NASA-GISS ModelE. *J. Clim.* **36**, 6141–6161 (2023).

[30] van Westen, R. M., Jacques-Dumas, V., Boot, A. A. & Dijkstra, H. A. The role of sea-ice insulation effects on the probability of AMOC transitions. *J. Clim.* **37**, 6269–6284 (2024).

[31] Oh, J.-H. et al. Noise-induced tipping of Atlantic Meridional Overturning Circulation under climate mitigation scenarios. *Nat. Commun.* **16**, 11515 (2025). 10.1038/s41467-025-66494-1 PMC12749622 41390504

[32] Jackson, L. C. et al. Understanding AMOC stability: the North Atlantic hosing model intercomparison project. *Geosci. Model Dev.* **16**, 1–32 (2023).

[33] van Westen, R. M., Kliphuis, M. & Dijkstra, H. A. Physics-based early warning signal shows that AMOC is on tipping course. *Sci. Adv.* **10**, 1189 (2024). 10.1126/sciadv.adk1189 PMC10857529 38335283

[34] van Westen, R. M., Vanderborght, E., Kliphuis, M. & Dijkstra, H. A. Physics-based indicators for the onset of an AMOC collapse under climate change. *J. Geophys. Res. Oceans* **130**, e2025JC022651 (2025).

[35] Drijfhout, S., Angevaare, J., Mecking, J., van Westen, R. M. & Rahmstorf, S. Shutdown of Northern Atlantic overturning after 2100 following deep mixing collapse in CMIP6 projections. *Environ. Res. Lett.* **20**, 094062 (2025).

[36] Weijer, W. et al. Stability of the Atlantic Meridional Overturning Circulation: a review and synthesis. *J. Geophys. Res. Oceans* **124**, 5336–5375 (2019). 10.1029/2019JC015152 PMC6853260 31763114

[37] Dijkstra, H. A. et al. Multi-stability of the present-day Atlantic Meridional Overturning Circulation. *WIREs Clim. Change* **17**, 70049 (2026).

[38] Weijer, W., Cheng, W., Garuba, O. A., Hu, A. & Nadiga, B. T. CMIP6 models predict significant 21st century decline of the Atlantic Meridional Overturning Circulation. *Geophys. Res. Lett.* **47**, 2019–086075 (2020).

[39] Bonan, D. B. et al. Observational constraints imply limited future Atlantic Meridional Overturning Circulation weakening. *Nat. Geosci.* **18**, 479–487 (2025).

[40] Dijkstra, H. A. & Westen, R. M. The probability of an AMOC collapse onset in the twenty-first century. *Annu. Rev. Mar. Sci.* **18**, 23–46 (2026). 10.1146/annurev-marine-040324-024822 40720806

[41] Liu, W., Xie, S.-P., Liu, Z. & Zhu, J. Overlooked possibility of a collapsed Atlantic Meridional Overturning Circulation in warming climate. *Sci. Adv.* **3**, 1601666 (2017). 10.1126/sciadv.1601666 PMC5217057 28070560

[42] van Westen, R. M. & Dijkstra, H. A. Asymmetry of AMOC hysteresis in a state-of-the-art global climate model. *Geophys. Res. Lett.* **50**, e2023GL106088 (2023).

[43] Ma, Q. et al. Revisiting climate impacts of an AMOC slowdown: dependence on freshwater locations in the North Atlantic. *Sci. Adv.* **10**, 3243 (2024). 10.1126/sciadv.adr3243 PMC11578178 39565857

[44] Rahmstorf, S. & Ganopolski, A. Long-term global warming scenarios computed with an efficient coupled climate model. *Clim. Change* **43**, 353–367 (1999).

[45] Dijkstra, H. A. & Westen, R. M. The effect of Indian Ocean surface freshwater flux biases on the multi-stable regime of the AMOC. *Tellus A Dyn. Meteorol. Oceanogr.* **76**, 90–100 (2024).

[46] Mehling, O., Vanderborght, E. & Dijkstra, H. A. Critical freshwater forcing for AMOC tipping in climate models—compensation matters. *Earth Syst. Dyn.* **17**, 563–579 (2026).

[47] Hawkins, E. et al. Bistability of the Atlantic overturning circulation in a global climate model and links to ocean freshwater transport. *Geophys. Res. Lett.* **38**, 10605 (2011).

[48] Hu, A. et al. Role of the Bering Strait on the hysteresis of the ocean conveyor belt circulation and glacial climate stability. *Proc. Natl Acad. Sci. USA* **109**, 6417–6422 (2012). 10.1073/pnas.1116014109 PMC3340043 22493225

[49] van Westen, R. M. & Dijkstra, H. A. Persistent climate model biases in the Atlantic Ocean’s freshwater transport. *Ocean Sci.* **20**, 549–567 (2024).

[50] Boot, A. A. & Dijkstra, H. A. Physics of AMOC multistable regime shifts due to freshwater biases in an EMIC. *Earth Syst. Dyn.* **16**, 1221–1235 (2025).

[51] van Westen, R. M. & Baatsen, M. L. European temperature extremes under different AMOC scenarios in the community Earth system model. *Geophys. Res. Lett.* **52**, e2025GL114611 (2025).

[52] Srokosz, M. & Bryden, H. Observing the Atlantic Meridional Overturning Circulation yields a decade of inevitable surprises. *Science* **348**, 1255575 (2015). 10.1126/science.1255575 26089521

[53] Smeed, D. A. et al. The North Atlantic Ocean is in a state of reduced overturning. *Geophys. Res. Lett.* **45**, 1527–1533 (2018).

[54] van Westen, R. M., Wiel, K., Falkena, S. K. & Selten, F. Changing European hydroclimate under a collapsed amoc in the community Earth system model. *Hydrol. Earth Syst. Sci.* **29**, 6607–6630 (2025).

[55] Menary, M. B. et al. Aerosol-forced amoc changes in CMIP6 historical simulations. *Geophys. Res. Lett.* **47**, 2020–088166 (2020).

[56] Hassan, T., Allen, R. J., Liu, W. & Randles, C. A. Anthropogenic aerosol forcing of the Atlantic Meridional Overturning Circulation and the associated mechanisms in CMIP6 models. *Atmos. Chem. Phys.* **21**, 5821–5846 (2021).

[57] Nikurashin, M. & Vallis, G. A theory of the interhemispheric meridional overturning circulation and associated stratification. *J. Phys. Oceanogr.* **42**, 1652–1667 (2012).

[58] Wolfe, C. L. & Cessi, P. Salt feedback in the adiabatic overturning circulation. *J. Phys. Oceanogr.* **44**, 1175–1194 (2014).

[59] Walin, G. On the relation between sea-surface heat flow and thermal circulation in the ocean. *Tellus* **34**, 187–195 (1982).

[60] Marshall, J., Jamous, D. & Nilsson, J. Reconciling thermodynamic and dynamic methods of computation of water-mass transformation rates. *Deep Sea Res. Part I* **46**, 545–572 (1999).

[61] Grist, J. P., Marsh, R. & Josey, S. A. On the relationship between the North Atlantic Meridional Overturning Circulation and the surface-forced overturning streamfunction. *J. Clim.* **22**, 4989–5002 (2009).

[62] Desbruyères, D. G., Mercier, H., Maze, G. & Daniault, N. Surface predictor of overturning circulation and heat content change in the subpolar north atlantic. *Ocean Sci.* **15**, 809–817 (2019).

[63] Marsh, R. Recent variability of the North Atlantic thermohaline circulation inferred from surface heat and freshwater fluxes. *J. Clim.* **13**, 3239–3260 (2000).

[64] Groeskamp, S. et al. The water mass transformation framework for ocean physics and biogeochemistry. *Annu.Rev. Mar. Sci.* **11**, 271–305 (2019). 10.1146/annurev-marine-010318-095421 30230995

[65] Lin, Y.-J., Rose, B. E. & Hwang, Y.-T. Mean state AMOC affects AMOC weakening through subsurface warming in the Labrador Sea. *J. Clim.* **36**, 3895–3915 (2023).

[66] Hankel, C., Cheng, W. & Bitz, C. M. Arctic sea ice meltwater as a forcing and feedback on the Atlantic Meridional Overturning Circulation. *J. Clim.* **38**, 1821–1839 (2026).

[67] van Westen, R., Börner, R. & Dijkstra, H. AMOC tracking under climate change. *Zenodo*10.5281/zenodo.21352775 (2026).

[68] Willeit, M., Ganopolski, A., Edwards, N. R. & Rahmstorf, S. Surface buoyancy control of millennial-scale variations in the Atlantic meridional ocean circulation. *Clim. Past* **20**, 2719–2739 (2024).

[69] Brüggemann, N. & Katsman, C. A. Dynamics of downwelling in an eddying marginal sea: contrasting the eulerian and the isopycnal perspective. *J. Phys. Oceanogr.* **49**, 3017–3035 (2019).

[70] Dai, J., Xu, F., Wright, J. S., Huang, R. X. & Huang, X. Subpolar North Atlantic sea surface salinity as an amoc mean state indicator. *npj Clim. Atmos. Sci.* **8**, 308 (2025).

[71] Huisman, S. E., Den Toom, M., Dijkstra, H. A. & Drijfhout, S. An indicator of the multiple equilibria regime of the Atlantic Meridional Overturning Circulation. *J. Phys. Oceanogr.* **40**, 551–567 (2010).

[72] Hansen, J. E. et al. Global warming in the pipeline. *Oxf. Open Clim. Change* **3**, 008 (2023).

[73] Foster, G. & Rahmstorf, S. Global warming has accelerated significantly. *Geophys. Res. Lett.* **53**, e2025GL118804 (2026).

[74] van Westen, R. M., Kliphuis, M. & Dijkstra, H. A. Collapse of the Atlantic Meridional Overturning Circulation in a strongly eddying ocean-only model. *Geophys. Res. Lett.* **52**, e2024GL114532 (2025).

[75] Großelindemann, H., Castruccio, F. S., Danabasoglu, G. & Biastoch, A. Long-term variability and trends in the Agulhas leakage and its impacts on the global overturning. *Ocean Sci.* **21**, 93–112 (2025).

[76] Caesar, L., Rahmstorf, S., Robinson, A., Feulner, G. & Saba, V. Observed fingerprint of a weakening Atlantic Ocean overturning circulation. *Nature* **556**, 191–196 (2018). 10.1038/s41586-018-0006-5 29643485

[77] Michel, S. L. et al. Deep learning based reconstructions of the Atlantic Meridional Overturning Circulation confirm twenty-first century decline. *Environ. Res. Lett.* **20**, 064036 (2025).

[78] Slyman, K. & Jones, C. K. Rate and noise-induced tipping working in concert. *Chaos* **33**, 013119 (2023). 10.1063/5.0129341 36725643

[79] Smith, R. et al. *The Parallel Ocean Program (POP) Reference Manual* (NCAR, 2010).

[80] Neale, R. B. et al. The mean climate of the Community Atmosphere Model (CAM4) in forced SST and fully coupled experiments. *J. Clim.* **26**, 5150–5168 (2013).

[81] Hunke, E. & Lipscomb, W. *The Los Alamos Sea Ice Model, Documentation and Software* Technical Report LA-CC-06-012 (2008).

[82] McDougall, T. J. & Barker, P. M. Getting started with TEOS-10 and the Gibbs Seawater (GSW) oceanographic toolbox. *Scor/iapso WG* **127**, 1–28 (2011).

[83] Jüling, A., Zhang, X., Castellana, D., Von Der Heydt, A. S. & Dijkstra, H. A. The Atlantic’s freshwater budget under climate change in the Community Earth System Model with strongly eddying oceans. *Ocean Sci.* **17**, 729–754 (2021).

[84] Elson, P. et al. SciTools/cartopy: REL: v0.24.1. *Zenodo*10.5281/zenodo.13905945 (2024).

