# Supplementary material for: Failure to track a stable AMOC state under rapid climate change
Source: Nat Clim Chang. 2026 Aug 13;16(10):1195–206. doi: 10.1038/s41558-026-02730-w (PMC13638462; doi:10.1038/s41558-026-02730-w)
Supplement: Supplementary file 1 [file 41558_2026_2730_MOESM1_ESM.pdf]

nature portfolio

Peer Review File

Failure to track a stable AMOC state under rapid climate change

Corresponding Author: Dr René van Westen

This file contains all reviewer reports in order by version, followed by all author rebuttals in order by version.

Version 0:

Reviewer comments:

Reviewer #1

(Comments for the Author)
This is a review of the manuscript entitled “Failure to track a stable AMOC state under rapid climate change” by van Westen, Börner and Dijkstr. The paper discusses AMOC evolution under different rates of warming and constant freshwater forcing and the timing of crossing critical thresholds in one model mainly but compares to other models as well.

The paper includes some very important & original results and should be published, however there are some necessary adjustments, I would not call them minor or major, but just necessary. The conclusions are robust but some questions exist.

The main issue is that the paper is not very clearly written which is unusual for these authors. At some places it is a lot more detailed and in other places too brief. It would be straight forward to go through it and just tighten up the text. Additionally, a table with crossing times, emissions or concentration levels and warming levels would help summarize the results better, particularly if it includes information about type of forcing (idealized, CO2 only, RCP forcing etc). The use of the box model as is now is not necessary, otherwise it needs to be expanded.

The abstract (and possibly the title) do not convey clearly the points of the paper. In my opinion, and the authors may disagree and it is their decision to make, the abstract should cast more emphasis in the rate dependency of the “realistic” scenarios and less so in the slow (highly idealized) experiment. The last sentence of the abstract is key. There will be more papers around this theme.

Other comments
- Lines 60-62: AMOC destabilization is not just through the larger scale salt-advection feedback. Noise induced instability has been shown in models as well (e.g. Romanou et al. 2023; Oh et al. 2025). Local feedbacks and processes may disrupt the operation of the large-scale salt-advection feedback and lead to abrupt shifts in AMOC. This is why several models with a negative salt-advection feedback, get AMOC collapse.
- Lines 63-65: this is only evidence from CESM1 while other models show that AMOC can collapse for smaller rates of freshening, sometimes Greenland ice sheet melt is not necessary (e.g. Romanou et al. 2023)
- Lines 72-73: the baseline simulation (reported in van Westen et al 2024; Science Advances) takes about 800 years to collapse under a flux which is 0.03Sv/yr. This is a huge freshwater forcing -not really supported by current climate evidence. Also, freshwater flux was applied over a large area of the North Atlantic 20-50N. Here AMOC collapse starts after 1700 years. These points need to be made clearly in the text (in the Discussion or here) to aid readers parse the importance of the freshwater forcing in different models.
- Lines 84-86: Freshwater compensation to conserve ocean salinity. Although it is general practice it is incorrect and needs to be shown that adding salt (increasing mixing) elsewhere does not affect currents and transports. The practice is invoked because many models, including CESM1, have rig-lid approximations and cannot add a physical freshwater flux in the model and must resort to adding or subtracting salt. However, adding/subtracting so much salt over prolonged periods of time has implications for local stratification profiles, mixing etc. This is a moot point however, since the authors cannot do anything about it. But I would welcome a very brief discussion on the importance of the salt correction.
- 0.5Sv over ~1500 yrs and over 20-50N. This is a very large freshwater flux for a very large region in the domain. In the discussion it would be nice to compare it to SMB changes for the Greenland ice sheet.
- Lines 98-100: the historical and future CESM runs have additional freshwater forcing. I would like to see in the Supplement a figure with comparison of the total forcing in these runs.

- Fig. 1c: the warming in this simulation seems too high. For example at 400 years, CO2 atm concentrations are 284+0.5ppm/yr*400=484ppm and the temperature change is ~1.8deg! And you add freshwater but the AMOC declines very little. Why?
Some clear quantification of these numbers would help (preferably in a table?)
- Fig. 1d: The sensitivity to freshwater forcing in the slow ramp-up (PI CO2) is stronger than due to CO2 forcing in the historical. That is probably because all these runs are branched off an already destabilized AMOC. Is this a fair comparison though? Or maybe I am missing something -apologies I was a little lost as to what the forcings in each run are. Also, how do you define the "tipping" threshold in the RCP8.5 being 2.8 degrees? There is tipping in RCP4.5 at 2.2C but I do not see tipping in RCP8.5 at 2.8C which is contrary to what you are trying to convey. There is steady weakening in AMOC but not tipping. Please clarify. Therefore I do not agree with lines 127-128.
- Lines 150-155: what is the contribution of the wind driven component of the AMOC?
- Figure S1a: why is there (substantial!) convection area increase in the ramp-up case? (small inlet figure). I would expect freshening. Where does this freshwater go? Mixes down too fast (relative to the forcing) or is advected away too fast? Or is salt advected in the region (due to salt correction)?
- Line 194: how important is the area of the calculations 40-65N? Surely, this area is much broader than the convection sites. I know there is justification for this in earlier papers, but please repeat it here too.
- Line 218-220. It is interesting that AMOC strengthens and MLDs deepen (Fig. S3d) while there is continuous warming. There is clearly a shift here but if it is not relevant to the topic of the paper, there is no need to discuss. In any case, Labrador Sea mixing is shutdown from the start of the simulation (sea ice covered) but then resumes presumably due to warming. Even so, the mixing is too shallow to feed in AMOC.
- It is great that you point out in Line 235 that FovS is only applicable as a criterion for AMOC stability under equilibrium, however, it is used broadly by the community as an indicator even under transient warming. Perhaps you want to highlight this point a little more.
- Line 253: important finding: there is no threshold but rather behavior is controlled by the warming rate. However, I would rephrase this that the critical threshold is rate dependent. If there is tipping behavior, there is a critical threshold. It is just not universal, and I agree is not a certain GMST. Other studies under review right now are showing the same result.
- Lines 282-285 and later: unclear what the conclusion is here from the multi-model comparison.
- Figure 5: it seems to me that there is a strong shift to the south of the ITCZ even though there is no AMOC weakening in the ramp-up run. Can you explain the patterns? And the surface salinity increases significantly, consistent with AMOC strengthening. I am not sure what is going on here. Is the ramp up too slow and the adjustments too fast?
- The use of the box model is unclear to me. Please explain what simulations you are fitting it to. If they are not the same simulations as the ones you used before, then I do not see what this section adds to the paper. This section would actually merit its own paper, I think.

Minor comments
- Line 40: distinguish studies discussed here. Mechanism is different in 13-15 references. Romanou study is about 2xCO2 and warming levels are exactly the same as pointed out here.
- Figure 1 titles are corrupted. Please go over the captions and figure legends closely.

Anastasia Romanou

Reviewer #2

(Comments for the Author)
Review of "Failure to track a stable AMOC state under rapid climate change"

This study uses CESM simulations to demonstrate that the stability of the Atlantic Meridional Overturning Circulation (AMOC) is critically dependent on the rate of radiative forcing, not just the final level of global warming. Under a very slow CO2 ramp-up (0.5 ppm/yr), the AMOC remains stable up to +5.5°C of warming. In contrast, under the faster RCP4.5 and RCP8.5 scenarios, the AMOC collapses at much lower warming levels (+2.2°C and +2.8°C). The authors identify a stabilizing mechanism involving enhanced evaporation, reduced sea-ice extent, and a salinifying North Atlantic Deep Water (NADW), which allows the AMOC to track its stable state under slow forcing. An idealized box model is used to provide a dynamical perspective on this rate-dependent tipping. While rate-dependent effects have been discussed in conceptual models (e.g., Stocker & Schmittner, 1997), this study provides a clear demonstration in a comprehensive, state-of-the-art Earth System Model (CESM). The core finding that a warming threshold is not a robust predictor of AMOC collapse and that the forcing rate is the critical control parameter challenges a widely held paradigm. The result that the AMOC can remain stable at +5.5°C under slow forcing, while collapsing at +2.2°C under faster forcing, is striking and has profound implications for how we assess and communicate climate tipping risks. This work effectively shifts the focus from "how much warming is too much?" to "how fast is too fast?".
The methodology is sound and appropriate for the research question. The conclusions are well-supported by the model analysis. The paper is well-structured and the narrative flows logically from results to discussion. However, some sections could be streamlined to better serve the main argument (see specific comments below). I recommend publication after minor revisions.

1. A key question arising from this single-model study is the generalizability of its quantitative findings. As the authors note, the experiment is initialized from a specific hosed equilibrium (0.45 Sv), which places the AMOC in a particularly sensitive state. The robustness of the critical warming rate, and even the detailed evolution of the AMOC (e.g., the activation of

Labrador Sea convection around year 800), would likely be sensitive to the choice of model and its parameterization schemes, particularly those for sea ice and deep convection. Models with different representations of deep convection may not exhibit the same sharp AMOC strengthening upon sea-ice retreat. The authors' use of an idealized box model (Figure 6) is a valuable step towards demonstrating the broader validity of the rate-dependent mechanism. However, a question remains: Can the warming eventually overcome the stabilizing effects of salinification under any forcing rate, or does the existence of a slow enough pathway imply an absolute limit to AMOC resilience?

2. Baker et al. (2025) present a compelling, global-scale mechanism for AMOC resilience, arguing that persistent Southern Ocean winds sustain a weakened, but not collapsed, AMOC by driving a Pacific Meridional Overturning Circulation (PMOC). Their findings provide a fascinating complement to this study. Baker et al. emphasize a global, mechanical constraint (Southern Ocean winds and the necessity of a PMOC for a full collapse), while van Westen et al. focus on regional, buoyancy-driven processes in the North Atlantic that allow the AMOC to track its stable state. These perspectives may not be contradictory but operate on different scales. A collapsed AMOC (as per Baker et al.) is a global state requiring a PMOC. Van Westen et al. show that even under extreme warming, the AMOC avoids this global collapse if the forcing is slow enough for the North Atlantic to adjust. I am wondering how the rate-dependent buoyancy adjustments the authors identify might influence the likelihood and strength of PMOC emergence, thereby linking their regional mechanism to the global-scale constraints highlighted by Baker et al. Does a faster forcing rate, by maintaining a weaker NADW outflow, contribute to the development of a PMOC?

3. The study might benefit from a more explicit connection to the work of Dai et al. (2025), who propose subpolar North Atlantic sea surface salinity (SPNA SSS) as an AMOC indicator. While the study show a salinifying NADW under slow warming (implying stability), Dai et al. argue that a significant freshening (e.g., ~2 PSU) would be needed for a substantial AMOC slowdown. This apparent contrast highlights the rate-dependent nature of the problem: a slow freshening might allow for compensating mechanisms (such as those identified here), while a rapid freshening could lead to collapse. The authors should discuss how their rate-dependent framework could reconcile these different perspectives. Specifically, is the concept of a 'critical freshwater volume' for collapse itself a function of the forcing rate? A slow, steady freshwater input might be balanced by the stabilizing mechanisms described (enhanced evaporation, NADW salinification), whereas the same total volume delivered rapidly could overwhelm the system and trigger a collapse. This would be a valuable extension of the freshwater hysteresis framework into the rate-dependent regime.

References:
Baker, J. A., et al. (2025). Continued Atlantic overturning circulation even under climate extremes. Nature.
Dai, J., et al., (2025). Subpolar North Atlantic sea surface salinity as an AMOC mean state indicator. npj Climate and Atmospheric Science.
Stocker, T. F., & Schmittner, A. (1997). Influence of CO2 emission rates on the stability of the thermohaline circulation. Nature.

Reviewer #3

(Comments for the Author)
Summary:
This manuscript examines the evolution of the AMOC in a new climate modeling experiment in which atmospheric CO_2 is increased very gradually over a few millennia and compares it to the AMOC evolution in the same model under more common Representative Concentration Pathways (which have a variety of levels and rates of greenhouse gas increase). The biggest takeaway from the comparison of these different experiments is that strong AMOC state can be maintained under very high levels of global mean warming when the rate of CO_2 change is very slow, while the AMOC would collapse at a much lower level of global mean warming under faster and more extreme forcing scenarios. This has the implication that global mean surface temperature “thresholds” for an AMOC tipping point are not meaningful, which changes how we think about the usefulness of policies aimed at limiting global warming to a certain level. The authors then perform two major categories of analysis to try to identify the physical mechanisms that stabilize the AMOC when the forcing rate is slow but fail when it is fast. Finally, they illustrate that in a simple conceptual model of the AMOC forced with temperature and freshwater changes, the collapse of the AMOC can be induced by faster rates of warming.
Overall, I think the main point of this paper (that a true global warming threshold does not exist for an AMOC tipping point because the tipping point is forcing scenario-dependent) is impactful and novel, both for the scientific field and the general public. However, I do not believe the authors’ secondary claim that the forcing rate is the primary determinant of whether a tipping point is crossed is fully justified, and significant work is needed to make the analysis more complete, logically sound, and supportive of their conclusions. In particular, because the study compares a slow CO_2 ramping rate to RCP scenarios in which not only the forcing rate, but also the forcing magnitude and types of forcing agents are different, the authors do not convincingly show that the differing AMOC evolutions are actually attributable to the different forcing rates rather than these other factors. In some sections (which I elaborate on below), they identify mechanisms that stabilize the AMOC in their slow CO_2 ramp, but do not show or explain why such mechanisms would not stabilize the AMOC under faster forcing rates, thus failing to connect their mechanistic insights to the forcing rate of change itself (which is central to their argument). Finally, I do not think the “dynamical perspective” section that uses the AMOC box model provides much scientific insight as it currently stands, because the mechanisms of rate-induced AMOC collapse in the conceptual model are not the same as those in the Earth System Model, so I recommend the authors either remove this section, or think carefully about how to re-formulate the box model to be more relevant for interpreting the CESM results. If the authors can resolve these issues, I believe the mechanistic explanations for the differing AMOC behaviors in the different modeling experiments have the potential to significantly deepen our understanding of rate-sensitivity of the AMOC and whether such rate sensitivity is

enough to cause a tipping point. Below, I provide general comments for each of the three results sections, and then more specific comments which may also help resolve the general comments.
General comments:
1) Isolating the role of forcing rate: As mentioned above, one of the major ways that the authors' analysis does not necessarily support their conclusions is that the experiments they compare (the CO_2 ramp, the RCPs in CESM, and the SSPs in other CMIP models) have not only different forcing rates, but also different forcing magnitudes, forcing agents, and even declining forcing (in the case of RCP2.6), so the differing mechanisms of AMOC stabilization or collapse that they explore may not necessarily be due to different forcing rates. In other words, they do not convincingly isolate the effect of the rate of forcing increase, despite this being central to their main conclusions. There are a few ways they could overcome this. One would be to run an additional experiment in CESM where CO_2 is ramped to the same final value as in their current experiment, but at a much faster ramping rate (perhaps comparable to the rate of increase in an intermediate or high RCP scenario). The authors could then focus the bulk of their analysis on comparing the two CO_2 ramps, but insofar as they identify similar features in the "fast" ramp as in the RCP and SSP scenarios, this would strengthen the claim that the collapse of AMOC in intermediate and high future emissions scenarios across CMIP models is driving by the failure of the AMOC to track its otherwise stable steady state. This would also eliminate the possibility that other forcing agents in the RCP/SSP scenarios are playing a role (for example, Northern Hemisphere aerosol changes, which may be relevant for surface buoyancy fluxes over the North Atlantic). To me this would be the most elegant solution, and is the one I recommend, but I recognize it requires an additional modeling experiment with additional computational resources. Another option would be to analyze segments of the CO_2 ramp and RCP and SSP scenarios that have similar levels of CO_2 change, but different rates of change (for example, the first ~50 years of RCP8.5, ~100 years of RCP4.5, and 600 years of the CO_2 ramp, all up until CO_2 reaches ~580 ppm). Then if they identify that key stabilizing mechanisms found in the CO_2 ramp are weaker or overcompensated by other mechanisms in the "faster" forcing scenarios (and can explain why this is related to the rate of forcing change), then I believe they would have a convincing argument that the eventual AMOC collapse in the higher RCP scenarios is due to forcing-rate-dependent stabilizing mechanisms. The third option (and this would be the bare minimum), is to clearly show that the mechanisms stabilizing the AMOC in the slow CO_2 ramp do not operate in the RCP/SSP experiments for reasons directly related to the different forcing rate of change. To some extent they do this in their analysis of surface water mass transformation by showing that the AMOC collapse occurs in the high emissions scenarios because the surface densities change faster than interior densities (thus shutting down surface WMT), which is clearly related to the interior ocean being out of equilibrium under faster forcing scenarios (though some key analyses are missing here, as I describe next).
2) Section on water mass transformation: I believe the results here do support the conclusion that the forcing rate influences whether the AMOC collapses or not, but some additional analyses and clarity are needed to make this evidence stronger. First, if the authors plan to use the factors contributing to Ψ_{NADW} to explain the differences in AMOC behavior across the CO_2 ramp and RCP experiments, they need to show that Ψ_{NADW} at least qualitatively matches the AMOC behavior in all experiments analyzed, in particular that it captures the greater AMOC weakening in the RCPs. To do this, I recommend they plot Ψ_{NADW} for the RCPs, not just for the CO_2 ramp. (This would also help justify their approach of looking at Ψ_{NADW} to diagnose AMOC changes, which at the moment seems rather dubious given that Ψ_{NADW} has a different trend than the AMOC index in the CO_2 ramp--clearly positive, as opposed to near-zero). Then, they could break down Ψ_{NADW} changes into changes from Bflux and changes from the area of deep water production shrinking (in both the CO_2 ramp and the RCP experiments), where I expect they will find that the key difference between the slow CO_2 ramp and the fast RCPs is the shrinking of the area where surface densities are greater than σ_{2max}, rather than differences in Bflux. After doing this, the authors can then use Figure S1 to confirm that the differences in Ψ_{NADW} across the experiments, which are correlated with and perhaps driving the differences in the AMOC strength, are due to forcing rate-dependent effects (lagged adjustment of interior ocean densities under faster forcing changes). By fully analyzing the surface water mass transformation in the RCPs in addition to the CO_2 ramp, this series of analyses will conclusively show that the greater AMOC weakening in the RCPs can be traced back to the faster forcing rate leading to differing WMT evolution.
3) Section on freshwater budget: In this section the authors analyze changes in $F_{ov,S}$ in the slow CO_2 ramp to identify another stabilizing feedback (salinification of returning NADW) that keeps the AMOC strong under slow forcing changes. However, their eventual claims that this stabilizing mechanism fails under faster forcing changes are not supported, as they do not analyze this mechanism for the high-RCP scenarios where the AMOC collapses. Specifically, the authors do not show $F_{ov,S}$ or NADW salinity changes (which they claim help explain the $F_{ov,S}$ trend) in the RCP8.5 scenario, and therefore do not provide any evidence that the stabilizing NADW mechanism is failing in RCP8.5, nor do they offer any clear reason why this failure would be caused by a faster forcing rate. To put it more directly: if the authors want to claim that the AMOC collapses in RCP8.5 but not in other scenarios because the higher rate of forcing change causes the stabilizing NADW mechanism to fail, they need to show a) that the NADW is not salinifying in RCP8.5 or that it is overcompensated by a different destabilization process and b) that whatever process they find in (a) is caused by the higher rate of forcing change. For example, could it be the case that NADW does not salinify in RCP8.5 runs because precipitation increases occur faster than evaporation increases (due to the slow adjustment of SSTs driving lagging behind the forcing under fast changes), leading to a net increase in the FW flux over the NADW formation regions? This is just an example of an analysis/explanation that would better support the authors' claim. The authors do a better job of establishing this logical chain in their discussion of WMT in the prior section, which they could model here: they show that NADW production cannot be sustained in the RCPs because the area of deep water formation (where surface outcroppings > σ_{2max}) disappears, and importantly they are able to show that this is a "rate-induced" effect. I recommend the authors try to establish a similar logical chain in this section. To do this, they need to perform similar analyses of $F_{ov,S}$ and its drivers in their experiments where the AMOC collapses, and show a forcing-rate-dependence of their proposed stabilizing mechanism. In fact, I do not understand why the authors only chose to analyze scenarios where the AMOC remained "sufficiently strong" (line 269) when their goal is to connect the collapse of the AMOC in high emissions scenarios to rate-dependent stabilizing mechanisms.

In addressing the above, I think the authors could also simplify this section by only focusing on key analyses (but performing

these analyses for all CESM experiments). Some analyses in this section were confusing, seemed to stray away from core AMOC-stability mechanisms, and might be extraneous. These included the discussion of freshwater convergence in the Atlantic, which is not as clearly tied to AMOC stability as $F_{ov,S}$ and the discussion of interbasin exchanges, which I mention below. I recommend focusing solely on $F_{ov,S}$, identifying what causes changes in it (presumably salinifying NADW), and whether those changes can be tied to surface perturbations.

4) Dynamical perspective: In this section the authors use a conceptual box model of the AMOC to show that crossing an AMOC tipping point depends on the rate of warming when freshwater changes are coupled to temperature changes. Though this model is updated from a previous iteration to include competing freshwater and temperature effects on the AMOC under anthropogenic climate change, it is not clear to me what new insights are generated from it with respect to interpreting the results in the rest of the manuscript. This is because the conceptual model (at least by my understanding) does not actually capture the key proposed mechanisms by which the AMOC remains stable under gradual warming in CESM: simultaneous adjustment of the interior and surface ocean densities that allows for continued surface WMT, and salinification of NADW due to enhanced basin-wide evaporation. Rather, the stabilization occurs simply by one forcing (freshwater) being prescribed to counteract the effects of another (northern temperature increase) on the AMOC. Moreover, that the freshwater forcing parameter EA is prescribed to decrease linearly with warming actually suppresses/goes against one of the most important rate-induced forcings on the AMOC: that surface freshwater fluxes over the North Atlantic tend to increase with warming on short/fast timescales, either due to enhanced P-E (e.g., Trenberth 2011) or sea ice meltwater changes (Hankel, 2025). Thus, it seems that the conceptual model more so serves to illustrate what a rate-induced tipping point is rather than capture the rate-dependent mechanisms governing the AMOC. If the physics of the conceptual model are actually more closely aligned with the AMOC-stabilizing mechanisms in CESM than I realize, then hopefully the authors can clarify this. Otherwise, I recommend that the authors only include this section in their revised manuscript if they can find a way for their model to capture the actual mechanisms of rate-sensitive AMOC stabilization found in CESM. If they can do so, then this would strengthen their claim that the AMOC behavior in CESM is actually a rate-induced tipping point, but if this cannot be done, then I think the manuscript already has offers significant insight without including this section (provided the authors resolve the other issues mentioned above).

Specific comments:

1) Lines 85—97: why not start from a regular PI climate without any hosing? While I understand it may be useful to start from an AMOC state that is closer to its tipping point, this means that all the simulations start from a climate that may not be realistic/representative of true preindustrial conditions.

2) Methods: Were the statistical PI steady states of the AMOC (at FH=.18 Sv and .45) obtained by branching from the gradual hosing experiment (van Westen & Dijkstra, 2024) and continuing the experiment with those hosing values fixed in time for a while, in order to ensure that the AMOC is indeed in equilibrium with the hosing value? While the hosing rate of increase in van Westen & Dijkstra 2024 is indeed very slow, the circulation could still be modestly out of equilibrium with this very.

3) Line 105: How do the authors define “tipping point” here, when they say that the AMOC reaches a tipping point in a given year? In my view, I would not necessarily assume that the AMOC collapse in the RCP scenarios is a “tipping point” for a few reasons: 1) while the previous work by the same authors shows that the AMOC has multiple equilibria with respect to freshwater forcing, we do not known that it has multiple equilibria with respect to greenhouse gas forcing, 2) relatedly, the collapsed state that is found in RCP4.5 and 8.5 may not be a permanent stable steady state, as much work has shown the AMOC can still recover after several centuries in CESM and other models (without any forcing changes; Bonan et al, 2022; Nobre et al., 2023; Curtis & Fedorov, 2024), and 3) even if the AMOC does undergo a tipping point in the RCP scenarios, it is not clear to me how one would define when this happens, as the circulation is surely not in equilibrium with the forcing (a point the authors make later). It would be best to use careful language here that acknowledges this, and perhaps define this as an AMOC collapse but not necessarily a tipping point.

4) Line 107: Why is the global warming threshold for the AMOC tipping point higher for RCP8.5 than for RCP4.5 if the AMOC should be even further from tracking its equilibrium in RCP8.5 (since greenhouse gases change faster in this scenario)? I would expect, based on the authors’ conclusions later in the manuscript, that the faster the forcing rate of change, the lower the global warming tipping point threshold.

5) Line 119-121: This is quite peculiar/counterintuitive—can the authors briefly explain why the logarithmic relationship between CO_2 concentrations and GMST fails in this case (or cite other literature that does)?

6) Line 125—126: The RCP scenarios also have other forcing agents changing (other GHGs, aerosols, etc.). I recommend the authors discuss these differences briefly and whether they would be expected to significantly influence the AMOC weakening—if so, then we wouldn’t necessarily expect the AMOC to collapse at the same GMST in the CO_2 as in the RCPs.

7) Figure 3: Why do the quantities Ψ_{surf} etc in the legend of panel (a) have delta symbols in front of them? This would suggest to me that these quantities are differences from some baseline, but I believe what is shown in this panel are the absolute surface WMT quantities. In Panel (d), conversely, I wonder if these legend labels should have delta symbols in front since what is shown are differences compared to the first 50 years of the simulation.

8) Line 164—169: These lines describing the causes of the NADW changes are a bit confusing, and I suggest the authors re-write them for clarity. I think the main point they should be trying to make is that Ψ_{NADW} can be influenced by changes in surface buoyancy fluxes and changes in the surface area of isopycnals outcropping with densities greater than σ_{2max}. The strengthening of Ψ_{NADW} appears to be contributed to by both terms, with surface buoyancy fluxes becoming more negative (Fig 3c) and the area meeting the density threshold also increasing Fig. S1 (unless the sign of buoyancy fluxes is different than what I think, in which case the authors should clarify). This understanding is somewhat obfuscated by the fact that the authors say “surface buoyancy fluxes change accordingly to maintain an adiabatic AMOC”, which to me implies that surface fluxes are compensating for a change in $>\sigma_{2max}$, when in fact, the two factors are working in the same direction. Relatedly, the authors comment on σ_{2max} and the surface densities changing at the same rate, but don’t comment on the area greater than σ_{2max}, which is what actually matters for calculating Ψ_{NADW}.

9) Lines 172—173: The RCPs and the CO_2 ramping experiment have not only different rates of forcing change, but also different levels of forcing change and different forcing agents. Therefore, it is not actually clear that the disappearance of surface isopyncals > $\sigma_2$max that explains the collapse of ΨNADW in RCP4.5/8.5 is purely a "rate-dependent" effect, but rather could be related to differences in the magnitude or structure of the forcing.
10) Conventional thought is that net freshwater fluxes in the North Atlantic should increase under global warming, primarily due to an enhancement of the hydrological cycle that would amplifying the global pattern of P-E (e.g., . Why is there a decrease in FW fluxes in this experiment?
11) Lines 221-226: The authors don't show or discuss similar watermass transformation for the RCP scenarios (aside from the densities shown in figure S1), so I feel that the claim that their result shows clear "indications of rate-induced effects" is not justified. For example, the RCPs may have completely qualitatively different evolutions of Bflux (not just different timescales of changes in Bflux) that give rise to greater AMOC weakening at a lower GMST: this would then undermine the assertion that "rate-induced" effects are the primary reason for differing AMOC responses. (This is related to general comment #2)
12) Line 255--256: Again, I don't think the claim that "AMOC tipping under anthropogenic climate change is primarily controlled by the warming rate" is fully supported by the evidence in this manuscript. It is clearly influenced by the warming rate, but, given that the different RCPs also have different forcing magnitudes and forcing agents, and that the AMOC in RCP8.5 collapses at a higher GMST threshold than in RCP4.5, one could just as easily conclude that AMOC weakening is governed by the spatial structure and temporal evolution of surface forcings, which are influenced by all of the above factors (not just the warming rate).
13) Paragraph starting at Line 292: I'm a bit confused about what the authors are trying to say here about salinity changes at 34S, mostly because the figures seem to contradict each other. In Figure 4d, the near-surface salinity at 34S seems to be decreasing, and the authors point this out in Line 292 and mention that it is surprising. However, Figure 5a shows a clear surface salinity signal at 34S, and the authors say that the Atlantic basin is salinifying, which seems to contradict Figure 4d? Moreover, the authors try to connect Atlantic salinity changes to changes in other basins (Pacific basin, Indian Ocean via Indian throughflow and Aghulas Leakage), but these explanations are purely speculative, as the authors do not perform any analysis that shows interbasin freshwater pathways, either oceanic or atmospheric (this would be quite challenging to do). I recommend the authors remove this speculative text and instead focus on the local changes occurring at 34S that can explain the changes in Fov.
14) Lines 325—229: The salinifying NADW in the CO_2 and in the low-intermediate RCP scenarios for CESM and CMIP6 models has already been discussed in lines 257—291, so it is not clear why the authors repeat this point here. Since the authors are analyzing surface salinity and freshwater fluxes in this section, perhaps it would be clearer to simply make the point that Figure 5a and b show a strong salinification over the WMT regions in the North Atlantic where NADW is produced, which gives a plausible cause for the salinification of the NADW mass at 34 S that was commented on previously.
15) Lines 331—334: "This also means that the destabilizing effects of anthropogenic climate change at high enough forcing rate can become larger than the stabilizing responses, such that the AMOC is brought out of equilibrium and subsequently collapses through a rate-induced AMOC tipping event." I don't believe this statement has been proven based on analysis presented in this paper, or at least, the authors have not made a convincing argument that their analyses show this (see major comment #3).
16) Line 428—430 (and elsewhere): the reduced sea ice extent not only leads to reduced insulation, but (perhaps even more importantly) to reduced freshwater—in fact this is the biggest contribution to the FW flux over the North Atlantic, where it can influence NADW production.
17) Line 444—445: "… we propose to convert the AMOC tipping warming threshold to a warming rate threshold" Again, I do not feel like the analysis presented in this paper justifies this: first, because the authors did not explore the impact of the rate of forcing change systematically, but rather that some mechanisms of AMOC stabilization/de-stabilization are fundamentally different in different forcing scenarios (of different forcing rates, forcing levels, and forcing agents), and second, because the experiments they did analyze don't reveal a straightforward relationship between AMOC collapse and the rate of warming: the AMOC collapsed at a lower GMST for the RCP scenarios that the slow CO_2 ramp, but actually collapsed at a lower GMST for the RCP4.5 vs 8.5 (despite it being a slower forcing rate of change). I think it is safer (and still very impactful!) to say that a pure GMST threshold for AMOC tipping clearly is not meaningful, but that other physics-based indicators may still be useful, but should also be re-evaluated in the context of different forcing rates and scenarios.

References:
Bonan, David B., et al. "Transient and equilibrium responses of the Atlantic overturning circulation to warming in coupled climate models: The role of temperature and salinity." Journal of Climate 35.15 (2022): 5173-5193.
P Nobre, et al., Amoc decline and recovery in a warmer climate. Sci. Reports 13, 15928 (2023).
Curtis, Paul Edwin, and Alexey V. Fedorov. "Collapse and slow recovery of the Atlantic Meridional Overturning Circulation (AMOC) under abrupt greenhouse gas forcing." Climate Dynamics 62.7 (2024): 5949-5970.
Trenberth, Kevin E. "Changes in precipitation with climate change." Climate research 47.1/2 (2011): 123-138.
Hankel, Camille. "The effect of CO2 ramping rate on the transient weakening of the Atlantic Meridional Overturning Circulation." Proceedings of the National Academy of Sciences 122.1 (2025): e2411357121.

Version 1:

Reviewer comments:

Reviewer #1

(Comments for the Author)
Review of van Westen et al, "Failure to track a stable AMOC state under rapid climate change"

Apologies for the delayed review. I have been traveling a lot and did not have the chance to get to it earlier.

This is a second review of the manuscript. The authors tried to address my comments and reservations, and I am grateful for this. I believe that the main message of the paper is clear and important to be made, namely that the rate of increasing emissions plays a role in determining the threshold for tipping behavior in the AMOC.

The paper is still too long I think but very worth publishing anyway. I have a few suggestions, which are minor in effort, but important to bring out the message. So please address them if you agree:

- Consider rephrasing the text in your abstract to highlight the important result that there is no single GWL (or CO2) threshold for AMOC collapse because these thresholds are pathway (rate) dependent.
- Likewise, note (as you already say in the text) that the term “tipping point” should be avoided and use “tipping transitions” instead. This should be in the abstract or conclusions too.
- Please note in your text that Hankel et al used the same model (CESM1).
- Also, it would be helpful to use warming rates rather than CO2 rates and GWLs in your abstract. For example, you say that the stable AMOC is found in the slow CO2 ramp up case. But what is the warming rate in this run? What is the warming rate in the fast CO2 ramp-up? You can provide average warming rates, I think that would be ok.
- In the table S1 (thank you for including it) could you please list the number of years of imposed forcing (FH), not the model year. I am not sure the model year is informative. Also in a separate column please list an “average” warming rate associated with the CO2rate. Ideally you would associate it with emissions rate too, but I understand that your simulations are not emissions-driven.
- Lastly you might want to highlight in your results that there is also no “system threshold” for AMOC collapse (i.e. a single AMOC strength level after which AMOC is considered collapsed – eg. Jackson et al take 6Sv as such threshold etc). $\psi NADW=0$ is a better system-threshold to assess collapse.

Reviewer #2

(Comments for the Author)
The authors have addressed my concerns well. I think the work can be accepted for publication.

Reviewer #3

(Comments for the Author)
Summary: The authors have made significant improvements to this manuscript, and I believe their main conclusions are now much better supported by their analyses. In particular, I appreciate that the authors were able to perform additional simulations with different CO2 increase rates, and I believe that including these results (as well as their more complete analysis of the RCP simulations) alongside the original simulations now convincingly shows that the mechanisms they present are indeed the ones leading to a rate-induced AMOC collapse. In addition, their re-writing of some sections (particularly on the freshwater budget of the Atlantic) has made the manuscript clearer and the logic easier to follow. Their response to my previous comments and re-writing of the Dynamical Perspective section has also strengthened my understanding of the connection between the box model and the CESM results. There are still some areas of the manuscript where the writing requires some clarification, which I provide suggested improvements for in my comments below, but overall, I think this paper should be published.
Comments:
1) In the “Stable AMOC” section, I suggest mentioning immediately (within the first paragraph, before getting into the details of the simulations) that the motivation for branching from the hosed PI state is to study an AMOC that is in a bi-stable regime (and thus, perhaps, more resembling the Earth’s AMOC). As of now, this reasoning is somewhat buried deep in the third paragraph, but it is very important for orienting the reader to the experimental design.
2) Calculation of ψ_{NADW} and related contributions: Is ψ_{NADW} calculated as the total surface WMT for all density classes greater than σ_{max}^2 (as lines 193/194 suggest), or is it simply the value of ψ_{surf} at σ_{max}^2 (as the notation $\psi_{NADW}= \psi_{surf} (\sigma_{max}^2)$ in line 197 suggests), i.e., essentially just looking at the maximum value of ψ_{surf}? I think, based on the following the discussion, that it is the former, but I recommend adding/standardizing the notation in lines 193—198 to make this more explicit—as it written now, it’s not clear that the “water supply of the North Atlantic Deep Water (NADW)” is the quantity ψ_{NADW}, and I found it confusing that the equation $\psi_{NADW}= \psi_{surf} (\sigma_{max}^2)$ drops the the $\sigma^2>\sigma_{max}^2$.
3) Assuming ψ_{NADW} is indeed calculated for all density classes greater than σ_{max}^2, then it is influenced by two factors: the area of $\sigma>\sigma_{max}^2$, and the changing Bfluxes. The former can, in turn, be further broken down into changes in surface isopycnal outcropping (σ^2) and changes related to interior ocean density (σ_{max}^2). By the end of the section, I was able to understand the relative contributions of these three factors, but I found that the way the authors phrased some things was a little bit confusing, and it may help if they try to streamline/re-order lines 202-224 to reflect this very intuitive breakdown. For example, this breakdown is sort of muddled when the authors say: “this combined response [that ψ_{NADW} remains large while σ_{max}^2 decreases] means that both the interior and surface sinking region are getting lighter at the same rate…”. In fact, that ψ_{NADW} remains large (actually increases) while σ_{max}^2 decreases could be from either of the two other factors: that surface densities in the sinking region are also getting lighter, OR that Bfluxes are getting more negative. Later, they show that indeed the ψ_{NADW} changes are because of both these things, so I think this sentence is somewhat misleading. I suggest the authors try reorganizing these few paragraphs so that the logic is more coherent. For example, rather start with focusing on σ_{max}^2, they could start by describing that $\sigma^2>\sigma_{max}^2$ (the quantity that goes into the calculate of ψ_{NADW}) actually increases, and then reveal that this increase can be attributed to the fact that while surface

densities are getting lighter in the sinking region, σ_max^2 is also getting lighter at a comparable rate (but this would not be expected under fast forcing changes).
4) Paragraph starting on line 246: the role of new deep convection areas developing due to sea ice loss is quite interesting, but I would not necessarily expect this to be a rate-dependent effect (since presumably all experiments lose their Lab. Sea ice). Does this mechanism occur in the faster ramping/RCP scenarios, and if not, why?
5) Line 298-299: I'm a bit confused by the sentence "The surface flux Fsurf is slightly declining prior to the strong increase in Fov, which implies that the salt-advection feedback is triggered by heat fluxes." How does this conclusion follow? Are the authors trying to say that surface freshwater fluxes cannot trigger the initial AMOC decline because they are going in the wrong direction?
6) Figure 5 caption: It would be good to add early in the caption that these subfigures are all for the gradual CO2 ramp experiment.
7) Line 300: "dominant"—dominant compared to/over what? Do you just mean it's large compared to the slow CO2 ramp?
8) Line 337: What is "the latter" referring to?
9) Discussion of NADW salinification: Starting around line 348, the writing here makes the connection between analyzing FovS and NADW a bit unclear (in particular, I don't understand the phrase "allowing the interhemispheric salinity contrast to adjust"). Rather than claiming that increased NADW salinity is a "fingerprint" of increased AMOC stability, it might be clearer to say that you're analyzing the main contributions to the increasing FovS (which the authors have already established can be understood as an indicator of AMOC stability). The main contributions could be freshening of the upper ocean at 34S or salinification of the NADW—the former is less consistent across models, so you conclude that salinifying NADW is a main cause of a stabilizing climate, and is expected from increased net evaporation in the North Atlantic. Relatedly, it seems important to say somewhere in this paragraph (and later when concluding the section too, see point #11) that this stabilizing process can only occur under sufficiently slow forcing changes, since it requires GMST to adjust and for the salinification signal to propagate all the way to 34S at depth. This is the key intuition for why you see this signal under slower forcing scenarios, but I'm not sure if you say it anywhere explicitly.
10) Lines 356—360: I found the discussion of +2.5C as a sort of threshold that delineates between experiments' NADW behavior to be odd, as there's no physical reason that the salinification of NADW should exist/not exist above/below this threshold. Rather, since P-E changes should scale somewhat linearly with GMST, wouldn't you expect a smooth range in the strength of the NADW salinification signal that scales with the range of GMST changes?
11) Related to point #9 above: Overall, this section is a lot easier to follow than it was in the first draft, but I still found it very long and challenging, and by the end, it was not entirely clear what the primary mechanism related to the particular analyses of this section was that could help explain the rate-dependent AMOC collapse. I would encourage the authors to consider finding paragraphs to shorten/remove, and also to conclude this section by summarizing/highlighting which aspects of the freshwater budget mechanisms clearly depend on the forcing rate. For example, they say rather vague summary statements like "the rate-dependent destabilizing effects of anthropogenic forcing must overcome the slow stabilizing effects", but what are those stabilizing/destabilizing effects, and why do they depend on the forcing rate (again, specific to the analysis in this section)? I think the main answer to this is that surface salinification of the (north) Atlantic can stabilize the AMOC by increasing FovS, but this occurs on a slow timescale because it requires (a) GMST to adjust to radiative forcing (in order to enhance net evaporation) and (b) the surface salinity signal to propagate downwards in the North Atlantic and salinify the outgoing NADW at 34S. Under fast radiative forcing changes, net surface heat fluxes weaken the AMOC and kick off the salt-advective feedback before this stabilizing process can happen (and, net freshwater fluxes are not counteracting this as much as they are in the slow ramp). Hopefully the authors can highlight and clarify this at the end of the section, because as it stands, the rate-dependent nature of this stabilizing feedback is not made very explicit.
12) The Dynamical Perspective is much more effectively written now, and I thank the authors for clarifying that the effective precipitation depends on the North Atlantic ocean temperature, not the imposed atmospheric temperature (I misunderstood this previously).

Open Access This Peer Review File is licensed under a Creative Commons Attribution 4.0 International License, which permits use, sharing, adaptation, distribution and reproduction in any medium or format, as long as you give appropriate credit to the original author(s) and the source, provide a link to the Creative Commons license, and indicate if changes were made.
In cases where reviewers are anonymous, credit should be given to 'Anonymous Referee' and the source.
The images or other third party material in this Peer Review File are included in the article's Creative Commons license, unless indicated otherwise in a credit line to the material. If material is not included in the article's Creative Commons license and your intended use is not permitted by statutory regulation or exceeds the permitted use, you will need to obtain permission directly from the copyright holder.
To view a copy of this license, visit https://creativecommons.org/licenses/by/4.0/

MS-No.: NCLIM-26020620

Version: Revision

Title: Failure to track a stable AMOC state under rapid climate change

Author(s): René M. van Westen, Reyk Börner and Henk A. Dijkstra

Point-by-point reply to reviewers

April 28, 2026

We thank Anastasia Romanou and two anonymous reviewers for their careful reading and for the useful comments on the manuscript.

Reviewer #1

This is a review of the manuscript entitled "Failure to track a stable AMOC state under rapid climate change" by van Westen, Börner and Dijkstra. The paper discusses AMOC evolution under different rates of warming and constant freshwater forcing and the timing of crossing critical thresholds in one model mainly but compares to other models as well.

The paper includes some very important & original results and should be published, however there are some necessary adjustments, I would not call them minor or major, but just necessary. The conclusions are robust but some questions exist.

The main issue is that the paper is not very clearly written which is unusual for these authors. At some places it is a lot more detailed and in other places too brief. It would be straight forward to go through it and just tighten up the text. Additionally, a table with crossing times, emissions or concentration levels and warming levels would help summarize the results better, particularly if it includes information about type of forcing (idealized, CO2 only, RCP forcing etc). The use of the box model as is now is not necessary, otherwise it needs to be expanded.

Thank you for your positive comments on the manuscript. We have performed additional CESM simulations with faster CO_2 rates, below referred

to as CO_2 ramps, and these new results helped to improve the manuscript. The results and conclusions remain robust and further strengthen our claim of rate-induced tipping. The manuscript text has been fully revised, taking your comments into account.

The abstract (and possibly the title) do not convey clearly the points of the paper. In my opinion, and the authors may disagree and it is their decision to make, the abstract should cast more emphasis in the rate dependency of the "realistic" scenarios and less so in the slow (highly idealized) experiment. The last sentence of the abstract is key. There will be more papers around this theme.

We kept the title the same but changed the abstract, also in light of the additional faster CO_2 ramps. Note that our main aim is to demponstrate the rate dependence of AMOC tipping in itself (i.e. whether the AMOC tracks its current stable state or tips to an alternative state) – less so the rate dependence of the timing of tipping onset under "realistic" ramping rates.

Other comments:

1. *Lines 60-62: AMOC destabilization is not just through the larger scale salt-advection feedback. Noise induced instability has been shown in models as well (e.g. Romanou et al. 2023; Oh et al. 2025). Local feedbacks and processes may disrupt the operation of the large-scale salt-advection feedback and lead to abrupt shifts in AMOC. This is why several models with a negative salt-advection feedback, get AMOC collapse.*

   We do acknowledge the role of noise-induced instability, which can eventually trigger the destabilising salt-advection feedback. This sentence was rewritten and we included the relevant references.

2. *Lines 63-65: this is only evidence from CESM1 while other models show that AMOC can collapse for smaller rates of freshening, sometimes Greenland ice sheet melt is not necessary (e.g. Romanou et al. 2023)*

   The reviewer is correct here. We have rewritten this entire section.

3. *Lines 72-73: the baseline simulation (reported in van Westen et al 2024; Science Advances) takes about 800 years to collapse under a flux which*

is 0.03Sv/yr. This is a huge freshwater forcing -not really supported by current climate evidence. Also, freshwater flux was applied over a large area of the North Atlantic 20-50N. Here AMOC collapse starts after 1700 years. These points need to be made clearly in the text (in the Discussion or here) to aid readers parse the importance of the freshwater forcing in different models.

Please do note that the AMOC collapse takes about 100 years in the quasi-equilibrium PI hosing (Figure 1a), while the full equilibration to the collapsed state takes an additional 300 years. Nevertheless, we agree with the reviewer that these freshwater flux forcings need to be placed into context. This is now addressed when introducing the quasi-equilibrium PI hosing (section 'Stable AMOC under extreme climate change').

4. *Lines 84-86: Freshwater compensation to conserve ocean salinity. Although it is general practice it is incorrect and needs to be shown that adding salt (increasing mixing) elsewhere does not affect currents and transports. The practice is invoked because many models, including CESM1, have rig-lid approximations and cannot add a physical freshwater flux in the model and must resort to adding or subtracting salt. However, adding/subtracting so much salt over prolonged periods of time has implications for local stratification profiles, mixing etc. This is a moot point however, since the authors cannot do anything about it. But I would welcome a very brief discussion on the importance of the salt correction.*

   This is indeed the standard protocol and we do note that the local hosing compensation is typically a factor 10 smaller than the local freshwater fluxes. However, it is good to comment on the hosing location and compensation procedure and this has been added in the revision.

5. *0.5Sv over 1500 yrs and over 20-50N. This is a very large freshwater flux for a very large region in the domain. In the discussion it would be nice to compare it to SMB changes for the Greenland ice sheet.*

   The forcing of 0.5 Sv is about 65× the present-day Greenland Ice Sheet melt. As was argued in previous work, this is unrealistically large and related to persistent climate model biases; this is now mentioned in the revision.

6. *Lines 98-100: the historical and future CESM runs have additional freshwater forcing. I would like to see in the Supplement a figure with comparison of the total forcing in these runs.*

   Full details of these RCP simulations were presented in van Westen et al., 2025, https://doi.org/10.1029/2025JC022651). Revised Figure S2h shows a key summary of the total North Atlantic forcing (B_{flux} decomposition).

7. *Fig. 1c: the warming in this simulation seems too high. For example at 400 years, CO2 atm concentrations are 284+0.5ppm/yr*400=484ppm and the temperature change is about 1.8deg! And you add freshwater but the AMOC declines very little. Why? Some clear quantification of these numbers would help (preferably in a table?)*

   These relatively high GMST anomalies in CO_2 space are correct and are related to slow climate feedbacks that contribute to additional warming. We have added faster CO_2 ramps, suggested by Reviewer #3, and they have lower GMST anomalies in CO_2 space. We comment on the warming levels in the revision.

8. *Fig. 1d: The sensitivity to freshwater forcing in the slow ramp-up (PI CO2) is stronger than due to CO2 forcing in the historical. That is probably because all these runs are branched off an already destabilized AMOC. Is this a fair comparison though? Or maybe I am missing something -apologies I was a little lost as to what the forcings in each run are. Also, how do you define the "tipping" threshold in the RCP8.5 being 2.8 degrees? There is tipping in RCP4.5 at 2.2C but I do not see tipping in RCP8.5 at 2.8C which is contrary to what you are trying to convey. There is steady weakening in AMOC but not tipping. Please clarify. Therefore I do not agree with lines 127-128.*

   We have streamlined this section and included a table and a description of the onset of an AMOC collapse (see revised Methods).

9. *Lines 150-155: what is the contribution of the wind driven component of the AMOC?*

   The wind-driven component to deep water formation is relatively small compared to the surface buoyancy fluxes. Adding these details will distract from the overall aim of this manscript; no changes in the manuscript were made here.

10. *Figure S1a: why is there (substantial!) convection area increase in the ramp-up case? (small inlet figure). I would expect freshening. Where does this freshwater go? Mixes down too fast (relative to the forcing) or is advected away too fast? Or is salt advected in the region (due to salt correction)?*

    The sinking area is only increasing over the first 800 model years and is related to the retreating sea-ice cover; this is now mentioned in the revision. As was discussed in the manuscript, the retreating sea-ice cover causes a negative freshwater flux forcing instead.

11. *Line 194: how important is the area of the calculations 40-65N? Surely, this area is much broader than the convection sites. I know there is justification for this in earlier papers, but please repeat it here too.*

    The results are not sensitive when slightly varying this latitude band, which is now mentioned in the revised Methods and referring to van Westen et al. (2025, https://doi.org/10.1029/2025JC022651).

12. *Line 218-220. It is interesting that AMOC strengthens and MLDs deepen (Fig. S3d) while there is continuous warming. There is clearly a shift here but if it is not relevant to the topic of the paper, there is no need to discuss. In any case, Labrador Sea mixing is shutdown from the start of the simulation (sea ice covered) but then resumes presumably due to warming. Even so, the mixing is too shallow to feed in AMOC.*

    The abrupt AMOC increase (found in many AMOC-related time series) and the role of reduced sea-ice melt is now made more explicit by analysing the freshwater budget between 40°N to 65°N (revised Figure 4).

13. *It is great that you point out in Line 235 that FovS is only applicable as a criterion for AMOC stability under equilibrium, however, it is used broadly by the community as an indicator even under transient warming. Perhaps you want to highlight this point a little more.*

    Thank you. We have completely streamlined this section and emphasised the role of F_{ovS}.

14. *Line 253: important finding: there is no threshold but rather behavior is controlled by the warming rate. However, I would rephrase this that the critical threshold is rate dependent. If there is tipping behavior,*

there is a critical threshold. It is just not universal, and I agree is not a certain GMST. Other studies under review right now are showing the same result.

Agreed, suggestion followed. This critical threshold is explored with the conceptual (box) model (see also our answer to comment #17 below). Note, however, that there can be tipping events without crossing a critical forcing threshold (e.g. noise- and rate-induced tipping). In this case, the critical threshold is a threshold in state space, which may or may not depend on the forcing.

15. *Lines 282-285 and later: unclear what the conclusion is here from the multi-model comparison.*

    Our results may be sensitive to the model used (CESM) and its specific equilibrium chosen ($\overline{F_H} = 0.45$ Sv). Therefore, we explore whether the stabilising AMOC response also holds for different CMIP6 models. The salinifying NADW is an important fingerprint of increased AMOC stability. We have streamlined this section to clarify the conclusion from the multi-model comparison.

16. *Figure 5: it seems to me that there is a strong shift to the south of the ITCZ even though there is no AMOC weakening in the ramp-up run. Can you explain the patterns? And the surface salinity increases significantly, consistent with AMOC strengthening. I am not sure what is going on here. Is the ramp up too slow and the adjustments too fast?*

    ITCZ shifts are possible under global warming alone (see Figure S8 in van Westen et al. (2025, https://doi.org/10.1029/2025JC022651)), which is also the case for slow CO_2 ramp. However, including this ITCZ analysis distracts from the overall aim of the manuscript and Figure 5 was moved to the supplementary material (now revised Figure S5). We also reduced the text on this figure to improve readability.

17. *The use of the box model is unclear to me. Please explain what simulations you are fitting it to. If they are not the same simulations as the ones you used before, then I do not see what this section adds to the paper. This section would actually merit its own paper, I think.*

    We believe that the idealised box model is useful for (a) illustrating the distinction between bifurcation and rate-induced tipping in an AMOC-relevant modeling setup, (b) building an understanding of the role of

the saddle-node bifurcation under radiative forcing pathways and (c) establishing a dynamical perspective on AMOC tipping under transient climate change that incorporates the combined and interdependent effects of heat and freshwater surface forcing. We have further clarified the motivation for using the box model and strengthened its connection to the CESM results by redesigning Figure 6.

Minor comments:

1. *Line 40: distinguish studies discussed here. Mechanism is different in 13-15 references. Romanou study is about 2xCO2 and warming levels are exactly the same as pointed out here.*

   We have removed the discussion of the Romanou study here, as the comparison is difficult to make due to other forcing agents (CO_2, methane, aerosols). This has been further discussed when introduction the RCP scenarios (as was suggested by Reviewer #3).

2. *Figure 1 titles are corrupted. Please go over the captions and figure legends closely.*

   Corrected.

Reviewer #2

This study uses CESM simulations to demonstrate that the stability of the Atlantic Meridional Overturning Circulation (AMOC) is critically dependent on the rate of radiative forcing, not just the final level of global warming. Under a very slow CO2 ramp-up (0.5 ppm/yr), the AMOC remains stable up to +5.5°C of warming. In contrast, under the faster RCP4.5 and RCP8.5 scenarios, the AMOC collapses at much lower warming levels (+2.2°C and +2.8°C). The authors identify a stabilizing mechanism involving enhanced evaporation, reduced sea-ice extent, and a salinifying North Atlantic Deep Water (NADW), which allows the AMOC to track its stable state under slow forcing. An idealized box model is used to provide a dynamical perspective on this rate-dependent tipping. While rate-dependent effects have been discussed in conceptual models (e.g., Stocker & Schmittner, 1997), this study provides a clear demonstration in a comprehensive, state-of-the-art Earth System Model (CESM). The core finding that a warming threshold is not a robust predictor of AMOC collapse and that the forcing rate is the critical control parameter challenges a widely held paradigm. The result that the AMOC can remain stable at +5.5°C under slow forcing, while collapsing at +2.2°C under faster forcing, is striking and has profound implications for how we assess and communicate climate tipping risks. This work effectively shifts the focus from "how much warming is too much?" to "how fast is too fast?". The methodology is sound and appropriate for the research question. The conclusions are well-supported by the model analysis. The paper is well-structured and the narrative flows logically from results to discussion. However, some sections could be streamlined to better serve the main argument (see specific comments below). I recommend publication after minor revisions.

Thank you for your positive assessment on the manuscript, please find our specific answers below.

1. *A key question arising from this single-model study is the generalizability of its quantitative findings. As the authors note, the experiment is initialized from a specific hosed equilibrium (0.45 Sv), which places the AMOC in a particularly sensitive state. The robustness of the critical warming rate, and even the detailed evolution of the AMOC (e.g., the activation of Labrador Sea convection around year 800), would likely*

be sensitive to the choice of model and its parameterization schemes, particularly those for sea ice and deep convection. Models with different representations of deep convection may not exhibit the same sharp AMOC strengthening upon sea-ice retreat. The authors' use of an idealized box model (Figure 6) is a valuable step towards demonstrating the broader validity of the rate-dependent mechanism. However, a question remains: Can the warming eventually overcome the stabilizing effects of salinification under any forcing rate, or does the existence of a slow enough pathway imply an absolute limit to AMOC resilience?

We agree with the reviewer that our quantitative findings (e.g., critical forcing rate or sea-ice feedbacks) may depend on the model used (CESM) and on the specific background state ($\overline{F_H} = 0.45$ Sv) chosen. The sensitivity to the hosing background state is illustrated by the RCP4.5 scenarios, in which the AMOC either recovers ($\overline{F_H} = 0.18$ Sv) or collapses ($\overline{F_H} = 0.45$ Sv). To further address these model and equilibrium sensitivities, we have included analyses based on CMIP6 and idealised models, which show responses that are consistent and robust with the CESM.

We expect that the Atlantic basin will continue to lose freshwater through its surface beyond +5.5°C of warming. This modified Atlantic freshwater budget results in a weaker salt-advection feedback strength, associated with a lower negative magnitude of F_{ovS}. If F_{ovS} were to become positive, indicating that the salt-advection feedback is stabilising the AMOC, it would be very unlikely for an AMOC tipping event to occur. Hence, we expect the model to track stable AMOC states under very slow warming rates. Only when the stabilising feedback becomes sufficiently weak, as demonstrated by the critical rate analysis in the idealised box model, does the warming dominate and lead to AMOC collapse.

We discuss the Atlantic freshwater budget and the role of F_{ovS} in greater detail in the revised paper.

2. *Baker et al. (2025) present a compelling, global-scale mechanism for AMOC resilience, arguing that persistent Southern Ocean winds sustain a weakened, but not collapsed, AMOC by driving a Pacific Meridional Overturning Circulation (PMOC). Their findings provide a fascinating complement to this study. Baker et al. emphasize a global, mechanical*

constraint (Southern Ocean winds and the necessity of a PMOC for a full collapse), while van Westen et al. focus on regional, buoyancy-driven processes in the North Atlantic that allow the AMOC to track its stable state. These perspectives may not be contradictory but operate on different scales. A collapsed AMOC (as per Baker et al.) is a global state requiring a PMOC. Van Westen et al. show that even under extreme warming, the AMOC avoids this global collapse if the forcing is slow enough for the North Atlantic to adjust. I am wondering how the rate-dependent buoyancy adjustments the authors identify might influence the likelihood and strength of PMOC emergence, thereby linking their regional mechanism to the global-scale constraints highlighted by Baker et al. Does a faster forcing rate, by maintaining a weaker NADW outflow, contribute to the development of a PMOC?

Whether a PMOC forms after substantial AMOC weakening (or collapse) is not central in this manuscript as we focus on tracking a stable AMOC state under climate change. A potential PMOC contribution was noted in van Westen et al., (2025, https://doi.org/10.1029/2025JC022651) for the RCP4.5 and RCP8.5 scenarios, as the AMOC does not reduce to 0 Sv ($\overline{F_H} = 0.45$ Sv, Figure 1b) while under constant PI conditions a collapsed AMOC state of 0 Sv does exist (blue markers in Figure 1a). As it diverges from the main issue addressed in this paper, we decided not to include a PMOC analysis in the present paper, but leave this for future work.

3. *The study might benefit from a more explicit connection to the work of Dai et al. (2025), who propose subpolar North Atlantic sea surface salinity (SPNA SSS) as an AMOC indicator. While the study show a salinifying NADW under slow warming (implying stability), Dai et al. argue that a significant freshening (e.g., 2 PSU) would be needed for a substantial AMOC slowdown. This apparent contrast highlights the rate-dependent nature of the problem: a slow freshening might allow for compensating mechanisms (such as those identified here), while a rapid freshening could lead to collapse. The authors should discuss how their rate-dependent framework could reconcile these different perspectives. Specifically, is the concept of a 'critical freshwater volume' for collapse itself a function of the forcing rate? A slow, steady freshwater input might be balanced by the stabilizing mechanisms described (enhanced evaporation, NADW salinification), whereas the same total volume de-*

livered rapidly could overwhelm the system and trigger a collapse. This would be a valuable extension of the freshwater hysteresis framework into the rate-dependent regime.

We agree that such an analysis is a valuable addition to the manuscript. Revised Figure 4 now shows the freshwater convergence and freshwater content between 40°N – 65°N, where large-scale freshening over the upper ocean is key for a destabilising AMOC, consistent with Dai et al. (2025). We have rewritten this section and incorporated the study of Dai et al. (2025) here.

Reviewer #3

This manuscript examines the evolution of the AMOC in a new climate modeling experiment in which atmospheric CO2 is increased very gradually over a few millennia and compares it to the AMOC evolution in the same model under more common Representative Concentration Pathways (which have a variety of levels and rates of greenhouse gas increase). The biggest takeaway from the comparison of these different experiments is that strong AMOC state can be maintained under very high levels of global mean warming when the rate of CO2 change is very slow, while the AMOC would collapse at a much lower level of global mean warming under faster and more extreme forcing scenarios. This has the implication that global mean surface temperature "thresholds" for an AMOC tipping point are not meaningful, which changes how we think about the usefulness of policies aimed at limiting global warming to a certain level. The authors then perform two major categories of analysis to try to identify the physical mechanisms that stabilize the AMOC when the forcing rate is slow but fail when it is fast. Finally, they illustrate that in a simple conceptual model of the AMOC forced with temperature and freshwater changes, the collapse of the AMOC can be induced by faster rates of warming.

Overall, I think the main point of this paper (that a true global warming threshold does not exist for an AMOC tipping point because the tipping point is forcing scenario-dependent) is impactful and novel, both for the scientific field and the general public. However, I do not believe the authors' secondary claim that the forcing rate is the primary determinant of whether a tipping point is crossed is fully justified, and significant work is needed to make the analysis more complete, logically sound, and supportive of their conclusions. In particular, because the study compares a slow CO2 ramping rate to RCP scenarios in which not only the forcing rate, but also the forcing magnitude and types of forcing agents are different, the authors do not convincingly show that the differing AMOC evolutions are actually attributable to the different forcing rates rather than these other factors. In some sections (which I elaborate on below), they identify mechanisms that stabilize the AMOC in their slow CO2 ramp, but do not show or explain why such mechanisms would not stabilize the AMOC under faster forcing rates, thus failing to connect their mechanistic insights to the forcing rate of change itself (which is central to

their argument). Finally, I do not think the "dynamical perspective" section that uses the AMOC box model provides much scientific insight as it currently stands, because the mechanisms of rate-induced AMOC collapse in the conceptual model are not the same as those in the Earth System Model, so I recommend the authors either remove this section, or think carefully about how to re-formulate the box model to be more relevant for interpreting the CESM results. If the authors can resolve these issues, I believe the mechanistic explanations for the differing AMOC behaviors in the different modeling experiments have the potential to significantly deepen our understanding of rate-sensitivity of the AMOC and whether such rate sensitivity is enough to cause a tipping point. Below, I provide general comments for each of the three results sections, and then more specific comments which may also help resolve the general comments.

Thank you for the positive assessment of the manuscript.

General comments:

1. *Isolating the role of forcing rate: As mentioned above, one of the major ways that the authors' analysis does not necessarily support their conclusions is that the experiments they compare (the CO2 ramp, the RCPs in CESM, and the SSPs in other CMIP models) have not only different forcing rates, but also different forcing magnitudes, forcing agents, and even declining forcing (in the case of RCP2.6), so the differing mechanisms of AMOC stabilization or collapse that they explore may not necessarily be due to different forcing rates. In other words, they do not convincingly isolate the effect of the rate of forcing increase, despite this being central to their main conclusions. There are a few ways they could overcome this. One would be to run an additional experiment in CESM where CO2 is ramped to the same final value as in their current experiment, but at a much faster ramping rate (perhaps comparable to the rate of increase in an intermediate or high RCP scenario). The authors could then focus the bulk of their analysis on comparing the two CO2 ramps, but insofar as they identify similar features in the "fast" ramp as in the RCP and SSP scenarios, this would strengthen the claim that the collapse of AMOC in intermediate and high future emissions scenarios across CMIP models is driving by the failure of the AMOC to track its otherwise stable steady state. This would also eliminate the*

possibility that other forcing agents in the RCP/SSP scenarios are playing a role (for example, Northern Hemisphere aerosol changes, which may be relevant for surface buoyancy fluxes over the North Atlantic). To me this would be the most elegant solution, and is the one I recommend, but I recognize it requires an additional modeling experiment with additional computational resources. Another option would be to analyze segments of the CO2 ramp and RCP and SSP scenarios that have similar levels of CO2 change, but different rates of change (for example, the first 50 years of RCP8.5, 100 years of RCP4.5, and 600 years of the CO2 ramp, all up until CO2 reaches 580 ppm). Then if they identify that key stabilizing mechanisms found in the CO2 ramp are weaker or overcompensated by other mechanisms in the "faster" forcing scenarios (and can explain why this is related to the rate of forcing change), then I believe they would have a convincing argument that the eventual AMOC collapse in the higher RCP scenarios is due to forcing-rate-dependent stabilizing mechanisms. The third option (and this would be the bare minimum), is to clearly show that the mechanisms stabilizing the AMOC in the slow CO2 ramp do not operate in the RCP/SSP experiments for reasons directly related to the different forcing rate of change. To some extent they do this in their analysis of surface water mass transformation by showing that the AMOC collapse occurs in the high emissions scenarios because the surface densities change faster than interior densities (thus shutting down surface WMT), which is clearly related to the interior ocean being out of equilibrium under faster forcing scenarios (though some key analyses are missing here, as I describe next).

We agree with the reviewer that the comparison between our CO_2 ramp and RCP/SSP simulations is biased due to different forcing agents (e.g., aerosols). Hence, we followed the 'most elegant solution' proposed by the reviewer, and conducted two additional CO_2 ramp simulations: +2.5 ppm yr^{-1} (factor of 5 faster) and +5.0 ppm yr^{-1} (factor of 10 faster). For reference, the average CO_2 growth rate at the Mauna Loa Observatory is +2.60 ppm yr^{-1} (2015 – 2025).

The AMOC collapses for both the +2.5 ppm yr^{-1} and +5.0 ppm yr^{-1}, which fully supports our earlier claim of rate-induced tipping. These new model simulations are presented and discussed in the revision, and the relevant text and figures are changed accordingly. It is still useful

to report the results of the RCP simulations, where the RCP2.6 shows similarities with the slow CO_2 ramp and the RCP4.5 and RCP8.5 with the faster CO_2 ramps. When discussing the RCP/SSP results, we stress that other forcing agents are also important.

2. *Section on water mass transformation: I believe the results here do support the conclusion that the forcing rate influences whether the AMOC collapses or not, but some additional analyses and clarity are needed to make this evidence stronger. First, if the authors plan to use the factors contributing to* Ψ_{NADW} *to explain the differences in AMOC behavior across the CO2 ramp and RCP experiments, they need to show that* Ψ_{NADW} *at least qualitatively matches the AMOC behavior in all experiments analyzed, in particular that it captures the greater AMOC weakening in the RCPs. To do this, I recommend they plot* Ψ_{NADW} *for the RCPs, not just for the CO2 ramp. (This would also help justify their approach of looking at* Ψ_{NADW} *to diagnose AMOC changes, which at the moment seems rather dubious given that* Ψ_{NADW} *has a different trend than the AMOC index in the CO2 ramp–clearly positive, as opposed to near-zero). Then, they could break down* Ψ_{NADW} *changes into changes from Bflux and changes from the area of deep water production shrinking (in both the CO2 ramp and the RCP experiments), where I expect they will find that the key difference between the slow CO2 ramp and the fast RCPs is the shrinking of the area where surface densities are greater than* σ_2^{max}*, rather than differences in Bflux. After doing this, the authors can then use Figure S1 to confirm that the differences in* Ψ_{NADW} *across the experiments, which are correlated with and perhaps driving the differences in the AMOC strength, are due to forcing rate-dependent effects (lagged adjustment of interior ocean densities under faster forcing changes). By fully analyzing the surface water mass transformation in the RCPs in addition to the CO2 ramp, this series of analyses will conclusively show that the greater AMOC weakening in the RCPs can be traced back to the faster forcing rate leading to differing WMT evolution.*

   We agree with the reviewer that Figure S1 supports our key finding of rate-induced tipping and decided to move this figure to the main text, now as revised Figure 3 (Figure 2 was moved to the Supplementary Material, now revised Figure S1). We included the results of the faster CO_2 ramps in revised Figure 3, together with the time series of Ψ_{NADW}.

Near-zero values of Ψ_{NADW} indicate a closure of the adiabatic pathways, which happens before the AMOC reaches very weak strengths (< 5 Sv).

We also included the B_{flux} decomposition for the faster CO_2 ramps (revised Figure S2), which look similar to the AMOC tipping cases under RCP4.5 and RCP8.5. The RCP4.5 and RCP8.5 decomposition were presented in previous work (van Westen et al., 2025, https://doi.org/10.1029/2025JC022651).

3. *Section on freshwater budget: In this section the authors analyze changes in Fov,S in the slow CO2 ramp to identify another stabilizing feedback (salinification of returning NADW) that keeps the AMOC strong under slow forcing changes. However, their eventual claims that this stabilizing mechanism fails under faster forcing changes are not supported, as they do not analyze this mechanism for the high-RCP scenarios where the AMOC collapses. Specifically, the authors do not show Fov,S or NADW salinity changes (which they claim help explain the Fov,S trend) in the RCP8.5 scenario, and therefore do not provide any evidence that the stabilizing NADW mechanism is failing in RCP8.5, nor do they offer any clear reason why this failure would be caused by a faster forcing rate. To put it more directly: if the authors want to claim that the AMOC collapses in RCP8.5 but not in other scenarios because the higher rate of forcing change causes the stabilizing NADW mechanism to fail, they need to show a) that the NADW is not salinifying in RCP8.5 or that it is overcompensated by a different destabilization process and b) that whatever process they find in (a) is caused by the higher rate of forcing change. For example, could it be the case that NADW does not salinify in RCP8.5 runs because precipitation increases occur faster than evaporation increases (due to the slow adjustment of SSTs driving lagging behind the forcing under fast changes), leading to a net increase in the FW flux over the NADW formation regions? This is just an example of an analysis/explanation that would better support the authors' claim. The authors do a better job of establishing this logical chain in their discussion of WMT in the prior section, which they could model here: they show that NADW production cannot be sustained in the RCPs because the area of deep water formation (where surface outcroppings $> \sigma_2^{\max}$) disappears, and importantly they are able to show that this is a "rate-induced" effect. I recommend the authors try to establish a similar logical chain in this section. To*

do this, they need to perform similar analyses of Fov,S and its drivers in their experiments where the AMOC collapses, and show a forcing-rate-dependence of their proposed stabilizing mechanism. In fact, I do not understand why the authors only chose to analyze scenarios where the AMOC remained "sufficiently strong" (line 269) when their goal is to connect the collapse of the AMOC in high emissions scenarios to rate-dependent stabilizing mechanisms.

The use of F_{ovS} as a stability indicator is only appropriate under (quasi-)equilibrium conditions, which are not satisfied under RCP8.5. While F_{ovS} represents the inter-hemispheric meridional salinity gradient (Vanderborght et al., 2025, https://doi.org/10.1175/JCLI-D-24-0570.1), AMOC destabilisation takes place in the northern North Atlantic Ocean. To address this and following the reviewer's suggestion, we have included a freshwater budget analysis for the North Atlantic (40°N – 65°N), which clearly shows the destabilising salt-advection feedback for the faster CO_2 ramps (revised Figure 4). For the slow CO_2 ramp, the salt-advection feedback is not activated, which further motivates the analysis of the full Atlantic freshwater budget (now shown in revised Figure 5). Do note that a salinifying NADW is only a fingerprint of AMOC stabilisation, this is clarified in the revision.

In addressing the above, I think the authors could also simplify this section by only focusing on key analyses (but performing these analyses for all CESM experiments). Some analyses in this section were confusing, seemed to stray away from core AMOC-stability mechanisms, and might be extraneous. These included the discussion of freshwater convergence in the Atlantic, which is not as clearly tied to AMOC stability as Fov,S and the discussion of interbasin exchanges, which I mention below. I recommend focusing solely on Fov,S, identifying what causes changes in it (presumably salinifying NADW), and whether those changes can be tied to surface perturbations.

We included summary figures for all CESM experiments, for example, see revised Figures 3g,h.

4. *Dynamical perspective: In this section the authors use a conceptual box model of the AMOC to show that crossing an AMOC tipping point depends on the rate of warming when freshwater changes are coupled to temperature changes. Though this model is updated from a previ-*

ous iteration to include competing freshwater and temperature effects on the AMOC under anthropogenic climate change, it is not clear to me what new insights are generated from it with respect to interpreting the results in the rest of the manuscript. This is because the conceptual model (at least by my understanding) does not actually capture the key proposed mechanisms by which the AMOC remains stable under gradual warming in CESM: simultaneous adjustment of the interior and surface ocean densities that allows for continued surface WMT, and salinification of NADW due to enhanced basin-wide evaporation. Rather, the stabilization occurs simply by one forcing (freshwater) being prescribed to counteract the effects of another (northern temperature increase) on the AMOC. Moreover, that the freshwater forcing parameter EA is prescribed to decrease linearly with warming actually suppresses/goes against one of the most important rate-induced forcings on the AMOC: that surface freshwater fluxes over the North Atlantic tend to increase with warming on short/fast timescales, either due to enhanced P-E (e.g., Trenberth 2011) or sea ice meltwater changes (Hankel, 2025). Thus, it seems that the conceptual model more so serves to illustrate what a rate-induced tipping point is rather than capture the rate-dependent mechanisms governing the AMOC. If the physics of the conceptual model are actually more closely aligned with the AMOC-stabilizing mechanisms in CESM than I realize, then hopefully the authors can clarify this. Otherwise, I recommend that the authors only include this section in their revised manuscript if they can find a way for their model to capture the actual mechanisms of rate-sensitive AMOC stabilization found in CESM. If they can do so, then this would strengthen their claim that the AMOC behavior in CESM is actually a rate-induced tipping point, but if this cannot be done, then I think the manuscript already has offers significant insight without including this section (provided the authors resolve the other issues mentioned above).

The 5-box AMOC model captures the essential CESM dynamics, including the salt-advection and gyre feedbacks. Although water mass transformation rates are not represented in the box model, Vanderborght et al. (2025, https://doi.org/10.1175/JCLI-D-24-0570.1) demonstrated that AMOC collapse dynamics in the CESM can be captured in a low-dimensional model, supporting the use of these con-

ceptual (box) models. The box model also enables the assessment of asymptotic stability of the AMOC under climate change (see also specific comment #3 by the reviewer), which cannot be directly analysed with the CESM. However, we agree that the connection between the box model and the CESM should be strengthened. This has been addressed in the revision by including AMOC and freshwater content time series in revised Figure 6a,b, motivated by the CESM analysis (e.g., revised Figure 4).

Note that the freshwater forcing parameter E_A is prescribed to decrease linearly with ocean warming, not the atmospheric temperature directly. This introduces a warming-rate dependence of the effective freshwater forcing as a function of atmospheric temperature. Thus, while this choice is a strong idealization and does not include e.g. sea ice effects mentioned by Hankel (2025), it demonstrates that a simple state-dependent freshwater forcing alone is sufficient to facilitate rate-dependnet tipping under warming.

Specific comments:

1. *Lines 85–97: why not start from a regular PI climate without any hosing? While I understand it may be useful to start from an AMOC state that is closer to its tipping point, this means that all the simulations start from a climate that may not be realistic/representative of true preindustrial conditions.*

   Multiple experiments have been conducted with the CESM without any freshwater flux forcing ($\overline{F_H} = 0$ Sv); however, the AMOC does not collapse (e.g., Bonan et al., 2022, https://doi.org/10.1175/JCLI-D-21-0912.1). This is likely related to the fact that the AMOC is initially in a monostable regime (Figure 1a) due to persistent freshwater biases. We agree that the imposed F_H is not realistic and should be interpreted as a bias correction for AMOC stability. The discussion has been extended accordingly in the revised manuscript.

2. *Methods: Were the statistical PI steady states of the AMOC (at FH=.18 Sv and .45) obtained by branching from the gradual hosing experiment (van Westen & Dijkstra, 2024) and continuing the experiment with those hosing values fixed in time for a while, in order to ensure that the AMOC is indeed in equilibrium with the hosing value? While the*

hosing rate of increase in van Westen & Dijkstra 2024 is indeed very slow, the circulation could still be modestly out of equilibrium with this very.

Correct, these PI steady states were integrated for 500 years under constant PI and $\overline{F_H}$. For the steady states, the radiative imbalance at the top of atmosphere (TOA) is close to zero. This is now included in the revision.

3. *Line 105: How do the authors define "tipping point" here, when they say that the AMOC reaches a tipping point in a given year? In my view, I would not necessarily assume that the AMOC collapse in the RCP scenarios is a "tipping point" for a few reasons: 1) while the previous work by the same authors shows that the AMOC has multiple equilibria with respect to freshwater forcing, we do not known that it has multiple equilibria with respect to greenhouse gas forcing, 2) relatedly, the collapsed state that is found in RCP4.5 and 8.5 may not be a permanent stable steady state, as much work has shown the AMOC can still recover after several centuries in CESM and other models (without any forcing changes; Bonan et al, 2022; Nobre et al., 2023; Curtis & Fedorov, 2024), and 3) even if the AMOC does undergo a tipping point in the RCP scenarios, it is not clear to me how one would define when this happens, as the circulation is surely not in equilibrium with the forcing (a point the authors make later). It would be best to use careful language here that acknowledges this, and perhaps define this as an AMOC collapse but not necessarily a tipping point.*

   We agree with the reviewer that the crossing of an AMOC tipping point cannot be assessed from transient climate change conditions, as this would require additional (quasi-)equilibrium experiments. In fact, our results suggest that no such tipping point threshold exists in CO_2 forcing space up to $4\times CO_2$. We therefore use the term 'AMOC collapse' instead. This clarification, along with the relevant references, has been included in the revised manuscript. We have also added a short description in the revised Methods section of how the timing of the AMOC collapse onset is estimated.

4. *Line 107: Why is the global warming threshold for the AMOC tipping point higher for RCP8.5 than for RCP4.5 if the AMOC should be even further from tracking its equilibrium in RCP8.5 (since greenhouse gases*

change faster in this scenario)? I would expect, based on the authors' conclusions later in the manuscript, that the faster the forcing rate of change, the lower the global warming tipping point threshold.

It is not entirely clear why the GMST anomaly is higher for RCP8.5 than for RCP4.5, but it is most likely related to differences in radiative forcing agent pathways. This is now included in the revision.

5. *Line 119-121: This is quite peculiar/counterintuitive–can the authors briefly explain why the logarithmic relationship between CO2 concentrations and GMST fails in this case (or cite other literature that does)?*

   These sentences were confusing and rewritten in the revision.

6. *Line 125–126: The RCP scenarios also have other forcing agents changing (other GHGs, aerosols, etc.). I recommend the authors discuss these differences briefly and whether they would be expected to significantly influence the AMOC weakening–if so, then we wouldn't necessarily expect the AMOC to collapse at the same GMST in the CO2 as in the RCPs.*

   Agreed, this is now discussed in the revision and addressed using the faster CO_2 ramps.

7. *Figure 3: Why do the quantities $\Psi_{\rm surf}$ etc in the legend of panel (a) have delta symbols in front of them? This would suggest to me that these quantities are differences from some baseline, but I believe what is shown in this panel are the absolute surface WMT quantities. In Panel (d), conversely, I wonder if these legend labels should have delta symbols in front since what is shown are differences compared to the first 50 years of the simulation.*

   The Δ was confusing here and we used a slightly different naming convention in the revision.

8. *Line 164–169: These lines describing the causes of the NADW changes are a bit confusing, and I suggest the authors re-write them for clarity. I think the main point they should be trying to make is that $\Psi_{\rm NADW}$ can be influenced by changes in surface buoyancy fluxes and changes in the surface area of isopycnals outcropping with densities greater than $\sigma_2^{\rm max}$. The strengthening of $\Psi_{\rm NADW}$ appears to be contributed to by both terms, with surface buoyancy fluxes becoming more negative (Fig 3c)*

and the area meeting the density threshold also increasing Fig. S1 (unless the sign of buoyancy fluxes is different than what I think, in which case the authors should clarify). This understanding is somewhat obfuscated by the fact that the authors say "surface buoyancy fluxes change accordingly to maintain an adiabatic AMOC", which to me implies that surface fluxes are compensating for a change in $> \sigma_2^{\max}$, *when in fact, the two factors are working in the same direction. Relatedly, the authors comment on* $\sigma_2^{\max}$ *and the surface densities changing at the same rate, but don't comment on the area greater than* $\sigma_2^{\max}$, *which is what actually matters for calculating* Ψ_{NADW}.

Agreed, this was not very clear. We have rewritten these sentences accordingly.

9. *Lines 172–173: The RCPs and the CO2 ramping experiment have not only different rates of forcing change, but also different levels of forcing change and different forcing agents. Therefore, it is not actually clear that the disappearance of surface isopyncals* $> \sigma_2^{\max}$ *that explains the collapse of* Ψ_{NADW} *in RCP4.5/8.5 is purely a "rate-dependent" effect, but rather could be related to differences in the magnitude or structure of the forcing.*

   This is now being addressed with the faster CO_2 ramps.

10. *Conventional thought is that net freshwater fluxes in the North Atlantic should increase under global warming, primarily due to an enhancement of the hydrological cycle that would amplifying the global pattern of P-E (e.g., . Why is there a decrease in FW fluxes in this experiment?*

    The hydrological cycle indeed intensifies, but part of these freshwater anomalies leaves the Atlantic basin through atmospheric bridges, resulting in a net decline in surface freshwater fluxes. This issue has been clarified in the revised manuscript.

11. *Lines 221-226: The authors don't show or discuss similar watermass transformation for the RCP scenarios (aside from the densities shown in figure S1), so I feel that the claim that their result shows clear "indications of rate-induced effects" is not justified. For example, the RCPs may have completely qualitatively different evolutions of Bflux (not just different timescales of changes in Bflux) that give rise to greater AMOC weakening at a lower GMST: this would then undermine the assertion*

that "rate-induced" effects are the primary reason for differing AMOC responses. (This is related to general comment #2)

As noted in the manuscript, these results were previously presented in van Westen et al. (2025, https://doi.org/10.1029/2025JC022651). However, we agree with the reviewer that including the RCP results improves the completeness of the analysis. We have therefore added 'summary figures' that show the key RCP findings (e.g., revised Figures 3g,h), which are qualitatively and quantitatively very similar to the CO_2 ramps.

12. *Line 255–256: Again, I don't think the claim that "AMOC tipping under anthropogenic climate change is primarily controlled by the warming rate" is fully supported by the evidence in this manuscript. It is clearly influenced by the warming rate, but, given that the different RCPs also have different forcing magnitudes and forcing agents, and that the AMOC in RCP8.5 collapses at a higher GMST threshold than in RCP4.5, one could just as easily conclude that AMOC weakening is governed by the spatial structure and temporal evolution of surface forcings, which are influenced by all of the above factors (not just the warming rate).*

    This is now being addressed with the faster CO_2 ramps.

13. *Paragraph starting at Line 292: I'm a bit confused about what the authors are trying to say here about salinity changes at 34S, mostly because the figures seem to contradict each other. In Figure 4d, the near-surface salinity at 34S seems to be decreasing, and the authors point this out in Line 292 and mention that it is surprising. However, Figure 5a shows a clear surface salinity signal at 34S, and the authors say that the Atlantic basin is salinifying, which seems to contradict Figure 4d? Moreover, the authors try to connect Atlantic salinity changes to changes in other basins (Pacific basin, Indian Ocean via Indian throughflow and Aghulas Leakage), but these explanations are purely speculative, as the authors do not perform any analysis that shows interbasin freshwater pathways, either oceanic or atmospheric (this would be quite challenging to do). I recommend the authors remove this speculative text and instead focus on the local changes occurring at 34S that can explain the changes in Fov.*

    We agree with the reviewer and removed this in the revision.

14. *Lines 325–229: The salinifying NADW in the CO2 and in the low-intermediate RCP scenarios for CESM and CMIP6 models has already been discussed in lines 257–291, so it is not clear why the authors repeat this point here. Since the authors are analyzing surface salinity and freshwater fluxes in this section, perhaps it would be clearer to simply make the point that Figure 5a and b show a strong salinification over the WMT regions in the North Atlantic where NADW is produced, which gives a plausible cause for the salinification of the NADW mass at 34 S that was commented on previously.*

    This entire section was rewritten.

15. *Lines 331—334: "This also means that the destabilizing effects of anthropogenic climate change at high enough forcing rate can become larger than the stabilizing responses, such that the AMOC is brought out of equilibrium and subsequently collapses through a rate-induced AMOC tipping event." I don't believe this statement has been proven based on analysis presented in this paper, or at least, the authors have not made a convincing argument that their analyses show this (see major comment #3).*

    This is now being addressed with the faster CO_2 ramps.

16. *Line 428–430 (and elsewhere): the reduced sea ice extent not only leads to reduced insulation, but (perhaps even more importantly) to reduced freshwater – in fact this is the biggest contribution to the FW flux over the North Atlantic, where it can influence NADW production.*

    Agreed, this is now made more explicit.

17. *Line 444—445: "... we propose to convert the AMOC tipping warming threshold to a warming rate threshold" Again, I do not feel like the analysis presented in this paper justifies this: first, because the authors did not explore the impact of the rate of forcing change systematically, but rather that some mechanisms of AMOC stabilization/de-stabilization are fundamentally different in different forcing scenarios (of different forcing rates, forcing levels, and forcing agents), and second, because the experiments they did analyze don't reveal a straightforward relationship between AMOC collapse and the rate of warming: the AMOC collapsed at a lower GMST for the RCP scenarios that the slow CO2 ramp, but actually collapsed at a lower GMST for the RCP4.5 vs 8.5*

(despite it being a slower forcing rate of change). I think it is safer (and still very impactful!) to say that a pure GMST threshold for AMOC tipping clearly is not meaningful, but that other physics-based indicators may still be useful, but should also be re-evaluated in the context of different forcing rates and scenarios.

This is now being addressed with the faster CO_2 ramps.

MS-No.: NCLIM-26020620

Version: Revision II

Title: Failure to track a stable AMOC state under rapid climate change

Author(s): René M. van Westen, Reyk Börner and Henk A. Dijkstra

Point-by-point reply to reviewers

July 9, 2026

We thank the reviewers again for their careful reading and for the useful comments on the revised manuscript.

Reviewer #1

This is a second review of the manuscript. The authors tried to address my comments and reservations, and I am grateful for this. I believe that the main message of the paper is clear and important to be made, namely that the rate of increasing emissions plays a role in determining the threshold for tipping behavior in the AMOC.

The paper is still too long I think but very worth publishing anyway. I have a few suggestions, which are minor in effort, but important to bring out the message. So please address them if you agree:

Minor comments:

1. *Consider rephrasing the text in your abstract to highlight the important result that there is no single GWL (or CO2) threshold for AMOC collapse because these thresholds are pathway (rate) dependent.*

   We now highlight that there is not a single temperature threshold in the revised abstract.

2. *Likewise, note (as you already say in the text) that the term "tipping point" should be avoided and use "tipping transitions" instead. This should be in the abstract or conclusions too.*

   The term 'tipping point' is avoided in the abstract and discussion.

3. *Please note in your text that Hankel et al used the same model (CESM1).*

   Suggestion followed.

4. *Also, it would be helpful to use warming rates rather than CO2 rates and GWLs in your abstract. For example, you say that the stable AMOC is found in the slow CO2 ramp up case. But what is the warming rate in this run? What is the warming rate in the fast CO2 ramp-up? You can provide average warming rates, I think that would be ok.*

   The different CO_2 ramping rates are preferred in the abstract, as this is the parameter varied across the simulations. Global warming levels (or warming rates) depend on slow climate feedbacks, which are discussed in the main text. No changes have been made to the abstract.

5. *In the table S1 (thank you for including it) could you please list the number of years of imposed forcing (FH), not the model year. I am not sure the model year is informative. Also in a separate column please list an "average" warming rate associated with the CO2 rate. Ideally you would associate it with emissions rate too, but I understand that your simulations are not emissions-driven.*

   As is explained in the main text, the F_H is fixed throughout the climate change simulations, which is being indicated as $\overline{F_H}$. The warming rates display non-linear responses for the collapsing AMOC cases, hence it is not useful to present an average warming rate in Table S1. These rates, however, were mentioned in the main text (lines 163 – 164).

6. *Lastly you might want to highlight in your results that there is also no "system threshold" for AMOC collapse (i.e. a single AMOC strength level after which AMOC is considered collapsed – eg. Jackson et al take 6Sv as such threshold etc). $\psi_{\mathrm{NADW}} = 0$ is a better system-threshold to assess collapse.*

   This point is extensively discussed in van Westen et al. (2025, https://agupubs.onlinelibrary.wiley.com/doi/10.1029/2025JC022651) and we highlighted this now in the Methods.

Reviewer #2

The authors have addressed my concerns well. I think the work can be accepted for publication.

Thank you.

Reviewer #3

Summary: The authors have made significant improvements to this manuscript, and I believe their main conclusions are now much better supported by their analyses. In particular, I appreciate that the authors were able to perform additional simulations with different CO2 increase rates, and I believe that including these results (as well as their more complete analysis of the RCP simulations) alongside the original simulations now convincingly shows that the mechanisms they present are indeed the ones leading to a rate-induced AMOC collapse. In addition, their re-writing of some sections (particularly on the freshwater budget of the Atlantic) has made the manuscript clearer and the logic easier to follow. Their response to my previous comments and re-writing of the Dynamical Perspective section has also strengthened my understanding of the connection between the box model and the CESM results. There are still some areas of the manuscript where the writing requires some clarification, which I provide suggested improvements for in my comments below, but overall, I think this paper should be published.

Comments:

1. *In the "Stable AMOC" section, I suggest mentioning immediately (within the first paragraph, before getting into the details of the simulations) that the motivation for branching from the hosed PI state is to study an AMOC that is in a bi-stable regime (and thus, perhaps, more resembling the Earth's AMOC). As of now, this reasoning is somewhat buried deep in the third paragraph, but it is very important for orienting the reader to the experimental design.*

   Agreed, this is now made more explicit in this paragraph.

2. *Calculation of ψ_{NADW} and related contributions: Is ψ_{NADW} calculated as the total surface WMT for all density classes greater than $\sigma_2^{\max}$ (as lines 193/194 suggest), or is it simply the value of ψ_{surf} at $\sigma_2^{\max}$ (as the notation $\psi_{\mathrm{NADW}} = \psi_{\mathrm{surf}}\ (\sigma_2^{\max})$ in line 197 suggests), i.e., essentially just looking at the maximum value of ψ_{surf}? I think, based on the following the discussion, that it is the former, but I recommend adding/standardizing the notation in lines 193—198 to make this more explicit – as it written now, it's not clear that the "water supply of*

the North Atlantic Deep Water (NADW)" is the quantity ψ_{surf}, and I found it confusing that the equation $\psi_{\text{surf}} = \psi_{\text{surf}}$ (σ_2^{max}) drops the the σ_2¿ σ_2^{max}.

The definition of $\Psi_{\text{NADW}}(t)$ is defined as the WMT difference between σ_2^{max} and σ_2^{∞}, with Ψ_{surf} at σ_2^{∞} being zero (see Figure 2a). Hence, it simply reduces to: $\Psi_{\text{NADW}}(t) = \Psi_{\text{surf}}(\sigma_2^{\text{max}}(t))$. This is now clarified in the main text.

3. *Assuming ψ_{NADW} is indeed calculated for all density classes greater than σ_2^{max}, then it is influenced by two factors: the area of $\sigma > \sigma_2^{\text{max}}$, and the changing Bfluxes. The former can, in turn, be further broken down into changes in surface isopycnal outcropping (σ_2) and changes related to interior ocean density (σ_2^{max}). By the end of the section, I was able to understand the relative contributions of these three factors, but I found that the way the authors phrased some things was a little bit confusing, and it may help if they try to streamline/re-order lines 202-224 to reflect this very intuitive breakdown. For example, this breakdown is sort of muddled when the authors say: "this combined response (that ψ_{NADW} remains large while σ_2^{max} decreases) means that both the interior and surface sinking region are getting lighter at the same rate…". In fact, that ψ_{NADW} remains large (actually increases) while σ_2^{max} decreases could be from either of the two other factors: that surface densities in the sinking region are also getting lighter, OR that Bfluxes are getting more negative. Later, they show that indeed the ψ_{NADW} changes are because of both these things, so I think this sentence is somewhat misleading. I suggest the authors try reorganizing these few paragraphs so that the logic is more coherent. For example, rather start with focusing on σ_2^{max}, they could start by describing that $\sigma_2 > \sigma_2^{\text{max}}$ (the quantity that goes into the calculate of ψ_{NADW}) actually increases, and then reveal that this increase can be attributed to the fact that while surface densities are getting lighter in the sinking region, σ_2^{max} is also getting lighter at a comparable rate (but this would not be expected under fast forcing changes).*

   Agreed, we have streamlined the text by following the reviewer's suggestions.

4. *Paragraph starting on line 246: the role of new deep convection areas developing due to sea ice loss is quite interesting, but I would not nec-*

essarily expect this to be a rate-dependent effect (since presumably all experiments lose their Lab. Sea ice). Does this mechanism occur in the faster ramping/RCP scenarios, and if not, why?

The sea-ice responses are rate-dependent and this is illustrated in Figure 2 of Hankel (2025, https://www.pnas.org/doi/epub/10.1073/pnas.2411357121). The slow CO_2 ramp allows for a gradual retreating sea-ice extent between 40°N – 65°N (inset Figure S2b), while in the faster ramps the sea-ice extent is temporarily increasing (insets Figures S2d,f). We mention the sea-ice rate-dependence in the revised manuscript.

5. *Line 298-299: I'm a bit confused by the sentence "The surface flux Fsurf is slightly declining prior to the strong increase in Fov, which implies that the salt-advection feedback is triggered by heat fluxes." How does this conclusion follow? Are the authors trying to say that surface freshwater fluxes cannot trigger the initial AMOC decline because they are going in the wrong direction?*

   Correct, there is no freshwater convergence by the surface component, which is also being illustrated by the B_{flux} decomposition (Figure S2). We have rewritten these sentences in the revision.

6. *Figure 5 caption: It would be good to add early in the caption that these subfigures are all for the gradual CO2 ramp experiment.*

   Suggestion followed, this is now included in the Figure's summary (bold text).

7. *Line 300: "dominant" – dominant compared to/over what? Do you just mean it's large compared to the slow CO2 ramp?*

   We have rewritten this sentence.

8. *Line 337: What is "the latter" referring to?*

   We have rewritten these sentences to clarify this.

9. *Discussion of NADW salinification: Starting around line 348, the writing here makes the connection between analyzing FovS and NADW a bit unclear (in particular, I don't understand the phrase "allowing the interhemispheric salinity contrast to adjust"). Rather than claiming that*

increased NADW salinity is a "fingerprint" of increased AMOC stability, it might be clearer to say that you're analyzing the main contributions to the increasing FovS (which the authors have already established can be understood as an indicator of AMOC stability). The main contributions could be freshening of the upper ocean at 34S or salinification of the NADW – the former is less consistent across models, so you conclude that salinifying NADW is a main cause of a stabilizing climate, and is expected from increased net evaporation in the North Atlantic. Relatedly, it seems important to say somewhere in this paragraph (and later when concluding the section too, see point #11) that this stabilizing process can only occur under sufficiently slow forcing changes, since it requires GMST to adjust and for the salinification signal to propagate all the way to 34S at depth. This is the key intuition for why you see this signal under slower forcing scenarios, but I'm not sure if you say it anywhere explicitly.

We have rewritten this part in the revision. The salinity anomalies that are formed in the northern North Atlantic Ocean (Figure 4b) eventually leave the Atlantic basin at 34°S, with the fastest pathway via the deep western boundary current (DWBC). We have determined the salinity anomalies over the DWBC at 34°S, which indicates a monotic increase (revised Figures S6c,d). The salinity across the entire zonal extent takes longer to adjust (Figure 5d), explaining why these salinity anomalies become more pronounced after model year 700 (Figure 5d). Following the reviewer's suggestion, we also highlight the propagation timescale in the section's summary.

10. *Lines 356–360: I found the discussion of +2.5C as a sort of threshold that delineates between experiments' NADW behavior to be odd, as there's no physical reason that the salinification of NADW should exist/not exist above/below this threshold. Rather, since P-E changes should scale somewhat linearly with GMST, wouldn't you expect a smooth range in the strength of the NADW salinification signal that scales with the range of GMST changes?*

    The salinity over the DWBC at 34°S clarifies this point (revised Figures S6c,d), as it shows a much smoother range for increasing GMST. The results and related text are changed accordingly.

11. *Related to point #9 above: Overall, this section is a lot easier to fol-*

low than it was in the first draft, but I still found it very long and challenging, and by the end, it was not entirely clear what the primary mechanism related to the particular analyses of this section was that could help explain the rate-dependent AMOC collapse. I would encourage the authors to consider finding paragraphs to shorten/remove, and also to conclude this section by summarizing/highlighting which aspects of the freshwater budget mechanisms clearly depend on the forcing rate. For example, they say rather vague summary statements like "the rate-dependent destabilizing effects of anthropogenic forcing must overcome the slow stabilizing effects", but what are those stabilizing/destabilizing effects, and why do they depend on the forcing rate (again, specific to the analysis in this section)? I think the main answer to this is that surface salinification of the (north) Atlantic can stabilize the AMOC by increasing FovS, but this occurs on a slow timescale because it requires (a) GMST to adjust to radiative forcing (in order to enhance net evaporation) and (b) the surface salinity signal to propagate downwards in the North Atlantic and salinify the outgoing NADW at 34S. Under fast radiative forcing changes, net surface heat fluxes weaken the AMOC and kick off the salt-advective feedback before this stabilizing process can happen (and, net freshwater fluxes are not counteracting this as much as they are in the slow ramp). Hopefully the authors can highlight and clarify this at the end of the section, because as it stands, the rate-dependent nature of this stabilizing feedback is not made very explicit.

We have streamlined this part of the manuscript (see also point #9).

12. *The Dynamical Perspective is much more effectively written now, and I thank the authors for clarifying that the effective precipitation depends on the North Atlantic ocean temperature, not the imposed atmospheric temperature (I misunderstood this previously).*

    Thank you!
